# A modular chromosomal passenger complex rewires chromosome segregation in *Plasmodium berghei*

**DOI:** 10.1038/s41467-026-74542-7

**Published:** 2026-06-20

**Authors:** Magali Roques, Chengyue Niu, Mathieu Brochet, Lorenzo Brusini

**Affiliations:** 1 Institute of Cell Biology, University of Bern, Bern, Switzerland; 2 Department of Microbiology and Molecular Medicine, Faculty of Medicine, University of Geneva, Geneva, Switzerland

**Keywords:** Parasite biology, Parasite evolution, Chromosome segregation, Checkpoints

## Abstract

Faithful chromosome segregation relies on precise kinetochore-microtubule interactions and checkpoint surveillance, yet the molecular basis of these processes varies widely across eukaryotes and is only beginning to be defined in apicomplexan parasites. In the malaria parasite *Plasmodium berghei*, chromosome segregation is especially critical during transmission from host to mosquito: rapid mitoses generate male gametes, and meiosis in the zygote seeds the next round of infection. Here, we identify Aurora-related kinase 1 (ARK1) as a central regulator of chromosome segregation in both mitotic and meiotic contexts. ARK1 localises to spindle poles, spindles, and kinetochores, and its depletion results in short and multipolar spindles, kinetochore misalignment, and failed chromosome partitioning. ARK1 forms a minimal Chromosomal Passenger Complex (CPC) with INCENP-A during male gametogenesis, but associates with additional components, including INCENP-B, kinetochores, centromeric histones, and spindle assembly checkpoint proteins, during meiosis. This stage-specific modularity supports efficient male gamete formation while safeguarding faithful chromosome inheritance during zygote development, thereby ensuring parasite transmission to the mosquito. Together, our findings indicate that *P. berghei* deploys distinct CPC states across sexual development, revealing developmental plasticity in chromosome-segregation control and a potential vulnerability for blocking transmission.

## Abstract (web summary)

This study shows that malaria-causing parasites use a unique chromosomal passenger complex with stage-specific composition, revealing how eukaryotic chromosome segregation can be rewired during parasite transmission.

## Introduction

Faithful segregation of eukaryotic chromosomes during cell division depends on their attachment to the microtubule-based spindle via kinetochores^1^. During mitosis, duplicated chromosomes are segregated precisely once to each daughter cell, whereas the production of haploid gametes requires two consecutive meiotic divisions, with homologous chromosomes segregated in meiosis I and sister chromatids separated in meiosis II^2^. In well-studied animal and fungal systems, fidelity of chromosome segregation depends upon biorientation at metaphase, where sister kinetochores attach to microtubules emanating from opposing spindle poles^3^. Biorientation is assured through an error-correction process: incorrect kinetochore-microtubule attachments trigger a “wait” signal via the spindle assembly checkpoint (SAC), a surveillance system that delays the onset of anaphase until all chromosomes are correctly attached^4^. Correction of erroneous kinetochore-microtubule attachments is coordinated by antagonism between centromeric kinases and outer kinetochore phosphatases^5^. A central component of this control is Aurora B kinase within the chromosomal passenger complex (CPC), composed of Aurora B, INCENP, SURVIVIN and BOREALIN, which destabilises improper attachments and promotes checkpoint signalling early in mitosis^6^. Upon biorientation, tension exerted across sister kinetochores is communicated through the action of PPP1 and PP2A phosphatases, leading to checkpoint silencing and timely progression into anaphase^7^.

SAC control varies greatly across eukaryotes. While the requirement for chromosome biorientation is widespread, the composition of SAC pathways and the mechanisms by which kinetochores communicate attachment status are not universally conserved^8–11^. Parasites belonging to the phylum Apicomplexa illustrate this diversity particularly clearly. This phylum groups a large number of intracellular parasites of considerable medical and veterinary relevance, including the malaria parasite *Plasmodium* and *Toxoplasma*, the causative agent for toxoplasmosis. In *Plasmodium* and *Toxoplasma*, conserved kinetochore components have now been identified, revealing recognisable inner and outer kinetochore modules^12,13^. Furthermore, recent ultrastructural work in *Plasmodium falciparum* showed that disruption of the NDC80 complex destabilises coupling between the mitotic apparatus and centrosome-equivalent structures during asexual blood-stage divisions, highlighting conserved kinetochore–spindle–MTOC connectivity during parasite division^14^. However, SAC proteins (beyond a putative MAD1 homolog) and key kinases required for SAC recruitment, such as Monopolar Spindle1 and Polo-Like, appear to be missing^15^, and the lack of complete metaphase arrest in response to certain spindle poisons has questioned whether these parasites support a robust error-correction response^16,17^. Comparative genomics further suggests that the CPC in *Plasmodium* and *Toxoplasma* is reduced, lacking clear orthologs of SURVIVIN and BOREALIN and containing two INCENP paralogs^18^. Together, these observations argue not for the absence of chromosome-segregation control, but for a streamlined regulatory system whose molecular organisation and deployment remain poorly resolved.

Sexual development in *Plasmodium* provides a particularly powerful context in which to investigate plasticity in chromosome-segregation control. Following transmission from vertebrate host to mosquito vector, male gametocytes undergo rapid rounds of DNA replication and mitosis to generate microgametes^19^, whereas fertilisation is followed by meiotic divisions during zygote development^20^. These stages differ markedly in tempo, division architecture and outcome, and represent critical transmission bottlenecks in the parasite lifecycle: failure to complete them faithfully directly compromises parasite transmission to the mosquito vector. Previous work established metaphase-like kinetochore alignment during asexual blood-stage mitosis and during meiosis, whereas similar architectures during male gametogenesis remain less clear^13^. This raises the possibility that *Plasmodium berghei* does not employ a single chromosome-segregation regime across its lifecycle.

Here we investigate chromosome segregation during sexual development in *Plasmodium berghei*. We show that the Aurora-related kinase ARK1 associates with spindle poles, spindle microtubules and kinetochores, and is required for bipolar spindle formation, kinetochore alignment and faithful chromosome segregation. Importantly, our results reveal a modular CPC whose composition shifts between mitosis of male gametogenesis and meiosis of developing ookinetes. This stage-specific modularity correlates with distinct modes of chromosome segregation, supporting efficient male gamete formation alongside tighter safeguarding of chromosome inheritance during zygote development. Together, these findings indicate that, whilst the core principles of kinetochore–spindle coupling are conserved in *Plasmodium*, CPC composition and attachment-regulatory mechanisms are developmentally modulated, broadening our understanding of the plasticity of eukaryotic chromosome-segregation control.

## Results

### MTOC partitioning drives spindle assembly and kinetochore redistribution during microgametogenesis

Sexual development in *Plasmodium berghei* is initiated by a change in conditions from host to mosquito following a blood meal. Male gametocytes (microgametocytes) undergo three rounds of DNA replication and mitosis within minutes, producing eight flagellated male gametes. Male gametes fertilize female gametes (macrogametes), leading to meiosis in the zygote and subsequent differentiation into motile banana-shaped ookinetes.

**Fig. 1:**
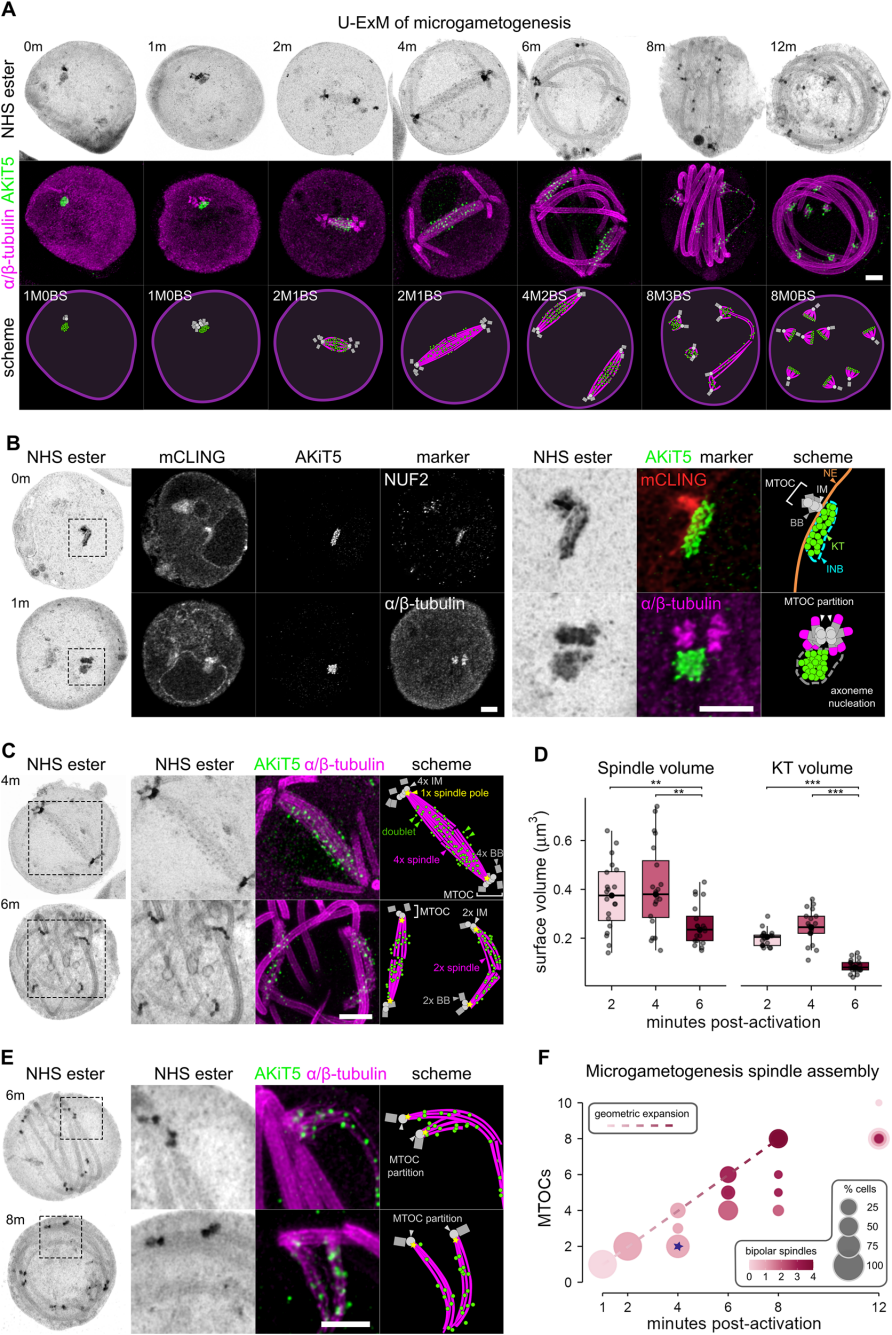
MTOC partitioning drives spindle assembly and kinetochore redistribution during microgametogenesis. **A** Ultrastructure expansion microscopy (U-ExM) time course of activated *Plasmodium berghei* microgametocytes (0–12 min post-activation; m.p.a.). Grayscale panels show NHS-ester labelling; merged panels show α/β-tubulin (magenta) and AKiT5-mNeonGreen-3×HA (green; detected by anti-HA). Representative images are from full-cell 3D reconstructions spanning three independent biological replicates. Schematics summarise the inferred numbers of microtubule organising centres (MTOC, M) and bipolar spindles (BS). **B** Early activation (0 and 1 m.p.a.) showing the bipartite MTOC and its relationship to the nuclear envelope. mCLING marks membrane (red), NHS-ester marks the MTOC region (grayscale), AKiT5 and NUF2 mark kinetochores, and α/β-tubulin marks axoneme/spindle microtubules. IM, inner MTOC; BB, basal body; NE, nuclear envelope; INB, inner nuclear body; KT, kinetochore. **C** Example at 4 m.p.a. showing an elongated bipolar spindle with laterally distributed AKiT5 puncta doublets. (**D**) Volumetric measurements from 3D reconstructions showing spindle microtubule volume and kinetochore (KT) volume across time points. *n* = 19, 21, and 20 fully reconstructed microgametocytes at 2, 4, and 6 m.p.a., respectively, from three independent biological replicates. Boxplots show the median centre line, interquartile-range box, and 1.5×IQR whiskers; points are individual cells. Statistical significance was assessed using two-sided Wilcoxon rank-sum tests with no adjustment for multiple comparisons; exact *P* values are provided in the Source Data file. **E** Examples at 6 and 8 m.p.a. illustrating continued MTOC partitioning and emergence of additional bipolar spindles. **F** Population quantification of spindle assembly states across time. Bubble size indicates the fraction of cells in each state, and colour indicates the number of bipolar spindles. The dashed line indicates the idealised geometric doubling trajectory. Scale bars, 1 μm unless otherwise indicated; dimensions are non-expanded. Source data are provided as a Source Data file.

Microgametogenesis does not appear to follow the same sequence of mitosis as described in animal cells, and how chromosomes are captured, partitioned, and faithfully inherited into male gametes is not completely understood. In particular, microgametocyte mitosis appears to lack clear kinetochore alignment, linear structures formed by apicomplexan kinetochore proteins (AKiTs) as observed during asexual blood-stage mitosis and highly reminiscent of biorientation (Figure S1A, B^13^). To better describe chromosome segregation during microgametogenesis, we tracked the spindle microtubule organising centre (MTOC), bipolar spindles and kinetochores at discrete time points post-activation using Ultrastructural Expansion Microscopy (U-ExM; Fig. 1A and Supplementary Data 10). The apicomplexan kinetochore protein 5 fused at the C-terminus to mNeonGreen-3xHA (AKiT5-mNG-3xHA; PBANKA_1243900) has previously been validated as a bona fide inner kinetochore component and shown to co-localise with established kinetochore markers^13^, which we also confirm here (Figure S1A–B).

In the non-activated microgametocyte, kinetochores occupy the inner nuclear body^21^ adjacent to a microtubule organising centre (MTOC) embedded at, or tightly associated with, the membrane of the nuclear envelope (Fig. 1B; “0 m”; Figure. S1C). Although the MTOC appears amorphous by NHS-ester staining, ultrastructural studies have shown it contains two tethered nucleation modules: a tetrad of basal bodies (denoted “BB”) from which axonemes extend, each linked across the nuclear envelope to an intranuclear site (the inner MTOC, denoted “IM”) that seeds spindle microtubules^22,23^.

Following activation, the MTOC undergoes a striking stepwise partitioning programme. By 1 min post-activation, it has resolved into two opposing plates, each comprising a tetrad of inner MTOCs and associated with axoneme nucleation on the cytoplasmic side of the nuclear envelope (Fig. 1B, “1 m”; Fig. S1D). By 2–4 min, the first intranuclear spindle elongates between these MTOC units as they move apart across the nucleus (Fig. 1C, “4 m”; Figure. S1D). At this stage, kinetochores do not form an equatorial plate; rather, they appear as paired puncta distributed laterally along spindle microtubules (Fig. 1C and Figure S1D, E), consistent with earlier observations^13^.

Between 4 and 8 min post-activation, MTOC partitioning continues and is accompanied by the emergence of additional bipolar spindles. Consistent with ultrastructural observations^21^, quantification of 3D reconstructions shows that spindle microtubule volume decreases between successive rounds of division, coincident with an ~2-fold decrease in kinetochore volume and progressive subdivision of the original MTOC into smaller units, culminating in singlets following the terminal division (Fig. 1D, E, Figure. S1D–F and Supplementary Data 1). Consequently, it remains unclear whether the first division proceeds with a diploid spindle–kinetochore complement or whether the first spindle engages a larger set of kinetochores.

Population-level quantification of MTOC units and bipolar spindles reveals a stepwise doubling (geometric progression) of spindle assembly (Fig. 1F; dotted guide line). However, at 4 min most cells dwell disproportionately in the single-spindle state (Fig. 1F; blue star), with others already reaching three or four separate MTOC units (Figure S1D; “4 m”), revealing MTOC partitioning precedes the emergence of additional bipolar spindles.

By 12 min post-activation, spindle microtubules retract, and eight discrete kinetochore clusters are present, one for each emerging male gamete (Figure S1G). Together, these data indicate that microgametogenesis proceeds through progressive MTOC partitioning and kinetochore redistribution along spindle microtubules, raising questions about whether it follows a sequential duplication–elongation–split programme in the absence of metaphase-like biorientation.

### Paternal chromosome transmission is less faithful during sexual development

Given the apparent absence of kinetochore congression and alignment, cues typically associated with correction of erroneous kinetochore-spindle interactions in many eukaryotes^24–29^, we asked whether inheritance of paternal chromosomes remains fully faithful during transmission.

**Fig. 2:**
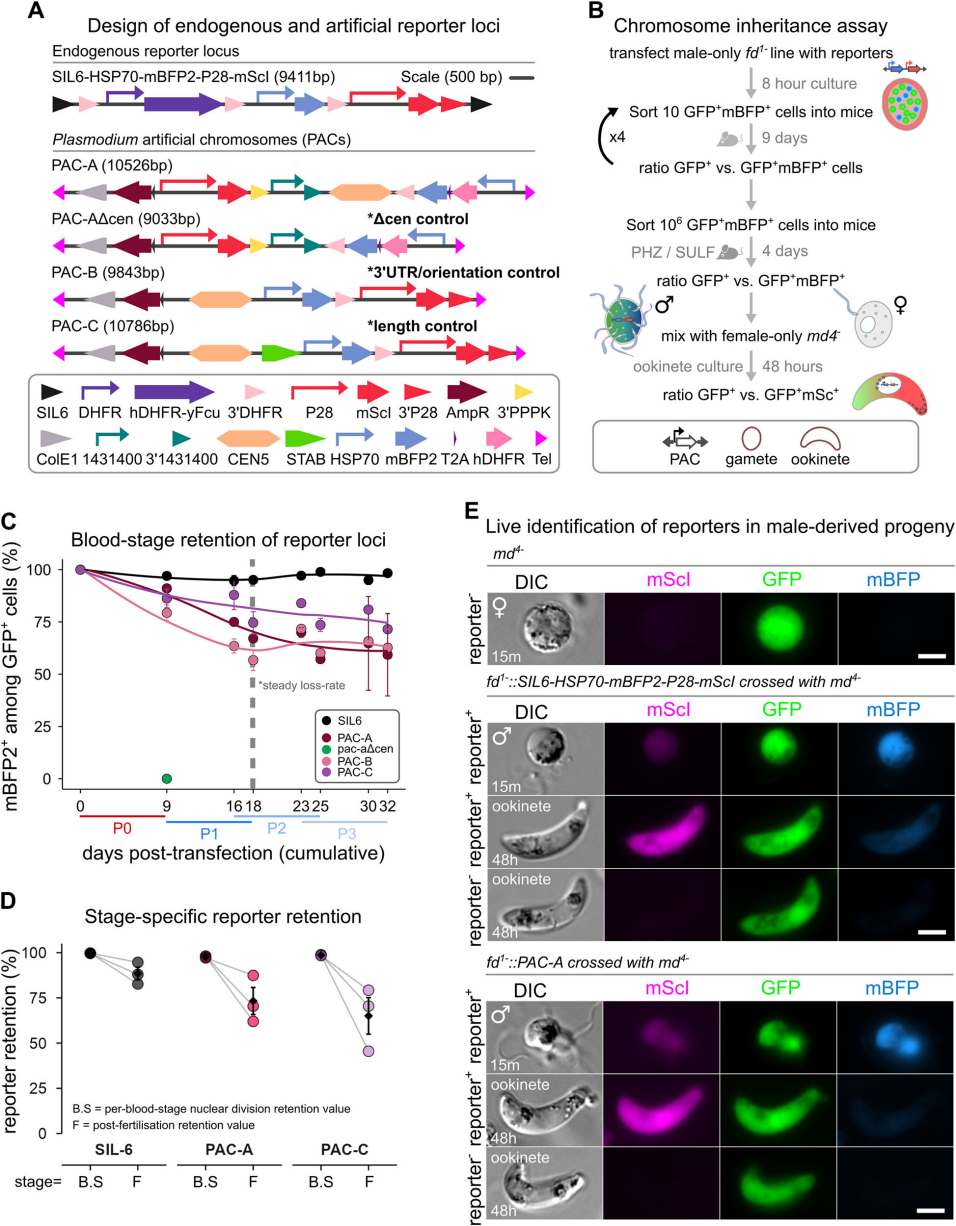
Paternal chromosome transmission in the zygote is less faithful than chromosome maintenance during blood-stage proliferation. **A** Schematic of the endogenous *SIL6* reporter and *Plasmodium* artificial chromosome (PAC) constructs used to monitor chromosome inheritance. The SIL6 reporter carries HSP70-driven mBFP2 and P28-driven mScarlet-I. PAC-A contains telomeric repeats, centromere 5 and the same reporter logic; PAC-AΔcen lacks centromere 5; PAC-B reorients the reporter cassettes and replaces the PPPK 3′UTR with the endogenous P28 3′UTR; PAC-C contains an additional STAB sequence upstream of HSP70. **B** Chromosome-inheritance assay. Reporter constructs were introduced into male-only *fd*^*1−*^ parasites, followed during serial blood-stage propagation by flow cytometry, and crossed to untransformed female-only *md*^*4−*^ parasites after phenylhydrazine treatment and sulfadiazine selection to assess paternal inheritance after fertilisation and ookinete development. GFP identifies the *fd*^*1−*^*/md*^*4−*^ lineages; mBFP2 and mScI report retention of the marked locus. **C** Blood-stage retention of reporter loci, measured as the percentage of GFP^+^ cells that are also mBFP2^+^. P0–P3 indicate passages. Points show individual biological replicates, and coloured lines connect replicate means. *n* = 4 independent biological replicates per reporter line unless otherwise indicated; each independently passaged parasite population is the unit of analysis. Error bars show mean ± SEM. The dashed line marks the approximately linear loss phase used to estimate steady-state maintenance. **D** Reporter retention during blood-stage propagation compared with after fertilisation. Lines connect matched biological replicates. Blood-stage values were calculated from reconstructed late-phase cumulative retention, with day 18 normalised to 1, and converted to retention per nuclear division, assuming four nuclear divisions per 24 h cycle. Post-fertilisation values indicate paternal reporter retention in mature ookinetes. *n* = 3 matched independent biological replicates per reporter line unless otherwise indicated; each independently passaged and crossed parasite population is the unit of analysis. Error bars show mean ± SEM. **E** Representative live images of gametocytes and ookinetes after the indicated crosses. Scale bar, 2.5 μm. Source data are provided as a Source Data file.

To directly assess chromosome inheritance in *P. berghei*, we established a chromosome inheritance assay using *Plasmodium* artificial chromosomes (PACs) together with an endogenous chromosomal reporter (Fig. 2A, B). PAC-A carries telomeric repeats and centromere 5 together with two reporters: *HSP70*-driven mBFP2 to monitor retention during blood-stage proliferation, and *P28*-driven mScarlet-I to score inheritance after fertilisation and ookinete development. Three additional controls were generated: PAC-Aδcen, an acentromeric control; PAC-B, in which the reporter cassettes are reoriented relative to the centromere and the mScI PPPK 3′UTR is replaced with P28 3′UTR; and PAC-C, a longer-context control that includes ~1 kb of genomic DNA (STAB) upstream of the *HSP70* promoter. As an endogenous reference, the same HSP70-mBFP2/P28-mScarlet logic was integrated at the *SIL6* locus.

To specifically monitor paternal chromosome segregation, each reporter was introduced into the male-only *fd*^*1-*^ line, which constitutively expresses *GFP* from the *SSU* locus under control of the *eEF1α* promoter^30^, and crossed to untransformed female-only *md*^*4-*^ parasites (Fig. 2B). mBFP2 and mScI measure inheritance of the reporter locus itself, whereas GFP identifies the *md*^*4*−^ and *fd*^*1*−^ lineages (Figure S2A, B and Supplementary Data 2). During serial blood-stage propagation, the acentromeric PAC-AΔcen control was rapidly lost, confirming the requirement for a centromere. By contrast, centromere-containing PACs were retained at high frequency, with estimated maintenance rates close to 98% per nuclear division, although they gradually declined relative to the endogenous *SIL6* control during the early post-transfection period (Fig. 2C, D). After ~18 days of propagation, this decline stabilised into a near-linear loss across subsequent passages. Previous work has shown that shortly after transfection, individual parasites can contain multiple PAC copies, which are progressively lost as parasites converge towards single-copy states^31^. Although we did not directly measure PAC copy number in our lines, the transition to a stabilised linear loss phase is consistent with the population having largely converged to single-copy PACs. PAC-B behaved similarly to PAC-A, indicating that cassette orientation and 3′UTR configuration are not the main determinants of blood-stage maintenance, whereas PAC-C showed slightly improved retention, suggesting that reporter length or surrounding sequence context may further stabilise inheritance.

When these reporters were followed through fertilisation, inheritance was markedly less robust. Even the endogenous *SIL6* reporter was not fully retained (90.44 ± 3.46%) in ookinetes, showing that paternal chromosome transmission is not fully faithful even for endogenous chromosomes (Fig. 2D, E). PAC-A and PAC-C showed substantially reduced retention after the male-to-ookinete transition compared with their inferred blood-stage retention. In mature ookinetes, the P28-driven reporter provided a clear readout of reporter-positive and reporter-negative progeny, whereas the HSP70-driven mBFP2 PAC signal was weak or undetectable, consistent with the known maternal bias of constitutive reporter expression during early zygote and ookinete development. Together, these data indicate that paternal chromosome transmission during fertilisation is substantially less faithful than chromosome inheritance during blood-stage proliferation.

### ARK1 defines a reduced CPC with an INCENP-A core in male gametocytes

The contrast in chromosome-inheritance fidelity between blood-stage proliferation and sexual development prompted us to look more closely at the chromosome-segregation machinery operating during male gametogenesis. Given the critical roles for Aurora kinases during mitosis in eukaryotic cells, we targeted an Aurora-related kinase called ARK1. In *P. berghei*, PbARK1 co-purifies with kinetochore components immunoprecipitated from activated mitotic gametocytes^13^, and the homolog PfARK1, from the human-infecting species *P. falciparum*, has been shown to localise to spindle poles during intra-erythrocytic divisions^32,33^.

**Fig. 3:**
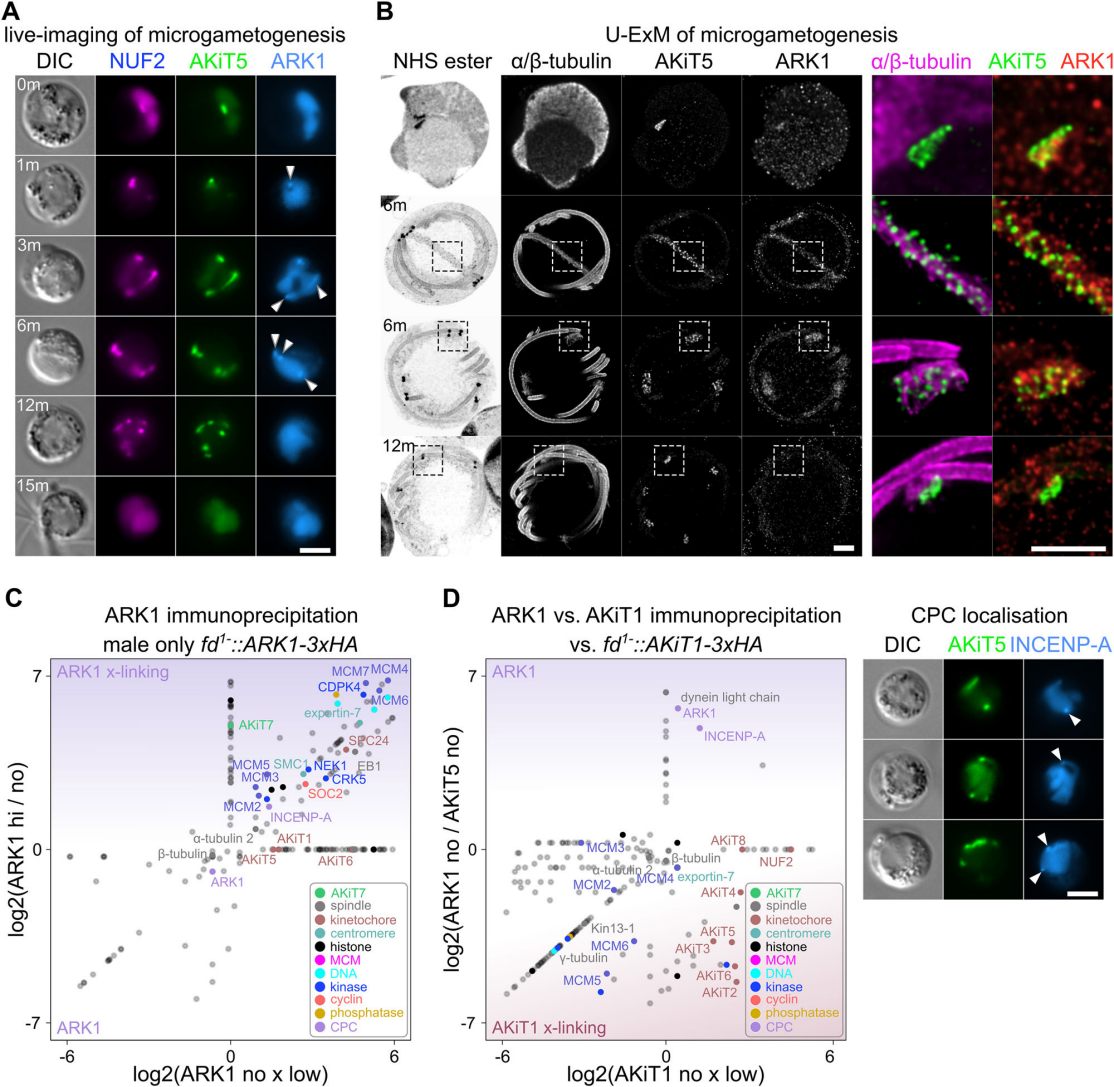
ARK1 forms a minimal CPC with INCENP-A and associates with spindle poles, spindle microtubules and kinetochores during microgametogenesis. **A** Live-cell imaging of native fluorescence in microgametocytes expressing NUF2-mScI (magenta), AKiT5-mNG-3xHA (green) and ARK1-mBFP2-2xTy (blue). Time post-activation (0–15 min; m.p.a.) is indicated. ARK1 is initially diffuse within the nucleus, accumulates at discrete foci and spindle-associated structures during activation, and diminishes before exflagellation. DIC images are shown at left. Scale bar, 2.5 μm. **B** U-ExM of ARK1 localisation during microgametogenesis. NHS-ester marks MTOCs (grayscale), α/β-tubulin marks spindle/axonemal microtubules (magenta), AKiT5 marks inner kinetochores (green), and ARK1 is detected by anti-Ty antibodies (red). Scale bar, 1 μm; dimensions are non-expanded. Representative localisation patterns in A and B were reproduced in three independent biological replicates. **C** ARK1 interactome in male-only microgametocytes (*fd*^*1−*^*::ARK1-3xHA*). Scatter plots show relative protein enrichment following ARK1-3xHA immunoprecipitation under native and cross-linked conditions. Cross-linking stabilises additional labile associations, including MCM helicase subunits, centromeric/chromatin-associated proteins and AKiT7. Proteins are coloured by functional group as indicated. **D** Comparison of ARK1-3xHA and AKiT1-3xHA immunoprecipitations from male-only gametocytes. The plot compares ARK1 and AKiT1 pulldowns under native and low cross-linking conditions, identifying INCENP-A as a robust ARK1-enriched interactor. Right: live-cell imaging of INCENP-A-mBFP2-2xTy (blue) with AKiT5 (green) during microgametogenesis. DIC images are shown. Scale bar, 2.5 μm. The INCENP-A localisation pattern in D was reproduced in four independent biological replicates. Source data are provided as a Source Data file.

To follow ARK1 in male gametocytes, we generated parasites expressing ARK1-mBFP2-2xTy from the endogenous locus and confirmed correct tagging by diagnostic PCR and anti-Ty immunoblotting (Figure S3A–C). Live imaging showed that ARK1 is initially diffuse within the nucleoplasm but, upon activation, accumulates in transient foci and spindle-like structures that track subsets of NUF2- and AKiT5-marked kinetochores before diminishing again prior to exflagellation (Fig. 3A). U-ExM placed ARK1 at spindle-pole MTOCs before activation and then along spindle microtubules and kinetochores during mitosis (Fig. 3B). Therefore, despite the unusual organisation of male gametogenesis, ARK1 retains the hallmark localisation pattern of an Aurora-family regulator acting at spindle poles, spindle microtubules and kinetochores.

To explore the proteins associated with ARK1 during male mitosis, in particular regulatory subunits analogous to the Chromosomal Passenger Complex (CPC) of Aurora B kinase (INCENP, SURVIVIN and BOREALIN), we immunoprecipitated endogenously tagged ARK1-3xHA from male-only *fd*^*1-*^ gametocytes to distinguish from contaminating, non-mitotic female interactions, under native conditions and after sequential formaldehyde cross-linking, and compared these datasets along with similar AKiT1-3xHA pulldowns (Fig. 3C, D; Figure S3D–F and Supplementary Data 3). Under native conditions, ARK1 reproducibly co-purified with α-/β-tubulin and a single INCENP paralog, PBANKA_1343200, among the highest-scoring hits. Because full-length sequence relationships among apicomplexan INCENP paralogs remain difficult to resolve and our analyses do not confidently support one-to-one orthology across lineages (discussed later, Fig. 8A–C and Figure. S7), we use INCENP-A/B nomenclature throughout and refer to PBANKA_1343200 as INCENP-A.

Incremental cross-linking recovered a broader and more labile ARK1 neighbourhood that included spindle and kinetochore proteins such as EB1 and SPC24, centromeric/chromatin-associated proteins, MCM helicase subunits, and regulators previously implicated in male spindle function, including CDPK4, NEK1, the CRK5–SOC2 complex (Fig. 3C; Figure. S3E, F). The spindle assembly checkpoint protein homolog AKiT7/MAD1 was identified upon high-cross-linking conditions alone. Direct comparison of ARK1 and AKiT1 pulldowns resolved INCENP-A as the most stable ARK1-specific interactor under native conditions, whereas wider spindle/kinetochore- and chromatin-associated environments were preferentially stabilised by cross-linking (Fig. 3D; left panel). Consistent with this biochemical interaction, live imaging of INCENP-A-mBFP2-2xTy showed a localisation pattern similar to ARK1 during male gametogenesis, with diffuse nuclear signal and enrichment near AKiT5-labelled kinetochores during mitosis (Fig. 3D; right panel).

### ARK1 and INCENP-A are required for bipolar spindle formation and kinetochore segregation during microgametogenesis

**Fig. 4:**
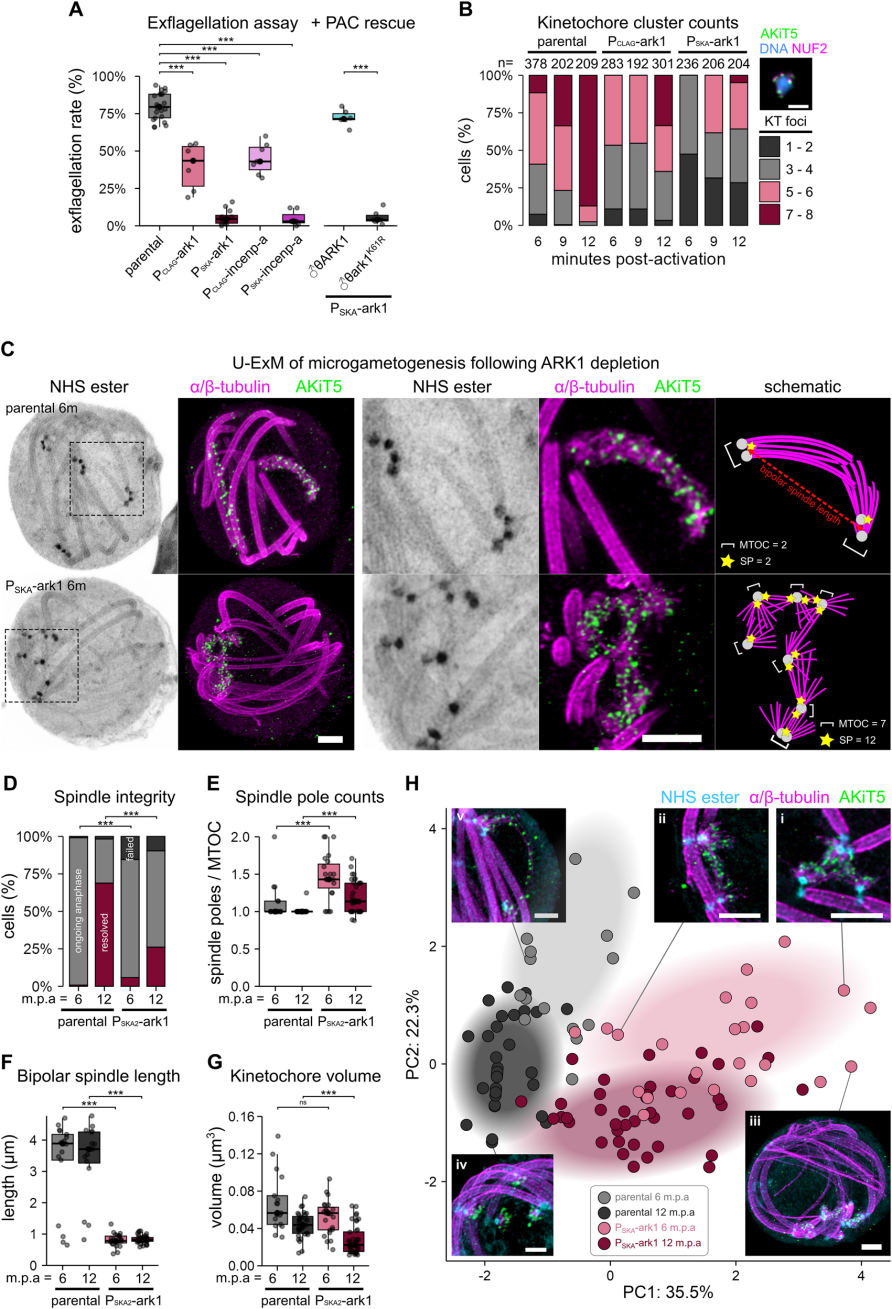
ARK1 is required for bipolar spindle integrity and kinetochore segregation during microgametogenesis. **A** Exflagellation rates of ARK1- and INCENP-A–depletion lines compared with parental controls, and PAC-mediated rescue of P_SKA_-ark1 with full-length ARK1 (♂ARK1) or kinase-dead ark1^K61R^. 6 independent biological replicates; each mouse-derived parasite preparation is the unit of analysis. Boxplots show the median centre line, interquartile-range box and 1.5×IQR whiskers; points are individual biological replicates. **B** Kinetochore (KT) cluster counts per nucleus by live imaging at the indicated minutes post-activation in parental, P_CLAG_-ark1 and P_SKA_-ark1 lines. Stacked bars show the percentage of cells with 1–2, 3–4, 5–6, or 7–8 KT foci; *n* values above bars indicate cells analysed. Scale bar, 5 μm. **C** Representative U-ExM micrographs at 6 min post-activation comparing parental and PSKA-ark1 microgametocytes. NHS-ester marks MTOCs (grey), α/β-tubulin marks microtubules (magenta), and AKiT5 marks kinetochores (green). Scale bars, 1 μm; dimensions are non-expanded. Representative phenotypes in C and H were observed across three independent biological replicates. (**D**) U-ExM classification of spindle morphologies at 6 and 12 min post-activation into resolved, ongoing anaphase, and failed categories. (**E**–**G**) U-ExM quantification of spindle poles per MTOC, bipolar spindle length and kinetochore volume per cell. For (**D**–**G**), *n* = 19 parental 6 m.p.a., 31 parental 12 m.p.a., 22 PSKA-ark1 6 m.p.a., and 40 PSKA-ark1 12 m.p.a. fully reconstructed microgametocytes from three independent biological replicates; each cell is the unit of analysis. Statistical significance was assessed using chi-squared tests for (**D**) and two-sided Wilcoxon rank-sum tests for (**A**, **E**–**G**), with no adjustment for multiple comparisons; exact *P* values are provided in the Source Data file. **H** Principal-component analysis of U-ExM-derived measurements distinguishing parental and P_SKA_-ark1 microgametocytes. Scale bars, 1 μm. Source data are provided as a Source Data file.

Our results indicate that male gametocytes deploy a minimal (2-component) CPC with a stable ARK1–INCENP-A core, together with a broader set of more labile spindle- and kinetochore-associated contacts. Given the conserved roles of the CPC in spindle organisation, kinetochore–microtubule interactions and chromosome partitioning, we assessed the consequences of CPC depletion on spindle integrity and kinetochore organisation during microgametogenesis. Because ARK1 and INCENP-A are essential during asexual proliferation^34,35^, we used promoter-swap strategies to reduce their expression specifically in sexual stages. The native 5′UTRs of *ARK1* and *INCENP-A* were swapped for those of genes that would maintain relative levels during blood stages but are differentially regulated across mosquito stages (Fig. 4A and Figure. S4A–C): *CLAG* (P_CLAG_), known to have lower expression in females relative to males^36^, and *SKA2* (P_SKA_), a kinetochore component detectable in female but not in male cells^13^.

Depletion of ARK1 or INCENP-A severely impaired male gametogenesis, with the strongest effect seen upon P_SKA_-driven downregulation: exflagellation rates dropped to <10% of parental levels by 15 min post-activation (Fig. 4A; Supplementary Data 4). This defect was rescued by PAC-mediated expression of full-length *ARK1*, but not by an *ark1*^*K61R*^ mutant, which carries the homologous substitution to the catalytically inactive human *aurora B*^*K106R*^ ^37,38^, together strongly suggesting ARK1 kinase activity is required for successful microgametogenesis (Fig. 4A and Figure. S4D, E).

To determine whether failed exflagellation reflects defective chromosome segregation, we quantified kinetochore foci using native NUF2-mScI and AKiT5-mNG-3xHA fluorescence. Compared with parental parasites, ARK1-depleted microgametocytes accumulated fewer visible kinetochore clusters over time (Fig. 4B). However, U-ExM showed that these reduced counts largely reflect unresolved aggregates of kinetochores within severely abnormal spindle architectures rather than simple failure to duplicate kinetochores (Fig. 4C). Quantitative analysis of the U-ExM reconstructions identified several specific defects (Supplementary Data 5; Supplementary Videos 1, 2 and 3; Supplementary Data 11). Firstly, we categorised spindle–kinetochore morphologies into segregating (“ongoing anaphase”), resolved (“post-segregation”), and persistent defective (“failed”) classes (Figure S4F). While parental parasites shifted predominantly towards the resolved category by 12 min post-activation, ARK1 depletion led to an enrichment of segregating and persistent spindles at both 6 and 12 min (Fig. 4D). Notably, while overall numbers of MTOCs per cell remained comparable between lines (Figure S4G), ARK1-depleted cells displayed increased numbers of spindle poles relative to MTOC units (Fig. 4E), resulting in multipolar spindles with markedly shortened spindle microtubules (Fig. 4F) and reduced kinetochore volume at later time points (Fig. 4G).

Consistent with broader uncoupling of the division machinery, depletion of ARK1 also led to an over-amplification of basal bodies per microgametocyte, resulting in a marked increase in “free” basal bodies otherwise not connected to a spindle apparatus (Figure S4H, I, J). This phenotype is reminiscent of the MTOC–kinetochore uncoupling observed after disruption of the NDC80 complex in blood-stage parasites^14^.

Principal-component analysis (PCA) identified features strongly distinguishing ARK1-depleted cells from parental controls at both 6 and 12 minutes post-activation (Fig. 4H). The first two components encompass 58% of total variance (PC1 = 36%, PC2 = 22 %). Separation along PC1 primarily captured variation in spindle polarity and structural integrity. Cells with higher PC1 scores showed elevated spindle-pole connections (Fig. 4H; panel i) and a greater proportion of short (Fig. 4H; panel ii) or persistent defective spindles lacking kinetochores (Fig. 4H; panel iii). Lower PC1 scores marked cells with well-segregated kinetochores (Fig. 4H; panel iv), typical of parental lines. PC2 reflected variation in mitotic progression and chromosome segregation, driven by average spindle length and kinetochore volume per cluster. This was particularly evident in parental lines, which showed a shift in PC2 between 6 and 12 minutes post-activation, consistent with ongoing anaphase spindles (Fig. 4H; panel v).

Together, these data show that although male gametogenesis proceeds without obvious repeated metaphase-like alignment, the ARK1–INCENP-A CPC remains essential for bipolar spindle integrity and orderly kinetochore segregation during microgametogenesis mitosis.

### ARK1 and INCENP-A are required for mosquito-stage development and for faithful meiotic chromosome segregation

Erroneous spindle–kinetochore attachments are corrected through antagonism between CPC kinase activity and counteracting PPP1/PP2A phosphatases^6^. In *P. berghei*, however, microgametogenesis appears unusually error-prone, despite the requirement for CPC activity in male gamete formation. We therefore asked whether the apparent reduction of the CPC to an ARK1–INCENP-A core during microgametogenesis contributes to this vulnerability, and whether CPC activity is required for onward transmission through the mosquito.

**Fig. 5:**
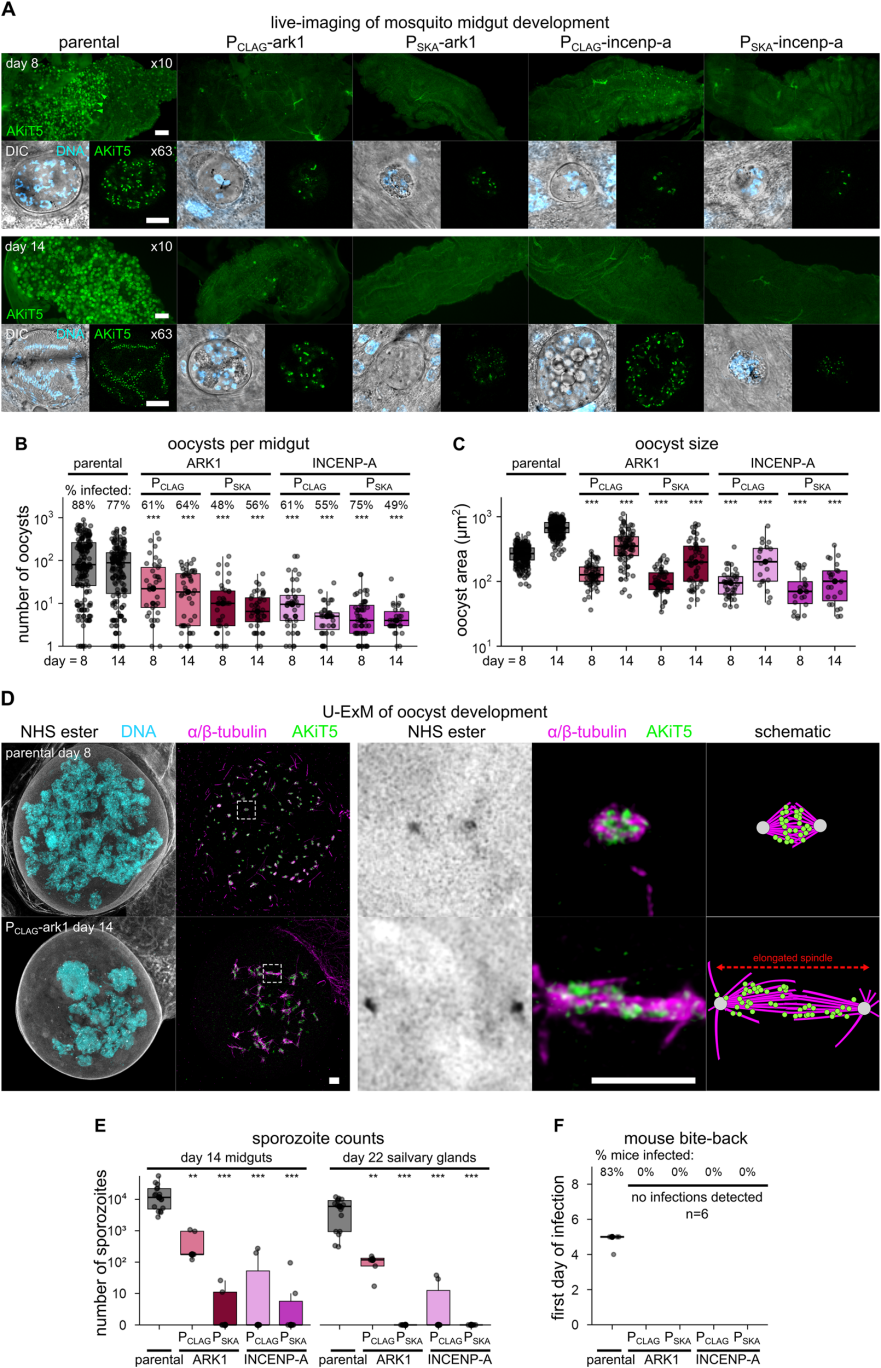
ARK1 and INCENP-A are required for mosquito-stage development and transmission. **A** Live imaging of dissected mosquito midguts at day 8 and day 14 post-blood meal infected with parental parasites or ARK1/INCENP-A depletion lines. AKiT5 native fluorescence marks developing oocysts. Low-magnification views show oocyst burden; high-magnification views show DIC, DNA and AKiT5 in representative oocysts. **B** Oocysts per midgut at day 8 and day 14. Each point represents one midgut and is the unit of analysis; percentages indicate dissected midguts containing ≥1 oocyst. **C** Oocyst cross-sectional area at day 8 and day 14. Each point represents one oocyst and is the unit of analysis. **D** U-ExM of oocyst development in parental and PCLAG-ark1 parasites. NHS-ester marks cell/MTOC material (grey), DNA is cyan, α/β-tubulin is magenta, and AKiT5 is green. Scale bars, 1 μm; dimensions are non-expanded. Representative images in (**A**, **D**) were reproduced across five independent mosquito-feeding experiments. **E** Sporozoite counts from day 14 midguts and day 22 salivary glands. Each point represents one pooled homogenate normalised per organ and is the unit of analysis. **F** Mouse bite-back transmission assay showing the first day of detectable blood-stage parasitaemia. Percentages indicate the fraction of mice infected per group. For (**B**, **C**, **E**), *n* values above plots indicate the number of midguts, oocysts or pooled homogenates analysed from six independent mosquito-feeding experiments. Boxplots show the median centre line, interquartile-range box and 1.5×IQR whiskers; points are individual observations. Statistical significance was assessed relative to parental controls at matched time points using two-sided Wilcoxon rank-sum tests with no adjustment for multiple comparisons; exact *P* values are provided in the Source Data file. Source data are provided as a Source Data file.

Consistent with ARK1 and INCENP-A acting as core CPC components, depletion of either protein markedly reduced oocyst infection of the mosquito midgut (Fig. 5A; ×10; Supplementary Data 6). The few oocysts that do form display abnormal morphology together with aberrant kinetochore organisation (Fig. 5A; ×63). These defects are already evident by day 8 post-blood meal, with significant reductions in both oocyst number and size (Fig. 5B, C). Notably, depletion of ARK1 or INCENP-A under P_CLAG_ regulation, despite causing only a mild reduction in microgametogenesis, has a strong impact on mosquito-stage development. U-ExM further revealed ARK1-depleted oocysts with elongated mitotic spindles and defective kinetochore partitioning (Fig. 5D); however, low oocyst numbers and limited imaging depth (working distance <500 µm for a 40× objective^39^) prevented further quantification of these phenotypes. Consistent with impaired oocyst development, infected mosquitoes produce dramatically fewer sporozoites, with strongly reduced parasite loads in day 14 midguts and day 22 salivary glands (Fig. 5E), and no detectable transmission to mice in bite-back assays (Fig. 5F).

**Fig. 6:**
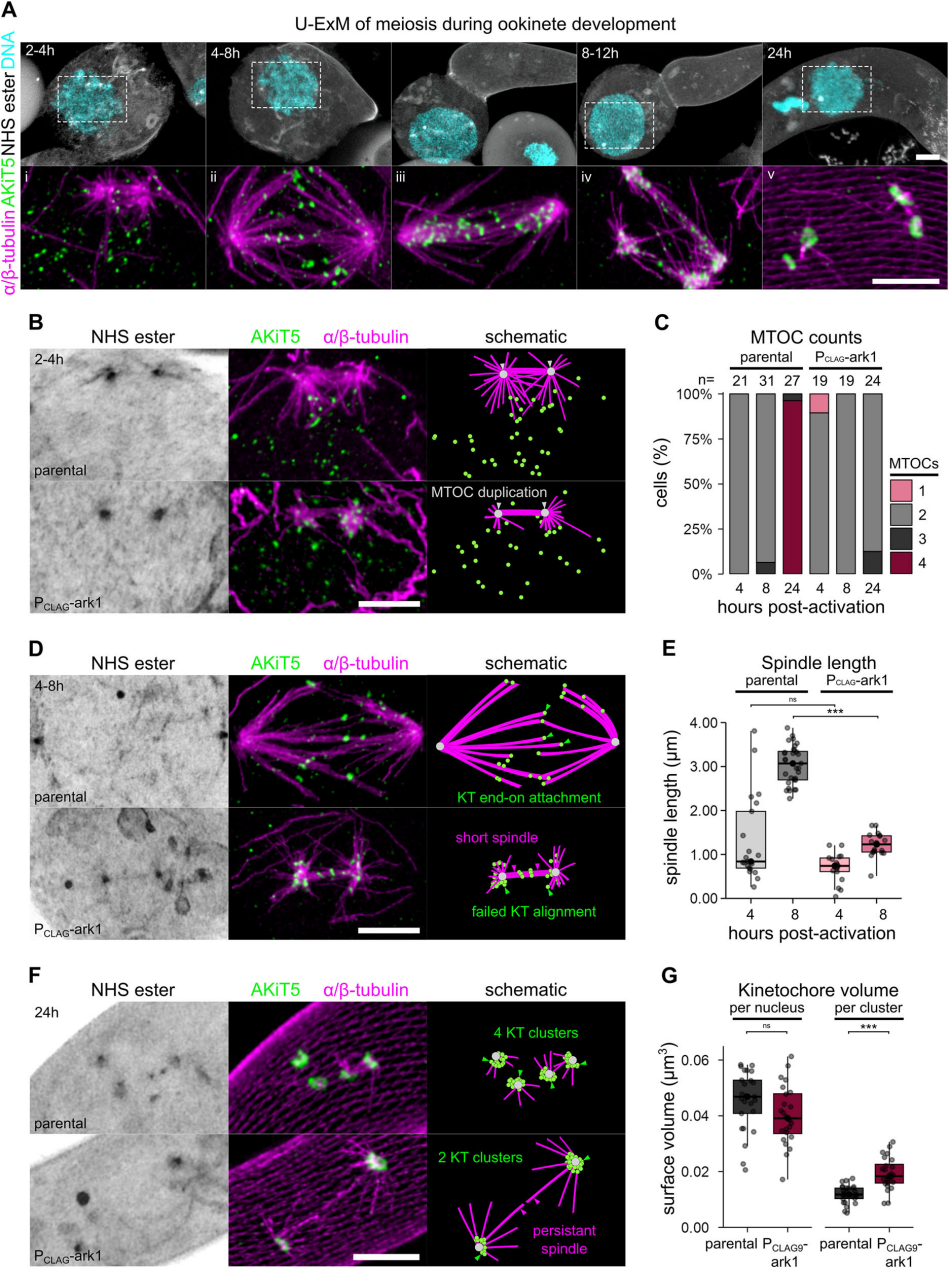
ARK1 is required for meiotic spindle elongation and kinetochore organisation during zygote/ookinete development. **A** U-ExM time course of spindle and kinetochore dynamics during ookinete meiosis. NHS-ester and SYTOX mark MTOCs and DNA (cyan), α/β-tubulin marks microtubules (magenta), and AKiT5-mNG-3xHA marks kinetochores (green; detected with anti-HA). Stages illustrate MTOC duplication, bipolar spindle formation, kinetochore capture/alignment, anaphase-like spindle remodelling, and four KT clusters in mature ookinetes. Boxed regions are magnified below. Scale bar, 1 μm; dimensions are non-expanded. **B** Representative U-ExM images at 2–4 h post-fertilisation showing MTOC duplication in parental and P_CLAG-_ark1 parasites. **C** Quantification of MTOCs per cell in parental and PCLAG-ark1 parasites at 4, 8 and 24 h post-fertilisation. Stacked bars show the percentage of cells containing 1–4 MTOCs; n values above bars indicate fully reconstructed cells analysed. **D** Representative U-ExM phenotypes at 4–8 h post-fertilisation comparing parental and P_CLAG_-ark1 parasites. Parental cells form elongated bipolar spindles with KT end-on attachment, whereas PCLAG-ark1 cells exhibit short spindles and failed KT alignment. **E** Bipolar spindle length at 4 and 8 h post-fertilisation. *n* = 21 parental 4 h, 30 parental 8 h, 19 P_CLAG_-ark1 4 h and 19 P_CLAG_-ark1 8 h fully reconstructed cells from three independent biological replicates; each cell is the unit of analysis. **F** Representative U-ExM examples at 24 h post-fertilisation comparing parental and PCLAG-ark1 parasites. Representative images in (**A**, **B**, **D**, **F**) were observed across three independent biological replicates. **G** Kinetochore volume quantification in parental and PCLAG-ark1 parasites, shown per nucleus and per cluster. The same 24 h fully reconstructed cells quantified in (**C**) were used for this analysis. Boxplots in E and G show the median centre line, interquartile-range box and 1.5×IQR whiskers; points are individual cells. Statistical significance was assessed using two-sided Wilcoxon rank-sum tests with no adjustment for multiple comparisons; exact *P* values are provided in the Source Data file. Source data are provided as a Source Data file.

To determine whether this vulnerability arises prior to colonization of the mosquito-midgut epithelium, we tracked spindle and kinetochore dynamics during meiosis post-fertilisation, in zygotes developing into ookinetes (Fig. 6A and Figure S5A). By 2–4 h post-fertilisation, the meiotic MTOC duplicates and nucleates a prominent intranuclear microtubule array. Between 4 and 8 h, AKiT5-marked kinetochores transition from a dispersed organisation to a captured bipolar spindle-associated configuration, including aligned states consistent with end-on kinetochore–microtubule attachment and biorientation (Fig. 6A; panel ii, Supplementary Video 4). Although AKiT5 puncta were also seen along a few spindle microtubules, the dominant feature of meiosis was the appearance of end-on kinetochore attachment (Supplementary Data 12), a feature not seen during male mitosis. Between 8–12 h post-fertilisation, kinetochores undergo sequential anaphase-like segregations as the spindle remodels into bundled microtubules (Fig. 6A; panels iii-iv), followed by migration of the nucleus along the body of the developing ookinete as it elongates (Figure S5A). Noteworthy is the presence of 3 MTOCs and multipolar spindles in a subset of cells (Figure S5B), indicating completion of the first chromosome segregation is not absolutely required for a second MTOC duplication. By 24 h, mature ookinetes contain four discrete kinetochore clusters (Fig. 6A; panel v), consistent with completion of meiosis and establishment of four haploid genome complements within a single nucleus.

ARK1 and INCENP-A depletion did not prevent overall differentiation into banana-shaped ookinetes (Figure S5C). However, quantification of NUF2-mScI and AKiT5-mNG-3xHA-labelled kinetochores showed a shift from four kinetochore clusters in parental parasites to predominantly two or three clusters in ARK1-depleted cells (Figure S5D). Rescue experiments localised this defect to the zygote/ookinete stage and strongly suggested ARK1 catalytic activity is required for proper meiotic progression: only full-length ARK1, and not ark1^K61R^, restored normal four-cluster segregation (Figure S5D–F).

U-ExM pinpointed the underlying meiotic defect (Fig. 6B–G, Supplementary Data 7, Supplementary Videos 5, 6, 7 and 8). ARK1-depleted zygotes initiate the first round of MTOC duplication (Fig. 6B, C), but fail to establish or maintain an elongated bipolar spindle (Fig. 6D, E). Instead, they form short or disorganised spindles that fail proper kinetochore alignment. Strikingly, this defect leads to a complete meiotic arrest; ARK1-depleted cells do not progress to the subsequent MTOC duplication required to generate four kinetochore clusters (Fig. 6C, F). Notably, total kinetochore volume per nucleus is broadly unchanged, whereas kinetochore volume per cluster is increased (Fig. 6G), indicating kinetochores become consolidated into fewer, larger clusters, a pattern more consistent with a segregation/partitioning defect than failure in DNA replication and centromere/kinetochore establishment.

### An expanded CPC including INCENP-B links ARK1 to attachment-regulatory modules during meiosis

**Fig. 7:**
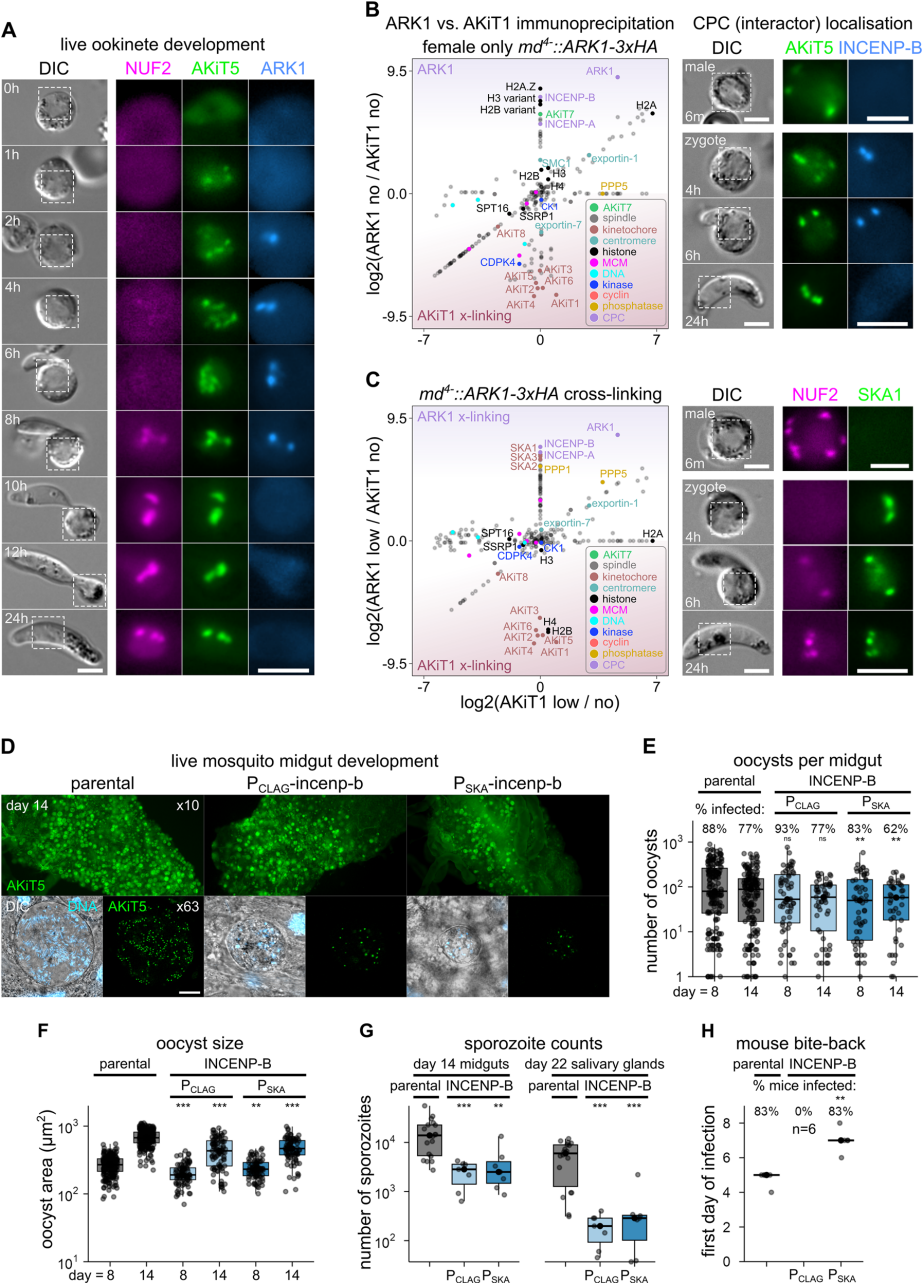
An expanded meiotic CPC engages spindle poles and kinetochores during mosquito-stage transmission. **A** Live widefield imaging of *P. berghei* ookinete development expressing NUF2-mScI (magenta), AKiT5-mNG-3xHA (green) and ARK1-mBFP2-2xTy (blue), with DIC. Time is shown in hours post-fertilisation. Scale bar, 2 μm. **B**, **C** ARK1-3xHA interactomes from female-only md4− zygotes 8 h post-fertilisation, compared with AKiT1 control pulldowns following mild cross-linking. Selected proteins are annotated by functional class. Right: live localisation of (**B**) INCENP-B-mBFP2-2xTy with AKiT5-mNG-3xHA and (**C**) SKA1-mNG-3xHA with NUF2-mScI during male gametogenesis and zygote/ookinete development. Scale bars, 2 μm. Representative localisation patterns in A–C were reproduced in three independent biological replicates. **D** Representative mosquito-midgut images at day 14 post-blood meal from infections with parental parasites or INCENP-B-depletion lines. Widefield ×10 views show AKiT5-marked oocysts; ×63 views show DIC, DNA and AKiT5. Scale bars: ×10, 100 μm; ×63, 20 μm. Representative images were reproduced across six independent mosquito-feeding experiments. **E** Oocysts per midgut at day 8 and day 14. Each point represents one infected midgut and is the unit of analysis; midguts with zero oocysts are not plotted. Percentages indicate dissected midguts containing ≥1 oocyst. **F** Oocyst cross-sectional area. Each point represents one oocyst and is the unit of analysis. **G** Sporozoite counts from day 14 midguts and day 22 salivary glands. Each point represents one pooled homogenate normalised per organ and is the unit of analysis. **H** Bite-back transmission assay showing first detectable blood-stage infection. For (**E**–**H**), exact n values for each genotype, time point and organ are provided in the Source Data file and Supplementary Data 6; data were collected from six independent mosquito-feeding experiments. Boxplots show the median centre line, interquartile-range box and 1.5×IQR whiskers; points are individual observations. Statistical significance was assessed using two-sided Wilcoxon rank-sum tests with no adjustment for multiple comparisons; exact *P* values are provided in the Source Data file. Source data are provided as a Source Data file.

In the zygote, ARK1 is required to establish a functional bipolar meiotic spindle and partition kinetochore clusters evenly, failure of which blocks meiotic progression. The greater architectural dependence of meiosis prompted us to ask whether ARK1 itself is deployed differently during zygote development. Live imaging of ARK1-mBFP2-2xTy showed that most signal is diffuse throughout zygotes during ookinete development (Fig. 7A). In the nucleus, AKiT5 foci are present immediately post-activation of gametogenesis^13^. In contrast, ARK1 transiently accumulates in peri-nuclear pole-like foci during the 2–4 h window of spindle assembly and chromosome segregation. As meiosis proceeds, ARK1 remains enriched at these pole-like structures during the window in which NUF2 kinetochores become clearly detectable (typically between 4–6 h; Figure. S6A^13^). ARK1 foci rapidly reduce to below detectability and diffuse levels by 10 h of development. U-ExM confirmed ARK1 accumulation at spindle poles/MTOCs and additionally revealed discrete ARK1 puncta along bipolar spindle microtubules, many overlapping AKiT5- and NUF2-labelled kinetochores (Figure S6B).

To define the meiotic ARK1 interaction network, we immunoprecipitated ARK1-3xHA from female-only *md*^*4-*^ zygotes at 8 h post-fertilisation (crossed to untagged *fd*^*1-*^ males), and compared it with AKiT1 control pulldowns together with limited cross-linking (Fig. 7B and Supplementary Data 8). In this meiotic context, ARK1 robustly associates not only with INCENP-A but also with a second INCENP paralog, INCENP-B (PBANKA_1353900), together with AKiT7/MAD1 and several chromatin-associated proteins. Limited cross-linking stabilised additional interactions, most notably with the SKA complex and PP1, linking the meiotic CPC to factors that in other systems reinforce end-on attachment and help silence checkpoint signalling.

Live imaging supports stage-specific CPC interactions. INCENP-B is not clearly detectable during male microgametogenesis but accumulates at spindle-pole-like foci during zygote development before disappearing again after meiosis (Fig. 7B; right panel, Figure S6C, D). Similarly, SKA1 accumulates on the meiotic spindle and kinetochores but is not detected in male microgametocytes, consistent with a meiosis-specific role in attachment regulation. AKiT7, by contrast, remains predominantly at the nuclear periphery and shows only a brief kinetochore-associated window during meiosis (Figure. S6E).

Importantly, ARK1 pulldowns from mixed male/female gametocytes (2.34 line; 6 min post-activation) also recovered both INCENP-A and INCENP-B, as well as AKiT7 under native, i.e. non-crosslinking conditions (Figure. S6F and Supplementary Data 8). Similar INCENP-B and AKiT7 associations are not detected in male-only ARK1 interactomes, demonstrating they arise in female/zygote stages and clarifying a limitation of earlier bulk gametocyte interactomes.

Finally, consistent with biochemical and localisation evidence, depletion of INCENP-B has little or no clear effect on male exflagellation (Figure S6G, H), but causes a strong mosquito-stage defect. Oocysts are smaller, sporozoite output reduced, and transmission depends on the stage at which INCENP-B was depleted: the female/meiotic-restricted P_CLAG_-incenp-b line fails to transmit, whereas the P_SKA_-incenp-b line retains transmission but with delayed patency (Fig. 7D–H and Figure. S6G, H).

Together, these data indicate that meiosis deploys a broader CPC assembly built around the ARK1–INCENP core and connected to SKA/PP1- and AKiT7-associated modules that are absent, or only weakly engaged, during male mitosis.

### Comparative analyses support INCENP classes but do not resolve duplication timing

The stage-specific deployment of INCENP-A and INCENP-B suggests extensive rewiring of chromosome-segregation machinery during *P. berghei* sexual development. However, whether this division of labour reflects a broader diversification of the chromosomal passenger complex across apicomplexans or a specific innovation within haemosporidians remains unclear. The presence of two INCENPs has been reported in *Toxoplasma gondii*, INCENP1 and INCENP2, with proper levels of INCENP2 required for TgARK1 function^40^. We therefore asked two questions: whether duplicated apicomplexan INCENPs resolve into recurring cross-lineage similarity classes, and whether those classes are better explained by an ancient duplication or by parallel lineage-specific duplication events.

In an attempt to sensitively identify INCENP proteins, we employed an iterative Hidden Markov Model (HMM) profiling strategy. Briefly, HMMs constructed from INCENP homologs^18^ were used as search queries across an in-house database of proteomes generated from alveolate protein sequences (Supplementary Data 9) and significant hits were concatenated iteratively. Full-length maximum-likelihood phylogeny of INCENP sequences recovered sensible lineage-level groupings, including haemosporidian, coccidian, piroplasmid, nephromycid and cryptosporidiid clades, but provided weak support for the deeper backbone (Figure S7). Outside the conserved Aurora-binding IN-box, domain architecture was highly heterogeneous, with extensive variation in coiled-coil and low-complexity regions, making deeper full-length alignments difficult to interpret.

**Fig. 8:**
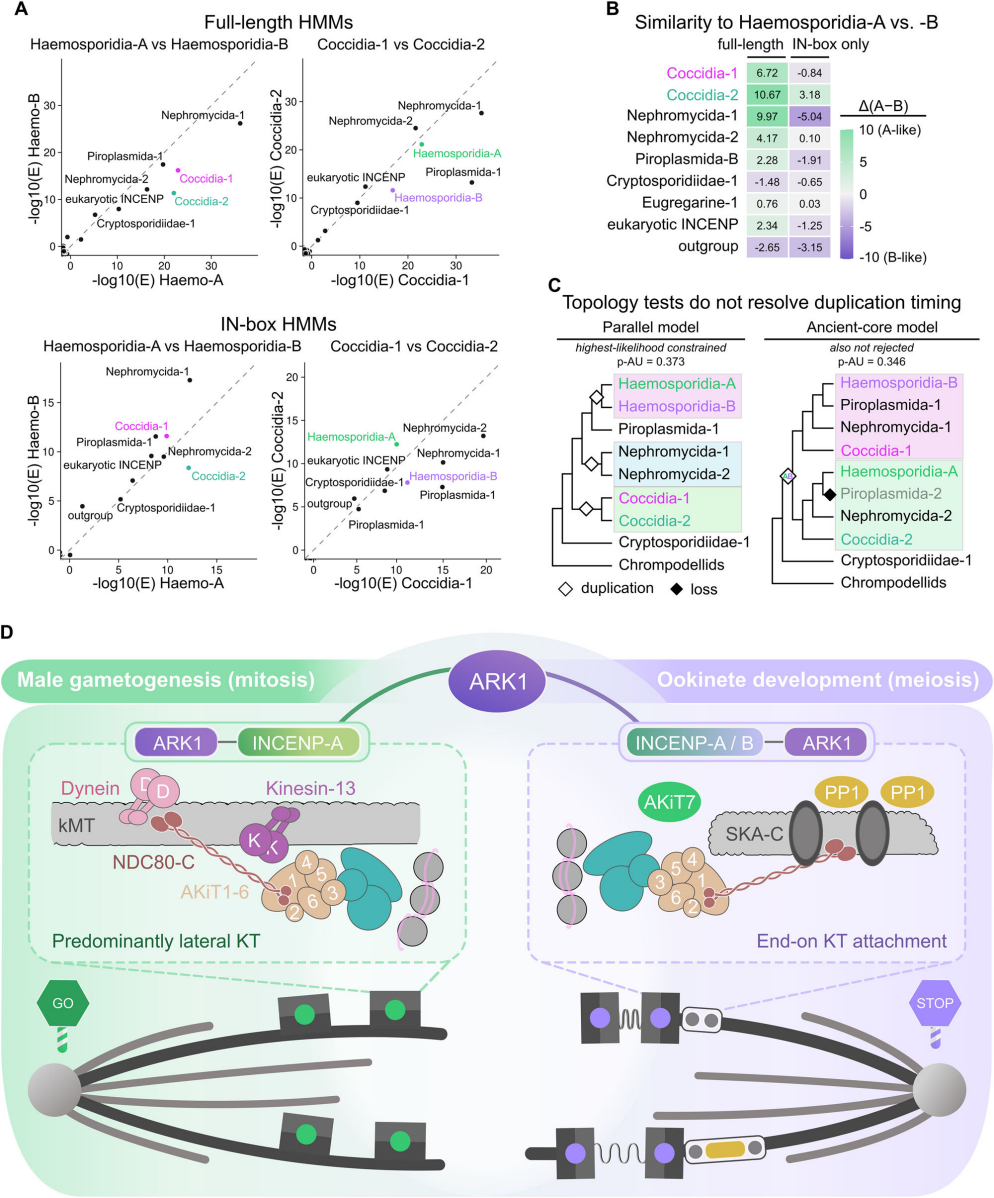
Domain-restricted INCENP analyses reveal cross-lineage class correspondence but unresolved duplication timing. **A** HHsearch profile–profile comparisons between INCENP HMMs built from full-length proteins or IN-box-only regions. Left plots compare similarity to Haemosporidia-A and Haemosporidia-B reference models; right plots compare similarity to Coccidia-1 and Coccidia-2 reference models. Axes show −log10(E) values; dashed diagonals indicate equal similarity to the two reference HMMs. **B** Heatmap summarising Δ(A − B) values relative to Haemosporidia-A and Haemosporidia-B HMMs for full-length and IN-box-only analyses. Δ(*A* − *B*) = −log_10_(*E*_*A*_) − [−log_10_(*E*_*B*_)]; positive values indicate A-like similarity and negative values indicate B-like similarity. **C** Schematic constrained topologies tested by approximately unbiased (AU) analysis on the conserved alignment. The parallel-duplication model was the best-fitting constrained model, but neither the parallel nor the ancient-core duplication model was rejected. Open and filled diamonds indicate inferred duplication and loss events. **D** Working model for stage-specific ARK1-associated chromosome-segregation states during *P. berghei* sexual development. Male gametogenesis is associated with a minimal ARK1–INCENP-A CPC and predominantly lateral kinetochore–microtubule interactions at anaphase. Ookinete development is associated with an expanded meiotic CPC including INCENP-A/B, AKiT7 and SKA-C/PP1-associated modules, consistent with end-on kinetochore attachment and tighter coupling of attachment state to meiotic progression. Springs denote tension-bearing attachments, and the yellow module denotes attachment- or tension-sensitive states. In this model, male gametogenesis follows a more permissive segregation programme (‘GO’), whereas meiosis engages attachment-state control, such that failure to satisfy it blocks meiotic progression (‘STOP’). The stoichiometry of ARK1 association with INCENP-A and INCENP-B during meiosis remains unresolved.

To investigate the impact of architectural noise, we compared lineage-specific HMMs built from either full-length INCENP or the IN-box alone (Fig. 8A, B). Full-length profile space was strongly shaped by non-core architecture and did not cleanly separate all paralogs. By contrast, IN-box-restricted comparisons recovered more interpretable A-like and B-like similarity classes across lineages: for example, Coccidia-1 shifted towards the B-like side whereas Coccidia-2 remained A-like, and Nephromycida-1 and Piroplasmida-B also tended towards B-like similarity, while Nephromycida-2 remained near neutral. Because the conserved IN-box is short, however, the resolving power of this analysis remains limited.

We therefore tested constrained topologies on a longer conserved alignment. Approximately unbiased tests did not reject either a parallel-duplication model or an ancient-core duplication model (Fig. 8C). Although the parallel model provided the marginally best fit among the constrained scenarios tested, the ancient-duplication alternative remained plausible.

Taken together, our data support interpreting INCENP-A-like and INCENP-B-like as cross-lineage similarity classes rather than confident one-to-one orthology assignments across apicomplexans. Therefore, the analyses do not decisively resolve whether these classes arose from a single ancestral duplication or from parallel duplications in different apicomplexan lineages.

## Discussion

*Plasmodium berghei* ARK1 is a kinase that we show is responsible for the precise orchestration of chromosome segregation in both mitotic and meiotic contexts. It localises to spindle poles, spindle microtubules and kinetochore-associated sites, and our data support a model in which ARK1 forms the catalytic core of a divergent chromosomal passenger complex (CPC) that is deployed differently across developmental stages. Our key biological message is not simply that *Plasmodium* species have a CPC different from that of animals and fungi, but, more broadly, our data argue that *Plasmodium* deploys different ARK1-centred chromosome-segregation regimes at different lifecycle stages, with important consequences for how faithfully chromosomes are transmitted (Fig. 8D).

The identification of kinetochore components and metaphase-like biorientation in *Plasmodium berghei*, as well as functional characterization in *Toxoplasma gondii*^12,13,41^, together with the high retention of centromere-bearing loci during *Plasmodium* blood-stage proliferation^31^, has challenged the traditional view that apicomplexan parasites possess a reductive, idiosyncratic machinery incapable of high-fidelity chromosome segregation^15–17^. Male gametogenesis, however, appears to follow a strikingly different chromosome-segregation regime to asexual blood-stage division. Although the speed and asynchrony of male division make interpretation difficult, our conclusions are grounded in quantitative U-ExM analyses of fully reconstructed three-dimensional cells spanning non-activated and closely spaced post-activation time points. Across our dataset, we did not observe a convincing example of kinetochore congression to an equatorial plate or metaphase-like kinetochore alignment. Instead, spindle assembly proceeds through progressive MTOC partitioning, with kinetochores redistributed along spindle microtubules. The first mitotic spindle also contains substantially more spindle and kinetochore material than later spindles, supporting a progressive partitioning regime rather than simple sequential diploid duplication–elongation–split cycles. This interpretation is consistent with early ultrastructural descriptions of a progressive reduction in spindle microtubule number across successive male divisions^21^, and raises the possibility that the initial spindle engages more than a diploid complement of chromosomes. One possible explanation is that centromeres and pericentromeric regions replicate before the entire length of chromosome arms during microgametogenesis, consistent with the reported localisation of DNA replication factors to the nuclear periphery in *P. falciparum*^42^, and this could help explain why changes implied by spindle and kinetochore content are not readily captured by bulk DNA-content flow cytometry^43–45^. This interpretation is further indirectly supported by recent work in *Toxoplasma gondii*, where engagement of DNA replication machinery at centromeric regions at the onset of S-phase suggested that centromeres are among the first genomic regions to replicate^46^, and by evidence of centromere-first replication in more widely distributed eukaryotes^47^.

Along with architectural changes, our inheritance assays also indicate that chromosome transmission during sexual development is considerably less faithful than during blood-stage proliferation. Centromere-containing PACs are retained at higher rates during blood-stage growth, yet paternal transmission through fertilisation and ookinete development is markedly less robust. Notably, even the endogenous paternally inherited *SIL6* reporter is lost in approximately 10% of mature ookinetes. This extends previous evidence that male gametogenesis can proceed despite failed or incomplete genome replication^48^. Accordingly, paternal contribution of *SIL6* is consistent with incomplete paternal chromosome transmission, although it does not by itself distinguish whole-genome loss from partial genome loss. Recent work reported a comparable parent-of-origin asymmetry in post-fertilisation homing efficiency at the endogenous *myoA* locus: homing was ~97% efficient when the guide RNA was contributed by the female gamete, but only ~89% efficient when it was contributed by the male gamete^49^. Together with the absence of metaphase kinetochore architecture, these observations support the conclusion that male development operates under a more permissive chromosome-segregation regime than blood-stage proliferation.

One explanation for a permissive male division programme is rewiring of chromosome-segregation machinery. By rewiring, we do not mean loss of the underlying checkpoint logic or attachment surveillance, but reconfiguration of ancestral machinery in *P. berghei*. Apicomplexans do possess recognisable kinetochores, built hierarchically from conserved inner CCAN and outer KMN components^12,13^. Nevertheless, *Plasmodium* kinetochores remain exceptionally streamlined relative to those of animals and fungi, with fewer than ~15 identified components across the CCAN and KMN sets, and lacking most canonical SAC proteins, except AKiT7/MAD1, as well as major SAC-associated kinases such as MPS1 and PLK. The CPC is similarly reduced. Consistent with the apparent loss of SURVIVIN and BOREALIN from *Plasmodium* genomes^11,18^, our data indicate that ARK1 does not assemble into a canonical four-subunit passenger complex. During microgametogenesis, this reduction is taken further still. Compared to asexual blood-stage divisions, male gametocytes do not detectably engage the SKA complex^21^, a factor that in animal cells is important for establishing and strengthening robust end-on kinetochore–microtubule attachments^50–52^. Similarly, we show here that males deploy a reduced CPC built around a stable ARK1–INCENP-A core, with no clear engagement of INCENP-B. A reduced male kinetochore configuration is well suited to a division programme that prioritises speed and gamete output over maximal attachment correction. Recently, an atypical Arp2/3 complex has been shown to be required to maintain faithful DNA segregation during microgametogenesis^53^, suggesting that chromosome-segregation surveillance may be mediated by evolutionarily different machinery, consistent with checkpoint-like logic implemented through non-canonical components. ARK1 depletion during microgametogenesis does not simply perturb a checkpoint-like delay, but causes short or multipolar spindles and produces over-amplified, “free” outer MTOCs that are uncoupled from intranuclear spindles, indicating a failure to coordinate spindle, MTOC and kinetochore organisation. The recovery of EB1 and SPC24 as ARK1 labile interactors in microgametocytes, together with the phenotypic parallel of ARK1-depletion to NDC80-complex^14^ and CENP-C/SEA1^54^ disruption in *P. falciparum* blood-stages, argues that ARK1 may act at the interface between kinetochore function and spindle/MTOC architecture during male division.

Following fertilisation, however, meiosis reveals phases of chromosome segregation not seen during microgametogenesis. Kinetochores align along the spindle midplane during meiosis, bipolar spindle assembly occurs within a defined temporal window, kinetochores form clear end-on attachments to the spindle prior to anaphase, and further MTOC duplication is delayed when chromosome-segregation fails. Together, these features are consistent with a more stringent surveillance regime that safeguards transmission fidelity.

In parallel, the CPC expands during meiosis. ARK1 associates not only with INCENP-A but also with INCENP-B, AKiT7/MAD1 and chromatin-associated proteins, and crosslinking further brings the complex into proximity with the SKA complex and PP1, factors that reinforce end-on kinetochore attachments and promote SAC silencing in eukaryotic cells^55,56^. Our live imaging supports biochemical identifications: INCENP-B accumulates during zygote development but is not clearly detectable during male mitosis. Similarly, SKA-C is restricted to meiotic spindles and kinetochores during ookinete development^21^. ARK1 also localises closer to the outer than to the inner kinetochore during meiosis, raising the possibility that it directly promotes end-on kinetochore attachment. In animal cells, PP1 antagonises Aurora B–mediated phosphorylation of the SKA complex, stabilising kinetochore–microtubule attachments and permitting anaphase onset under proper tension conditions^7^. It is tempting to speculate that a similar ARK1–PP1 relationship may operate across *Plasmodium* centromeres. Likewise, the recovery of AKiT7/MAD1 in the meiotic context suggests that ARK1 may interface with checkpoint signalling upstream of tension satisfaction. Although our data do not demonstrate a canonical spindle assembly checkpoint in *Plasmodium*, they strongly support the view that meiosis deploys a more attachment-sensitive and fidelity-oriented segregation programme than male gametogenesis. INCENP-B may be particularly important for ARK1-dependent chromosome biorientation and the transmission-critical transition from zygote to ookinete. Recently, INCENP-B has been shown to form part of the *P. falciparum* CPC during intra-erythrocytic divisions^57^. Although INCENP function was not dissected, it supports a model whereby developmental stages that resolve kinetochore metaphase-alignment specifically include INCENP-B within the CPC. The recovery of H2A.Z, H3v and H2B further hints that ARK1 may also have a chromatin-facing role during meiosis, potentially analogous to Aurora-driven chromatin remodelling as described in opisthokonts^58–61^.

A notable strength of the present study is that both chromosome-segregation fidelity and CPC assemblies could be resolved in a sex-specific manner. By combining chromosome inheritance assays and immunoprecipitations with the marker-free single-sex *fd*^*1-*^ and *md*^*4-*^ *P. berghei* lines recently developed by Sayers et al. (2024), which enable sex-resolved phenotyping and downstream biochemical analyses^30^, we avoided the confounding influence of mixed gametocyte populations and gained stage-specific resolution of ARK1 interaction partners during male mitosis and zygote meiosis. Previous inheritance assays were inherently unable to isolate male segregation accuracy, because the reporter locus was already present in the female gamete prior to fertilisation^31^. The same sex-resolved strategy provided stage-specific resolution of ARK1 interaction partners during microgametogenesis versus meiosis, helping to explain why previous studies, including our own, captured composite associations such as SKA-C^13,57^. It also clarifies the stage specificity of AKiT7/MAD1, SKA and INCENP-B, and is consistent with recent work showing that AKiT7/MAD1 has comparatively weak kinetochore association in activated gametocytes^53^. Our sex-specific interactomes also complement recent work identifying both INCENP paralogs in bulk gametocyte populations and providing transcriptomic evidence for a broader ARK1-associated network^57^. Whereas bulk and transcript-level approaches report relative mRNA abundance and global expression dynamics, they do not by themselves resolve CPC composition, interaction stability or division-mode-specific deployment. By combining stage-resolved interactomes with targeted perturbations and high-resolution localisation analyses across two distinct division programmes, our study helps clarify that CPC modularity is developmentally regulated rather than constitutive.

CPC modularity shown here also helps frame the evolutionary meaning of Aurora/CPC divergence in apicomplexans. *Plasmodium* ARKs are Aurora kinases, a family with broadly conserved roles in spindle assembly, kinetochore function and cytokinesis^62^. Aurora systems have been extensively rewired across eukaryotes^63^. In animals and plants, paralogous Aurora kinases often overlap in spindle, CPC and meiotic functions^64^, whereas budding yeast condenses all Aurora-dependent processes into a single kinase, Ipl1^65^. Conversely, the euglenozoan *Trypanosoma brucei* encodes three Aurora kinases and assembles a pentameric CPC comprising AUK1/Aurora B, INCENP/CPC1, CPC2 and the kinesins KIN-A and KIN-B, which promotes accurate kinetochore–microtubule attachments and timely anaphase onset^29,66–68^. In this context, *Plasmodium* fits a broader pattern of Aurora-system plasticity across eukaryotes. ARK1 combines AURKA-like spindle functions, AURKB-like passenger behaviour and AURKC-like meiotic roles.

Genuine novelty in apicomplexan chromosome biology appears in more recent duplications within core kinetochore and CPC systems, as already described for CENP-C/AKiT9/10/SEA1^13,69^, and which we now show for INCENP. Our comparative analyses argue against strong claims about the timing of INCENP duplication. Full-length INCENP phylogenies recover sensible lineage-level clades, as previously noted^18,57^, but provide weak support for deeper relationships. By contrast, IN-box-restricted HMM comparisons reveal clearer cross-lineage A-like and B-like similarity classes, most clearly separating the two coccidian paralogs. Even so, these domain-restricted similarities do not decisively distinguish between parallel-duplication and ancient-core duplication scenarios, and AU tests on the longer conserved alignment do not reject either model. We therefore interpret the current data as supporting cross-lineage A-like and B-like paralog classes, but not confident one-to-one orthology assignments between paralogs from different apicomplexan groups. If an ancient-core duplication were eventually supported, it would be broadly compatible with Aurora kinase expansion in apicomplexans^18^. More broadly, comparative genomics provides a starting point for evolutionary inference, but distinguishing shared ancestry, lineage-specific divergence and functional repurposing ultimately requires integration with cell-biological and biochemical data in widely distributed apicomplexans.

Developmental tuning of chromosome-segregation fidelity may itself help explain why *Plasmodium* kinetochores remain extremely divergent relative to those in animals and fungi. If error correction is weaker during some stages, centromere-biased mis-attachments may persist more readily, potentially relaxing purifying selection on kinetochore interfaces and accelerating turnover of centromere-associated components. From a therapeutic perspective, these stage-specific CPC states may represent exploitable vulnerabilities. The segregation machinery that safeguards parasite transmission in mosquito stages appears indispensable, albeit less buffered during male gametogenesis. Targeting CPC function genetically or pharmacologically could therefore selectively disrupt parasite proliferation and block transmission, while limiting evolutionary escape routes. Consistent with the *Plasmodium* CPC as an attractive drug target, a recent study has chemically validated that the human Aurora B inhibitor hesperadin blocks *P. falciparum* ARK1 with nanomolar potency, providing proof of principle that pharmacological inhibition of CPC function can deliver multistage antimalarial activity^70^.

Taken together, our findings show that *Plasmodium* employs a modular CPC to balance propagation and chromosome-segregation fidelity across distinct developmental divisions. This modularity appears to reflect a solution to stage-specific segregation demands, highlighting a potential vulnerability during parasite transmission.

## Methods

### Ethics statement

All animal experiments were approved by the competent cantonal veterinary authorities under licence numbers GE283C (Canton of Geneva) and BE118/22 (Canton of Bern). For the Geneva licence, applications were evaluated by the Commission Cantonale pour les Expériences sur les Animaux and authorised by the Service de la consommation et des affaires vétérinaires. Experiments were performed in accordance with Swiss Federal Food Safety and Veterinary Office guidelines and the Swiss Animal Welfare Act (Tierschutzgesetz, TSchG). Female mice were used for parasite propagation, mosquito infection and transmission assays. Sex was not analysed as an experimental variable because mice served as hosts for parasite development and mosquito feeding rather than as the biological subject of the chromosome-segregation phenotypes measured.

### Parasites, mice and mosquito husbandry

The *Plasmodium berghei* ANKA clone 2.34 and all derived transgenic lines were maintained in 6- to 8-week-old female CD1 or BALB/c mice obtained from Charles River Laboratories. Mice were housed under specific-pathogen-free conditions in individually ventilated cages at 21 ± 2 °C, with humidity monitored according to institutional SPF facility standards, under a 12 h light/dark cycle, with autoclaved chow and water provided ad libitum. Parasitaemia was monitored on methanol-fixed, Giemsa-stained thin blood smears.

*Anopheles stephensi* mosquitoes were maintained at 28 °C, 80% relative humidity and a 12 h light/dark cycle. Adults were fed 8% fructose supplemented with p-aminobenzoic acid (pABA; 0.2 g/L), and females were blood-fed once weekly for egg production. Larvae were reared in water and fed Liquifry/fish food. After infectious blood meals, mosquitoes were maintained at 20 °C.

### Plasmid construction and generation of transgenic parasite lines

All oligonucleotides are listed in Table S1.

#### Endogenous C-terminal tagging

For endogenous C-terminal tagging, constructs were derived from pCPY-0-based vectors previously used in *P. berghei*^13^. Briefly, ~500 bp from the 3′ end of the coding sequence and ~500 bp from the immediately downstream 3′ untranslated region (UTR) of each target gene were cloned on either side of a fluorescent/epitope tag cassette containing mBFP2-2xTy. Constructs were linearised within the homology region prior to transfection. This strategy was used to generate ARK1-mBFP2-2xTy, INCENP-A-mBFP2-2xTy, INCENP-B-mBFP2-2xTy and AKiT7-mBFP2-2xTy parasites. Correct integration was confirmed by diagnostic PCR and, where appropriate, by anti-Ty immunoblotting.

#### Promoter-swap depletion lines

To deplete essential genes specifically in mosquito stages, promoter-swap constructs were generated from pNP-based vectors in which the endogenous 5′ regulatory region was replaced with the 5′ UTR of either CLAG (P_CLAG_) or SKA2 (P_SKA_), upstream of an N-terminal 2xTy tag. For each construct, ~500 bp of the target ORF and ~500 bp of the endogenous upstream targeting region were cloned into the vector, and the plasmid linearised before transfection. This strategy was used to generate P_CLAG_-ark1, P_SKA_-ark1, P_CLAG_-incenp-a, PS_KA_-incenp-a, P_CLAG_-incenp-b and P_SKA_-incenp-b lines.

#### Reporter loci and *Plasmodium* artificial chromosomes

Chromosome inheritance was monitored using both an endogenous chromosomal reporter and *Plasmodium* artificial chromosomes (PACs).

PAC constructs were derived from pBAT1- and PCEN-based backbones^31,71^. Telomeric repeats and centromere 5 (CEN5) were combined with the same dual-reporter logic used in the SIL6 reporter. PAC-A contained telomeric repeats, CEN5, HSP70-mBFP2 and P28-mScI. PAC-AΔcen lacked the centromeric region and served as an acentromeric control. PAC-B reoriented the reporter cassettes relative to the centromere and replaced the PPPK 3′UTR downstream of mScI with the endogenous P28 3′UTR. PAC-C contained an extended genomic context including an additional STAB sequence upstream of the HSP70 promoter.

For the endogenous reporter, a similar dual-fluorescence cassette containing HSP70-driven mBFP2 and P28-driven mScarlet-I was ordered from GenScript and cloned into pBAT1-SIL6, which integrates at the *SIL6* locus. This generated a single-copy endogenous chromosomal reporter used as a reference for paternal inheritance.

For rescue experiments, PAC-A derivatives were generated in which 2xMyc-tagged ARK1 or the mutant ark1^K61R^ was expressed from male-specific regulatory sequences derived from PBANKA_1431400. Where indicated, additional PAC variants carrying rescue cassettes active in the female/zygote context were used for meiosis-stage complementation.

### *Plasmodium berghei* maintenance and transfection

*P. berghei* ANKA strain-derived clone 2.34^72^, together with derived transgenic lines, was grown and maintained in CD1 outbred and BALB/c mice. 6- to 8-week-old mice were obtained from Charles River Laboratories, and females were used for all experiments. Mice were specific pathogen-free (including *Mycoplasma pulmonis*) and subjected to regular pathogen monitoring by sentinel screening. Mice were housed in individually ventilated cages furnished with a cardboard mouse house and Nestlet, maintained at 21 ± 2°C under a 12 h light/dark cycle and given commercially prepared autoclaved dry rodent diet and water ad libitum. The parasitaemia of infected animals was determined by microscopy of methanol-fixed and Giemsa-stained thin blood smears. For gametocyte production, parasites were grown in mice that had been phenylhydrazine-treated three days before infection. Exflagellation was induced in exflagellation medium (RPMI 1640 containing 25 mM HEPES, 4 mM sodium bicarbonate, 5% fetal calf serum (FCS), 100 μM xanthurenic acid, pH 7.8). For gametocyte purification, parasites were harvested in suspended animation medium (SA; RPMI 1640 containing 25 mM HEPES, 5% FCS, 4 mM sodium bicarbonate, pH 7.20) and separated from uninfected erythrocytes on a Histodenz/Nycodenz cushion made from 48% of a Histodenz/Nycodenz stock (27.6% [w/v] Histodenz/Nycodenz [Sigma/ Alere Technologies] in 5.0 mM TrisHCl, 3.0 mM KCl, 0.3 mM EDTA, pH 7.20) and 52% SA, final pH 7.2. Gametocytes were harvested from the interface.

### Parasite transfection and selection

Schizonts for transfection were purified from overnight in vitro cultures on a 55% Histodenz cushion. Purified schizonts were washed, resuspended in 25 μL Amaxa Basic Parasite Nucleofector solution, mixed with 10 μg linearised DNA in 10 μL H₂O, and electroporated using programme FI-115 on a 4D-Nucleofector (Lonza). Transfected parasites were immediately mixed with 200 μL fresh erythrocytes and injected intraperitoneally into recipient mice. Selection was initiated 24 h later with pyrimethamine (0.07 mg/mL) in drinking water. Correct genomic integration was confirmed by diagnostic PCR.

### Blood-stage propagation, gametocyte production and purification

For gametocyte production, mice were treated with phenylhydrazine hydrochloride three days before infection. Parasites were harvested in suspended animation medium (SA; RPMI 1640 containing 25 mM HEPES, 5% fetal calf serum, 4 mM sodium bicarbonate, pH 7.2) and purified on a 48% Histodenz/Nycodenz cushion prepared in SA. Gametocytes were collected from the interface and washed before downstream applications.

Microgametogenesis was induced in exflagellation/ookinete medium consisting of RPMI 1640 supplemented with 25 mM HEPES, 4 mM sodium bicarbonate, 5% fetal calf serum and 100 μM xanthurenic acid, pH 7.6–7.8.

### Exflagellation assay and ookinete differentiation

Exflagellation was quantified by adding 10–20 μL infected blood to 100 μL activation medium and counting exflagellation centres at 12–15 min post-activation by phase-contrast microscopy using a 60× objective. For in vitro fertilisation and ookinete development, activated sexual stages were incubated at 20 °C, and mature cultures were scored after 24–48 h as indicated for each experiment. For morphology scoring, zygotes/ookinetes were classified as round, retort or mature ookinete based on cell shape in DIC images.

### Mosquito infections, oocyst analyses, sporozoite counts and bite-back

For mosquito-transmission experiments, gametocyte-rich infections were generated by passaging *P. berghei*-infected erythrocytes through phenylhydrazine-treated mice. Briefly, mice were injected intraperitoneally with phenylhydrazine hydrochloride (6 mg/mL in PBS), and when donor infections reached 2–5% parasitaemia, mice were euthanised by CO₂ and parasitised blood was collected by heart puncture and intravenously passaged into pre-treated mice. By 3 days post-injection, parasitaemia typically exceeded 10%, and gametocytaemia exceeded 1%, at which point mice were anaesthetised with ketamine:xylazine and placed in cages containing approximately 100–150 female *Anopheles stephensi* mosquitoes for blood feeding. At the indicated time points post-blood meal, mosquitoes were aspirated using a small vacuum device, anaesthetised with chloroform, and maintained on ice in Petri dishes during dissection. Midguts were dissected at days 8 and 14 post-blood meal, mounted on glass slides in PBS containing 1 μg/mL Hoechst 33342, and imaged on a Leica DM5500 widefield fluorescence microscope using a 10× objective to quantify oocyst numbers and on a Leica TCS SP8 laser-scanning confocal microscope using a 100× objective to assess oocyst morphology. Oocyst numbers were quantified in Fiji using the ‘Find Maxima’ function, followed by manual correction where necessary. Oocyst size was measured manually from high-magnification images as cross-sectional area using the ellipse and ‘Measure’ tools. For sporozoite quantification, day-14 midguts were dissected and homogenised to release oocyst sporozoites, and day-22 salivary glands were dissected and homogenised to release salivary gland sporozoites. Sporozoites were counted in a Neubauer chamber from 10 μL of homogenate, and values were normalised per organ or per mosquito as indicated. For bite-back assays, infected mosquitoes were allowed to feed on naïve mice and blood-stage patency was monitored by blood smear, with the first day of detectable parasitaemia recorded.

### Immunoblotting

Actively dividing cells were washed in PBS and lysed in Laemmli buffer at 95 °C for 5 min. Lysates equivalent to ~5 × 10⁶ cells were resolved on 4–20% polyacrylamide gels and transferred to 0.45 μm nitrocellulose overnight at 1.6 V cm⁻¹. Membranes were blocked in 5% milk in TBS-T, incubated with primary antibody in 1% milk/TBS-T, washed, incubated with HRP-conjugated secondary antibody, and developed by chemiluminescence. Antibodies and dilutions are listed in Table S2.

### Live-cell fluorescence microscopy

Native fluorescence imaging of blood and sexual stages was performed on an inverted Zeiss Axio Observer Z1 microscope equipped with an Axiocam 506 mono camera and a Plan Apochromat 63×/1.4 oil DIC objective. For male gametogenesis, purified gametocytes were activated immediately before imaging and samples were acquired at the indicated minutes post-activation. For zygote/ookinete development, cultures were imaged at the indicated hours post-fertilisation.

Mosquito-midgut and salivary glands were imaged either on a Leica DM5500 widefield microscope or on a Leica TCS SP8 confocal microscope.

### Ultrastructure expansion microscopy (U-ExM)

U-ExM was adapted for *P. berghei* sexual stages from established expansion microscopy protocols^73,74^, as follows. Cells were sedimented onto polyD-lysine-coated coverslips and fixed for 6 min in 2% methanol-free formaldehyde in PBS, followed by 1 min in methanol at −20 °C and three PBS washes containing 100 mM glycine. Coverslips were then incubated overnight at 37 °C in anchoring solution containing 1.4% formaldehyde and 2% acrylamide in PBS.

Gelation was performed in monomer solution containing 23% (w/v) sodium acrylate, 10% acrylamide and 0.1% bisacrylamide with 10% APS and 10% TEMED. Gels were denatured for 1.5 h at 95 °C in denaturation buffer (200 mM SDS, 200 mM NaCl, 50 mM Tris, pH 9.0) and expanded by repeated incubations in ddH_2_O. After the first expansion round, gels were cryopreserved in 50% glycerol if required and stained later.

For immunostaining, gels were blocked in 3% BSA/PBS for 30 min at 37 °C, incubated with primary antibodies in 2% BSA/PBS for 3 h at 37 °C, washed, and incubated with secondary antibodies for 2.5 h at 37 °C. NHS-ester and SYTOX were added after immunostaining as indicated in Table S2. Gels were re-expanded in ddH₂O overnight before imaging. Expansion factors ranged from 4.2 to 4.6 and were calculated from the ratio between final gel size and the original 12 mm coverslip diameter.

Expanded samples were imaged on either a Leica TCS SP8 equipped with a 100×/1.40 oil objective or a Leica Stellaris 8 FALCON equipped with a 63×/1.20 water objective. For each condition, z-stacks of typically 17–30 cells were acquired under identical settings. Deconvolution was performed using Leica Lightning or Huygens Essential with identical settings within each dataset.

### U-ExM image analysis and quantitative measurements

Distances were measured using Fiji, and volumes were quantified using Imaris 9.5. In Imaris, MTOC structures were detected using Spot objects, while kinetochore, spindle and nuclear volumes were segmented using Surface objects.

For microgametocytes, cells were identified during acquisition by the presence of axonemal microtubules visualised by α/β-tubulin staining, which also labels the spindles. Full-cell 3D reconstructions were used to quantify spindle microtubule volume, kinetochore volume, MTOC number, spindle-pole number, basal-body number, spindle length and the presence of “free” basal bodies. Spindle morphologies were classified manually as resolved/post-segregation, ongoing anaphase/segregating, or persistent failed spindles, using the criteria illustrated in Figure. S4F.

For meiotic zygotes and ookinetes, spindle length was defined as the distance between the two spindle-associated MTOCs. In mature 24 h ookinetes, SYTOX staining was additionally used to segment the nucleus. All distance and volume measurements were converted back to non-expanded dimensions using the experimentally determined expansion factor.

### Flow cytometry

Blood-stage reporter retention was quantified by conventional flow cytometry. A small volume of tail blood was diluted into 500 μL PBS and analysed on a Beckman Coulter MoFlo Astrios EQ cell sorter using Summit v6.3.1 software. Events were first gated by forward- and side-scatter parameters displayed on a log scale to enrich erythrocytes, followed by singlet discrimination using FSC area versus FSC height. A further doublet-exclusion step was applied to the population of interest using SSC height versus SSC width. Fluorescence signals were then used to resolve lineage and reporter populations, with GFP detected using 488 nm excitation and a 526/52 emission filter, and BFP detected using 405 nm excitation and a 488/59 emission filter. For blood-stage analyses, infected cells were identified by GFP fluorescence from the male-only *fd*^*1*⁻^ background, and reporter retention was scored as the percentage of GFP⁺BFP⁺ events among total GFP⁺ singlets. Parental *fd*^*1*⁻^ parasites and the acentromeric PAC-AΔcen control were used to set negative gates and background thresholds. Data were analysed post-acquisition in FlowJo v11 to determine final population frequencies.

### Imaging-flow cytometry

Paternal inheritance after fertilisation was quantified by imaging-flow cytometry at 48 h after crossing. Cultures were first enriched for ookinetes on a Histodenz cushion prepared by mixing 2.5 mL of 27.6% Histodenz stock with 1.5 mL PBS, giving a final Histodenz concentration of 17.25%. The enriched isolate was then incubated on ice in 0.17 M ammonium chloride for 15 min to lyse residual erythrocytes, washed twice in PBS, and resuspended in fresh ookinete medium. The enriched isolate was then analysed on a BD FACSDiscover™ S8 Cell Sorter with CellView™ imaging technology, with acquisition and on-the-fly analysis performed in BD FACSChorus v6.2.8. Images were acquired for individual events, and gating was performed in an order designed to enrich mature ookinetes. First, events were selected based on light loss in the violet channel versus SSC-A to capture cells with the appropriate overall morphology. Singlets were then enriched using light-loss height versus light-loss width, followed by a second singlet/morphology gate using eccentricity versus radial moment derived from SSC measurements. Fluorescence gates were then applied using YG2 (590/16 band-pass filter) for mScarlet-I and B2 (517/16 band-pass filter) for GFP. Mature ookinetes were subsequently identified from the acquired images by their characteristic banana-shaped morphology using eccentricity versus long-axis moment. Within this banana-shaped population, light-loss parameters were further used to assess cell size and refine the final gate. Final paternal inheritance was quantified from the red and green fluorescent populations, with paternal retention calculated as the proportion of GFP⁺ mature ookinetes that were mScI⁺. Representative reporter-positive and reporter-negative ookinetes were additionally documented by live fluorescence microscopy.

### Chromosome inheritance assay and retention calculations

To compare chromosome inheritance across lifecycle stages, reporter constructs were introduced into the male-only *fd*^*1−*^ line and analysed during serial blood-stage propagation and after fertilisation following crosses to untransformed female-only *md*^*4−*^ parasites. In this assay, GFP identifies the *fd*^*1−*^*/md*^*4−*^ lineage background, whereas mBFP2 and mScI report inheritance of the marked locus itself. The acentromeric control PAC-AΔcen was used to confirm centromere dependence of PAC maintenance and to define background reporter thresholds.

#### Blood-stage retention

After transfection, parasites were cultured for 8 h, and GFP⁺mBFP2⁺ parasites were enriched by flow sorting. To monitor blood-stage maintenance, sorted parasites were serially passaged through mice. Each passage lasted 9 days and was sampled at day 7 and day 9. At the end of each passage, reporter-positive parasites were re-sorted to find the next passage. The values plotted in Fig. 2C therefore represent passage-specific retention values measured within each passage rather than cumulative retention across the whole experiment.

At each blood-stage timepoint, retention of the reporter locus was calculated as:

1 $${{{\rm{Blood}}}}{\mbox{-}}{{{\rm{stage\; retention}}}}(\%)=100\,\times ({{{\rm{GFP}}}}+{{{\rm{BFP}}}}+{{{\rm{singlets}}}}/{{{\rm{total\; GFP}}}}+{{{\rm{singlets}}}})$$

Passage-specific retention was calculated separately for each biological replicate and construct. Mean values across replicates were plotted in Fig. 2C together with replicate-level measurements.

#### Late-phase cumulative retention and retention per nuclear division

Because early post-transfection parasite populations are expected to contain variable PAC copy numbers, stable centromere-dependent maintenance was not inferred from the initial decline in reporter frequency. Instead, only the later approximately linear loss phase was used. The day-18 timepoint (the day-9 endpoint of passage P1) was defined as the start of this late phase and normalised to 1.

Late-phase cumulative retention was reconstructed from the day-9 endpoints of successive late passages using passage-specific retention values expressed as fractions. Thus:

2 $${C}_{18}=1,{C}_{25}={p}_{25},{C}_{32}={p}_{25}\times {p}_{32}$$

More generally, from the start of the late phase:

3 $${Ct}=\prod i=1{k\,pi}$$

where *pi* values are the successive day-9 passage-specific retention fractions after day 18, and *Ct* is the cumulative retention fraction at time point *t*.

Retention per nuclear division was then calculated from the reconstructed cumulative retention using the same multiplicative logic as Iwanaga et al.:

4 $$r=100\times {({Ct}/100)}^{1/n}$$

where r is retention per nuclear division, Ct is the cumulative late-phase retention expressed as a fraction, and n is the number of nuclear divisions elapsed over the same interval. Following Iwanaga et al., one 24 h P. berghei blood-stage cycle was assumed to comprise four nuclear divisions. Therefore:

5 $$n=4\times \Delta d$$

where Δ*d* is the number of days elapsed since the start of the late phase. Retention per nuclear division was calculated separately for each biological replicate from its late-phase cumulative value, and the mean, standard deviation and standard error of the mean were then derived from these replicate-specific values.

The acentromeric PAC-AΔcen control was rapidly lost before the late phase and was therefore used as a non-centromeric negative control rather than for late-phase maintenance estimates.

#### Paternal inheritance after fertilisation

For sexual-stage inheritance assays, reporter-positive male-only *fd*^*1−*^ lines were crossed to untransformed female-only *md*^*4−*^ parasites and cultured to mature ookinetes. Because the constitutive HSP70-driven mBFP2 signal was weak or not reliably detectable in mature ookinetes, paternal inheritance after fertilisation was quantified using the P28-driven mScI reporter. Mature banana-shaped GFP⁺ ookinetes were identified by imaging-flow cytometry and confirmed by fluorescence microscopy. Paternal transmission was calculated as:

6 $${{{\rm{Paternal\; transmission}}}}\,(\%)=	 100\times ({{{{\rm{GFP}}}}}^{+}{{{{\rm{mScI}}}}}^{+}{{{\rm{mature\; ookinetes}}}}/ \\ 	 {{{\rm{total}}}}\,{{{{\rm{GFP}}}}}^{+}{{{\rm{mature\; ookinetes}}}})$$

The endogenous SIL6 reporter was analysed in parallel as a single-copy chromosomal reference using the same fluorescent logic. All blood-stage and sexual-stage comparisons were performed from matched biological replicates.

### Immunopurification of ARK1 complexes

All ARK1 interactomes were generated by direct anti-HA immunoprecipitation of endogenously HA-tagged proteins; no proximity-labelling enzyme was used in this study.

Male-stage interactomes were obtained from activated male-only *fd*^*1−*^*::ARK1-3xHA* gametocytes. Meiotic interactomes were obtained from female-only *md*^*4−*^*::ARK1-3xHA* parasites after crossing to untagged *fd*^*1−*^ males and harvesting at 8 h post-fertilisation. Where indicated, mixed-sex gametocyte samples from *2.34::ARK1-3xHA* parasites were harvested 6 min post-activation.

For each sample, parasites were washed in HKMEG buffer (150 mM KCl, 150 mM glucose, 25 mM HEPES, 4 mM MgCl₂, 1 mM EGTA, pH 7.8) supplemented with protease inhibitors and MG132. To distinguish stable from more labile associations, samples were processed in parallel under native conditions or after limited formaldehyde cross-linking (0%, 0.1% or 1% formaldehyde for 10 min at room temperature), followed by quenching with glycine. Cross-linking was used only to stabilise interactions during lysis and purification and should not be interpreted as proximity labelling.

Cells were lysed in HKMEG containing 1% NP-40, 0.5% sodium deoxycholate, 0.1% SDS, 1 mM DTT, protease inhibitors and MG132. Lysates were sonicated in a water-bath sonicator, clarified by centrifugation, and incubated for 4 h with affinity-purified anti-HA antibody coupled to Protein G Dynabeads. Beads were washed extensively and bound proteins were processed for on-bead digestion.

#### Sample preparation for mass spectrometry

Bead-bound proteins were resuspended in 6 M urea/50 mM ammonium bicarbonate, reduced with dithioerythritol, alkylated with iodoacetamide, diluted to 1 M urea, and digested overnight at 37 °C with trypsin. Peptides were desalted on C18 microspin columns, dried under vacuum and stored at −20 °C until LC-MS/MS analysis.

#### LC-MS/MS acquisition

Peptides were analysed by LC-ESI-MS/MS on either a Q Exactive Plus Orbitrap mass spectrometer coupled to an Easy-nLC 1000 or an Orbitrap Fusion Lumos coupled to an Easy-nLC 1200. Peptides were trapped on an Acclaim PepMap 100 nano trap column and separated on Easy-Spray C18 analytical columns using a 90 min gradient of solvent A (0.1% formic acid in water) and solvent B (0.1% formic acid in acetonitrile) at 250 nL/min.

For the Q Exactive Plus, MS1 scans were acquired at 70,000 resolution followed by HCD MS2 scans of up to 15 selected precursors. For the Orbitrap Fusion Lumos, MS1 scans were acquired in the Orbitrap at 120,000 resolution and data-dependent MS2 spectra were acquired in the ion trap within a 3 s cycle time.

#### Protein identification and quantitative interactome analysis

Raw files were processed in Proteome Discoverer 2.3 and searched with Mascot against the *P. berghei* ANKA database (PlasmoDB release 46) supplemented with a database of common contaminants. Trypsin specificity with up to one missed cleavage was allowed. Carbamidomethylation of cysteine was set as a fixed modification, and methionine oxidation and phosphorylation of serine, threonine and tyrosine were allowed as variable modifications. Protein identification was filtered using a target-decoy strategy at 1% false discovery rate at the PSM, peptide and protein levels.

Relative enrichment of interactors was assessed from normalised spectral abundance values (NSAF) and compared across ARK1-3xHA, AKiT1-3xHA and the corresponding no/low/high cross-link conditions, as indicated in the relevant figures and supplementary tables. Proteins reproducibly enriched in ARK1 pulldowns relative to controls were annotated by functional class and visualised in R.

### Comparative INCENP analyses

Representative INCENP proteins from apicomplexans and selected alveolate outgroups were curated from public genome resources and manually checked prior to analysis. Full-length proteins and IN-box-only regions were analysed separately.

Multiple sequence alignments (MAFFT) were generated for the conserved regions of INCENP, and maximum-likelihood phylogenies (IQTREE) were inferred from the conserved full-length alignment. Node support was assessed using SH-aLRT and ultrafast bootstrap support. Coiled-coil segments were predicted with ncoils and displayed alongside manually curated N-terminal, C-terminal and IN-box architectures.

To compare cross-lineage similarity classes independently of variable low-complexity and coiled-coil regions, lineage-specific HMMs (HMMER) were generated either from full-length INCENP proteins or from the IN-box alone. These profiles were compared by profile–profile searches using HHsearch. For each query, Δ(*A* − *B*) values were calculated as:

7 $$\Delta (A-B)=-{\log }_{10}({E}_{A})-[-\log 10({E}_{B})]$$

where *E*_*A*_ and *E*_*B*_ are the HHsearch E-values obtained against the two reference classes being compared. Constrained topologies representing parallel-duplication and ancient-core duplication scenarios were then tested on the conserved alignment using approximately unbiased (AU) tests.

### Statistics and reproducibility

Statistical analyses were performed in R. Unless otherwise stated, continuous variables were compared using two-sided Wilcoxon rank-sum tests and categorical distributions were compared using chi-squared tests. No adjustments for multiple comparisons were applied unless explicitly stated. Exact *P* values for statistical comparisons are provided in the Source Data file. Significance thresholds were defined as ns, *P* ≥ 0.05; *P < 0.05; **P < 0.01; ***P < 0.001. Boxplots show the median centre line, interquartile-range box and 1.5 × IQR whiskers, with individual observations overlaid unless otherwise stated. Error bars, where shown, indicate mean ± SEM. Principal-component analysis was performed on centred and scaled quantitative U-ExM features using the first two principal components for visualisation.

No statistical method was used to predetermine sample size. Sample sizes were chosen based on previous work in P. berghei sexual-stage imaging, mosquito-transmission and U-ExM assays, and on the number of biological replicates, mosquito midguts, oocysts, pooled sporozoite samples or fully reconstructed cells required to capture reproducible stage-specific phenotypes. Biological replicate numbers, sample sizes, units of analysis and statistical tests are provided in the figure legends and Source Data file. For imaging-based quantifications, the unit of analysis was the individual cell, ookinete, oocyst, midgut or pooled organ homogenate as defined in the relevant figure legend. For exflagellation and parasite-line comparison assays, each independently prepared mouse-derived parasite sample was treated as one biological replicate. For mosquito-transmission assays, independent mosquito feeds were treated as biological replicates. Representative imaging experiments were repeated independently with similar results as stated in the relevant figure legends.

Samples were allocated to experimental groups according to parasite genotype, reporter configuration, developmental stage and time point. Random allocation was not applicable because groups were defined by engineered parasite lines, matched parental controls or defined developmental stages. Blinding was not used for most experiments because experimental groups were identifiable from genotype, fluorescent reporter configuration or developmental phenotype during acquisition and analysis. Where flow-cytometry gates, imaging-flow gates, segmentation thresholds or quantitative image-analysis settings were used, the same analysis criteria were applied consistently across compared groups.

### Software and data processing

Kinetochore, spindle and protein architecture schematics are author-created in Inkscape 1.4.2. Flow cytometry data were acquired using Summit v6.3.1 on a Beckman Coulter MoFlo Astrios EQ cell sorter and analysed using FlowJo v11. Imaging-flow-cytometry data were acquired and analysed on a BD FACSDiscover™ S8 Cell Sorter with CellView™ imaging technology using BD FACSChorus v6.2.8. Live-cell fluorescence microscopy was performed on an inverted Zeiss Axio Observer Z1 microscope and mosquito-stage widefield imaging on a Leica DM5500 microscope; confocal and U-ExM imaging were performed on Leica TCS SP8 and Leica Stellaris 8 FALCON systems. Microscope acquisition was performed using ZEN 3.11 and LAS X (Leica Application Suite X). Image processing, measurements and figure preparation were performed using Fiji/ImageJ version 2.17.0 and Imaris v9.5. Where deconvolution was applied, images were processed using Leica Lightning version 4.1.1 or Huygens Essential v25.10.

Proteomics raw files were processed using Proteome Discoverer v2.3 and searched with Mascot 3.x against the *Plasmodium berghei* ANKA database from PlasmoDB release 46, supplemented with common contaminants. Comparative sequence analyses were performed using MAFFT 7.526, IQ-TREE 3.1.1, HMMER 3.4, HHsearch/HHsuite 3.3.0 and ncoils/COILS implementation of Lupas et al. Statistical analysis and plotting were performed in R 4.6.0 using ggplot2.

### Reporting summary

Further information on research design is available in the Nature Portfolio Reporting Summary linked to this article.

## Supplementary information

Supplementary Information

Description Of Additional Supplementary File

Supplementary Data 1

Supplementary Data 2

Supplementary Data 3

Supplementary Data 4

Supplementary Data 5

Supplementary Data 6

Supplementary Data 7

Supplementary Data 8

Supplementary Data 9

Supplementary Data 10

Supplementary Data 11

Supplementary Data 12

Supplementary Video 1

Supplementary Video 2

Supplementary Video 3

Supplementary Video 4

Supplementary Video 5

Supplementary Video 6

Supplementary Video 7

Supplementary Video 8

Reporting Summary

Transparent Peer Review file

## Source data

Source Data

## Data Availability

All data supporting the findings of this study are available within the paper, the Supplementary Information, the Source Data files, the Supplementary Data files, and associated public repositories. Source Data are provided with this paper. Proteomics mass spectrometry data generated in this study have been deposited in the PRIDE database under accession code PXD068832. Supplementary Data 1–9 contain quantitative measurements, raw counts, interactome outputs, comparative sequence datasets, and related source datasets. Supplementary Data 10–12 contain full U-ExM 3D reconstructions and additional representative imaging datasets. Supplementary Tables S1–S2 contain oligonucleotide, genetic construct, antibody, stain, and reagent details. Source data are provided with this paper.

## Code availability

No custom software or mathematical algorithm central to the conclusions of this study was developed. Statistical analyses, data visualisation and figure generation were performed using standard software, packages and functions as described in the Methods. The numerical data required to reproduce the analyses and figures are provided in the Source Data file and Supplementary Data files.
