# Supplementary material for: A modular chromosomal passenger complex rewires chromosome segregation in Plasmodium berghei
Source: Nat Commun. 2026 Jun 20;17:7788. doi: 10.1038/s41467-026-74542-7 (PMC13439184; doi:10.1038/s41467-026-74542-7)
Supplement: Supplementary file 1 [file 41467_2026_74542_MOESM1_ESM.pdf]

Supplementary information

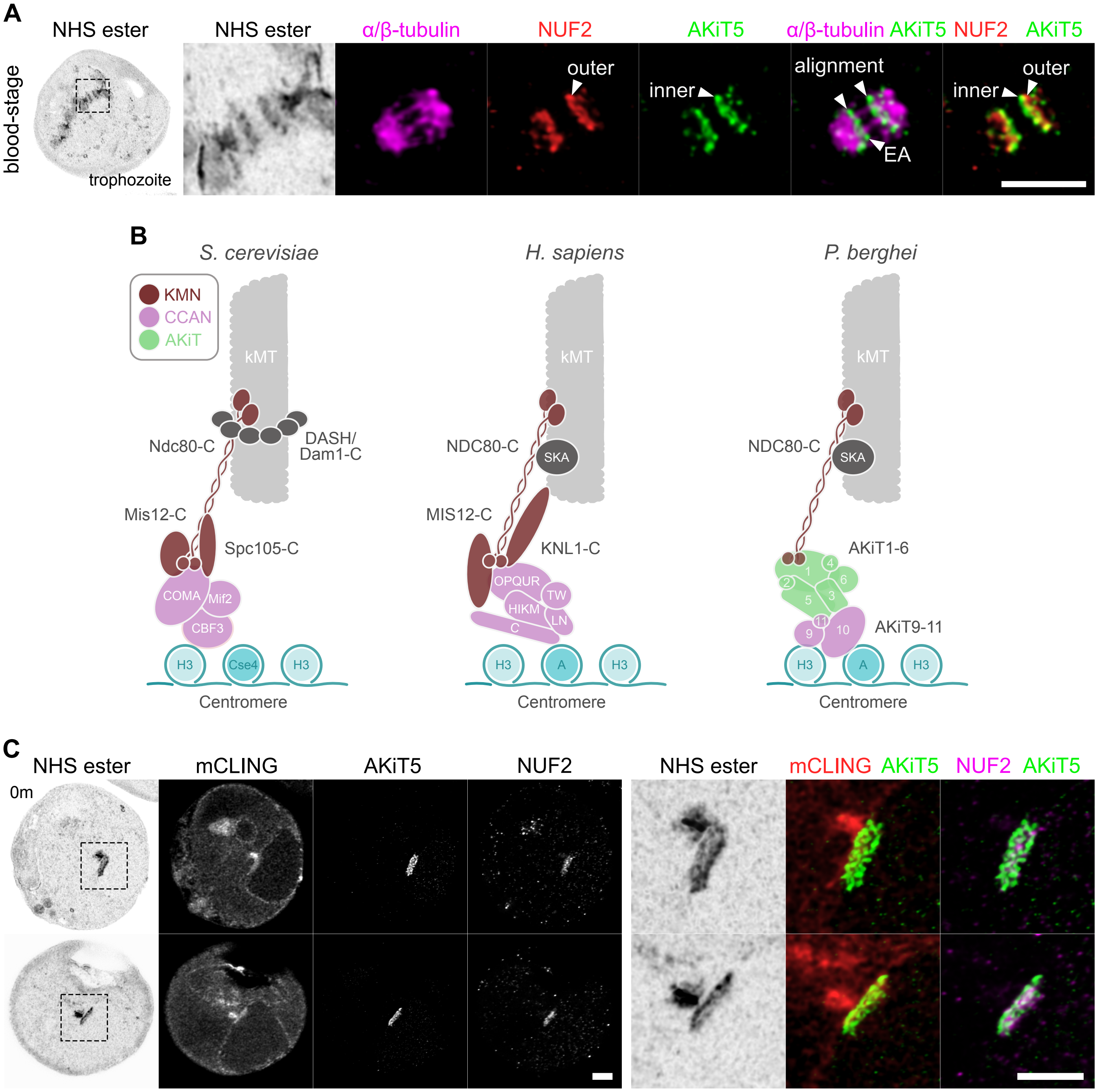
A
blood-stage
NHS ester
trophozoite
NHS ester
α/β-tubulin
NUF2
outer
AKiT5
inner
α/β-tubulin AKiT5
alignment
EA
NUF2 AKiT5
inner
outer
B
S. cerevisiae
KMN
CCAN
AKiT
kMT
Ndc80-C
DASH/
Dam1-C
Mis12-C
Spc105-C
COMA
Mif2
CBF3
H3
Cse4
H3
Centromere
H. sapiens
kMT
NDC80-C
SKA
MIS12-C
KNL1-C
OPQUR
TW
HIKM
LN
C
H3
A
H3
Centromere
P. berghei
kMT
NDC80-C
SKA
AKiT1-6
AKiT9-11
H3
A
H3
Centromere
C
NHS ester
0m
mCLING
AKiT5
NUF2
NHS ester
mCLING AKiT5
NUF2 AKiT5

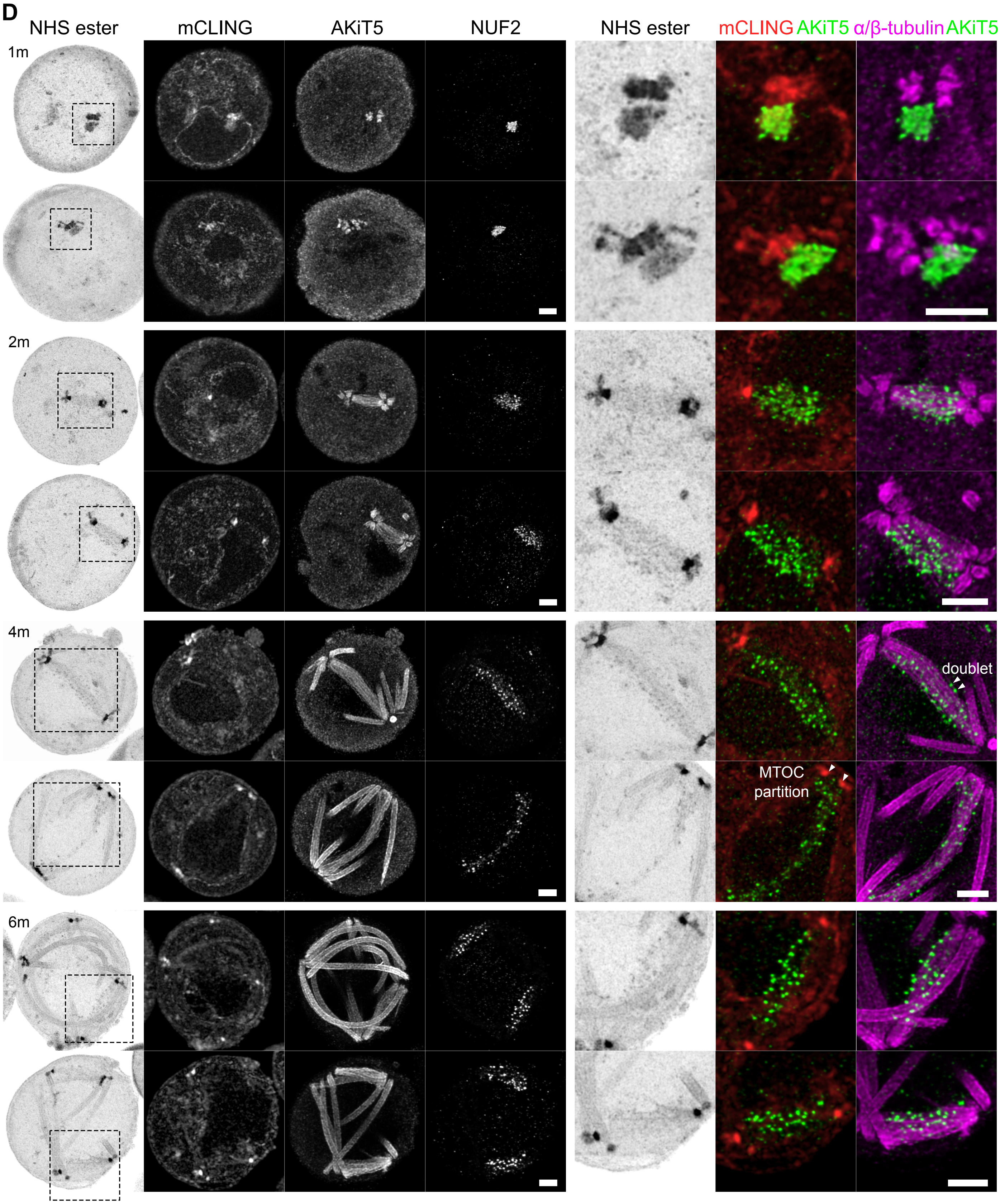
D
NHS ester
mCLING
AKiT5
NUF2
NHS ester
mCLING AKiT5
α/β-tubulin AKiT5
1m
2m
4m
6m
doublet
MTOC
partition

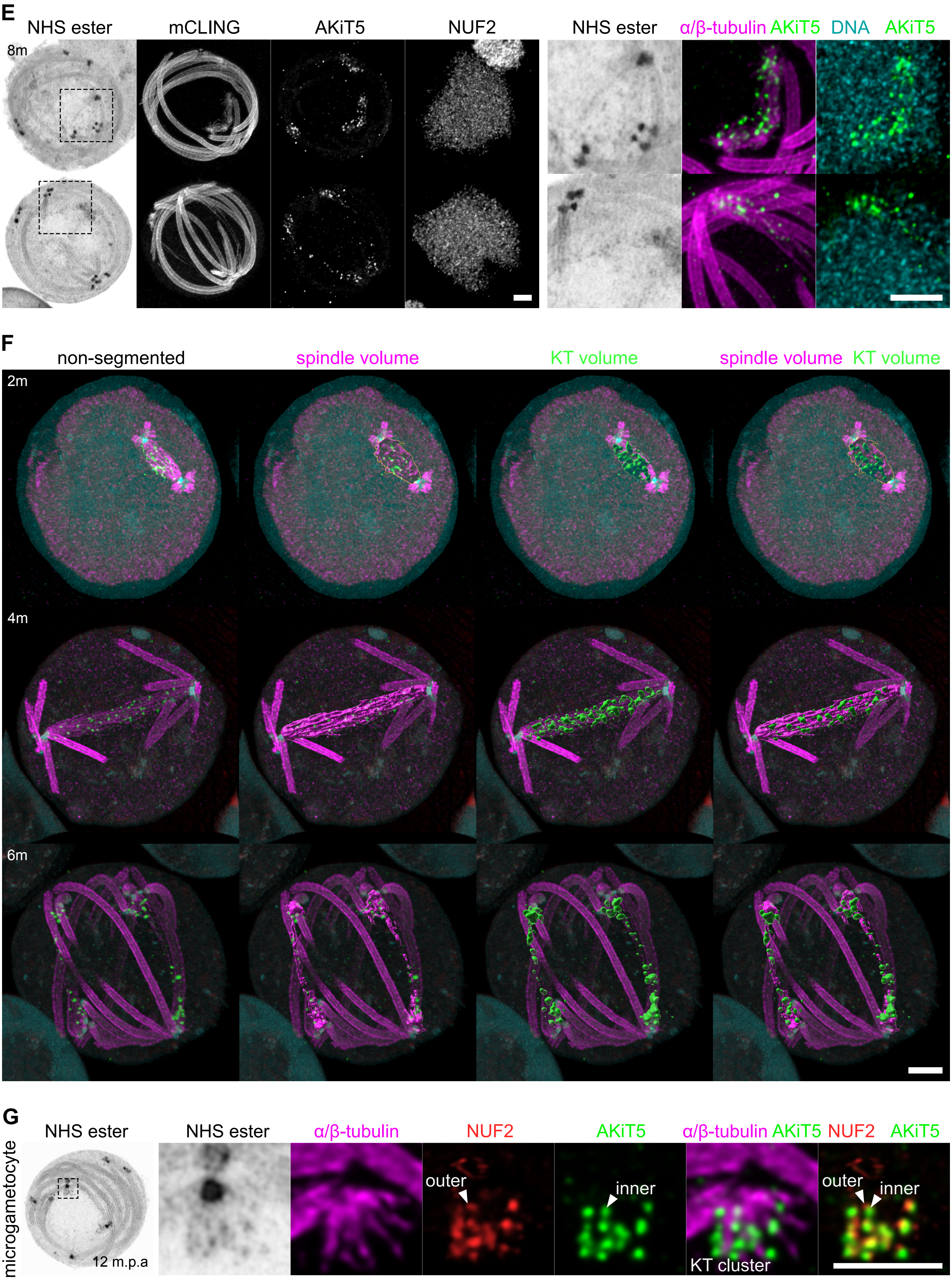

E
NHS ester
mCLING
AKiT5
NUF2
NHS ester
α/β-tubulin AKiT5
DNA AKiT5
8m
F
non-segmented
spindle volume
KT volume
spindle volume KT volume
2m
4m
6m
G
microgametocyte
NHS ester
NHS ester
α/β-tubulin
NUF2
AKiT5
α/β-tubulin AKiT5
NUF2 AKiT5
12 m.p.a
outer
inner
outer
inner
KT cluster

Figure S1. Comparative kinetochore organisation and spindle dynamics across asexual and sexual stages of *P. berghei*. (A) U-ExM of an asexual intra-erythrocytic trophozoite expressing AKiT5-mNG-3xHA, co-stained for α/β-tubulin and NUF2. Images show metaphase-like kinetochore alignment, end-on attachment (EA) to spindle microtubules, and separation of inner AKiT5 and outer NUF2 kinetochore domains. (B) Comparative schematic of kinetochore architectures in *S. cerevisiae*, *H. sapiens* and *P. berghei*, highlighting conserved KMN/CCAN modules and the streamlined AKiT-containing kinetochore in *P. berghei*. (C) Early microgametocyte activation at 0 min post-activation showing the bipartite MTOC, nuclear-envelope-associated membrane staining, and clustered kinetochores within the inner nuclear body. (D) Time-resolved U-ExM analysis of microgametogenesis at 1, 2, 4 and 6 min post-activation, showing MTOC partitioning, spindle elongation and lateral redistribution of kinetochores along spindle microtubules. (E) Late microgametogenesis at 8 min post-activation showing multiple bipolar spindles and dispersed kinetochore foci. (F) Representative full-cell 3D reconstructions used for spindle and kinetochore volumetric quantification in Fig. 1. Segmentation masks for spindle microtubules and kinetochores are overlaid. (G) Terminal microgametogenesis at 12 min post-activation compared with an asexual trophozoite, showing eight discrete kinetochore clusters rather than metaphase-like alignment. Scale bars, 1 µm; dimensions are non-expanded.

A

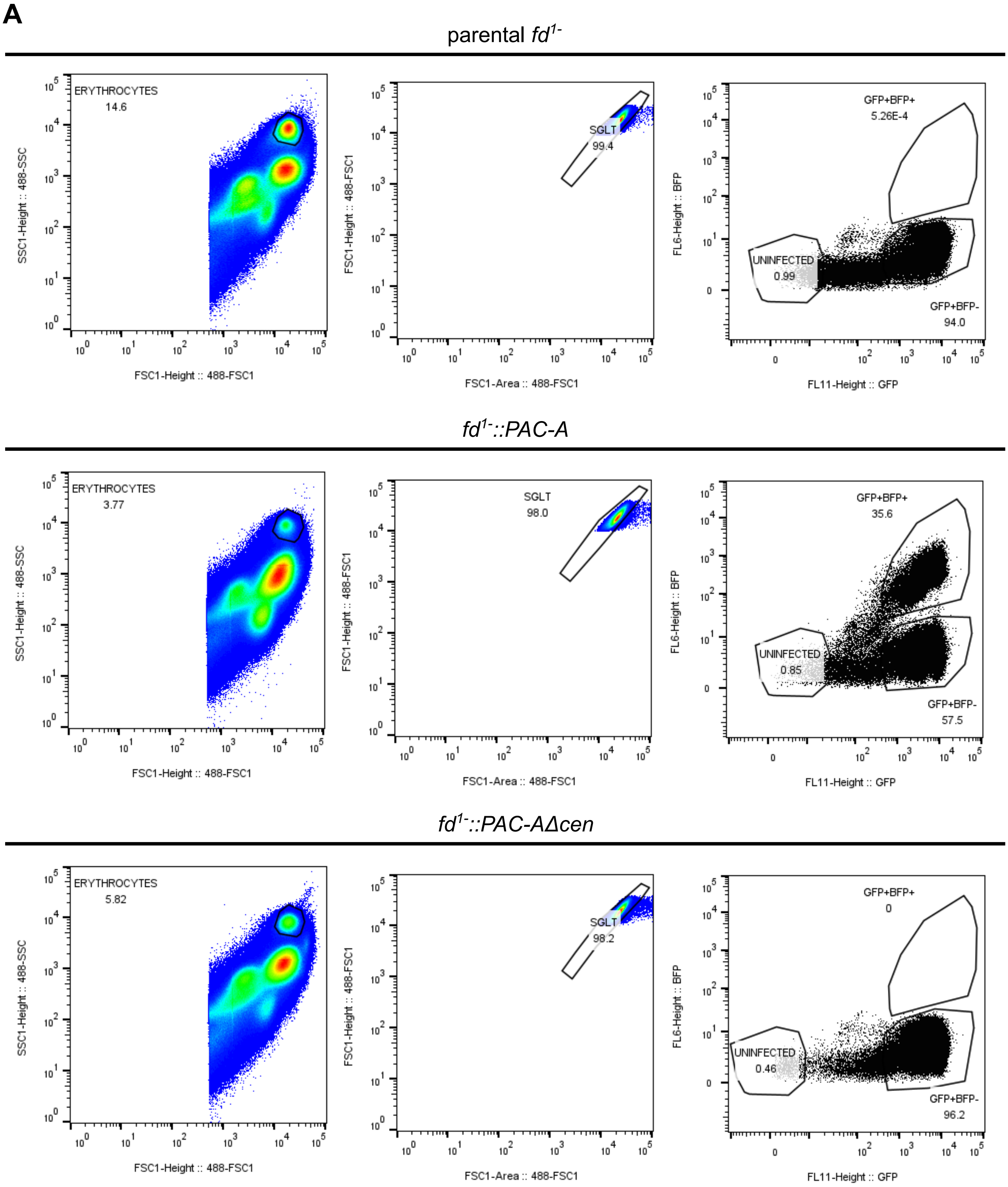
parental fd1-
ERYTHROCYTES
14.6
SSC1-Height :: 488-SSC
FSC1-Height :: 488-FSC1
SGLT
99.4
FSC1-Height :: 488-FSC1
FSC1-Area :: 488-FSC1
GFP+BFP+
5.26E-4
UNINFECTED
0.99
GFP+BFP-
94.0
FL6-Height :: BFP
FL11-Height :: GFP
fd1-::PAC-A
ERYTHROCYTES
3.77
SGLT
98.0
GFP+BFP+
35.6
UNINFECTED
0.85
GFP+BFP-
57.5
fd1-::PAC-AΔcen
ERYTHROCYTES
5.82
SGLT
98.2
GFP+BFP+
0
UNINFECTED
0.46
GFP+BFP-
96.2

B

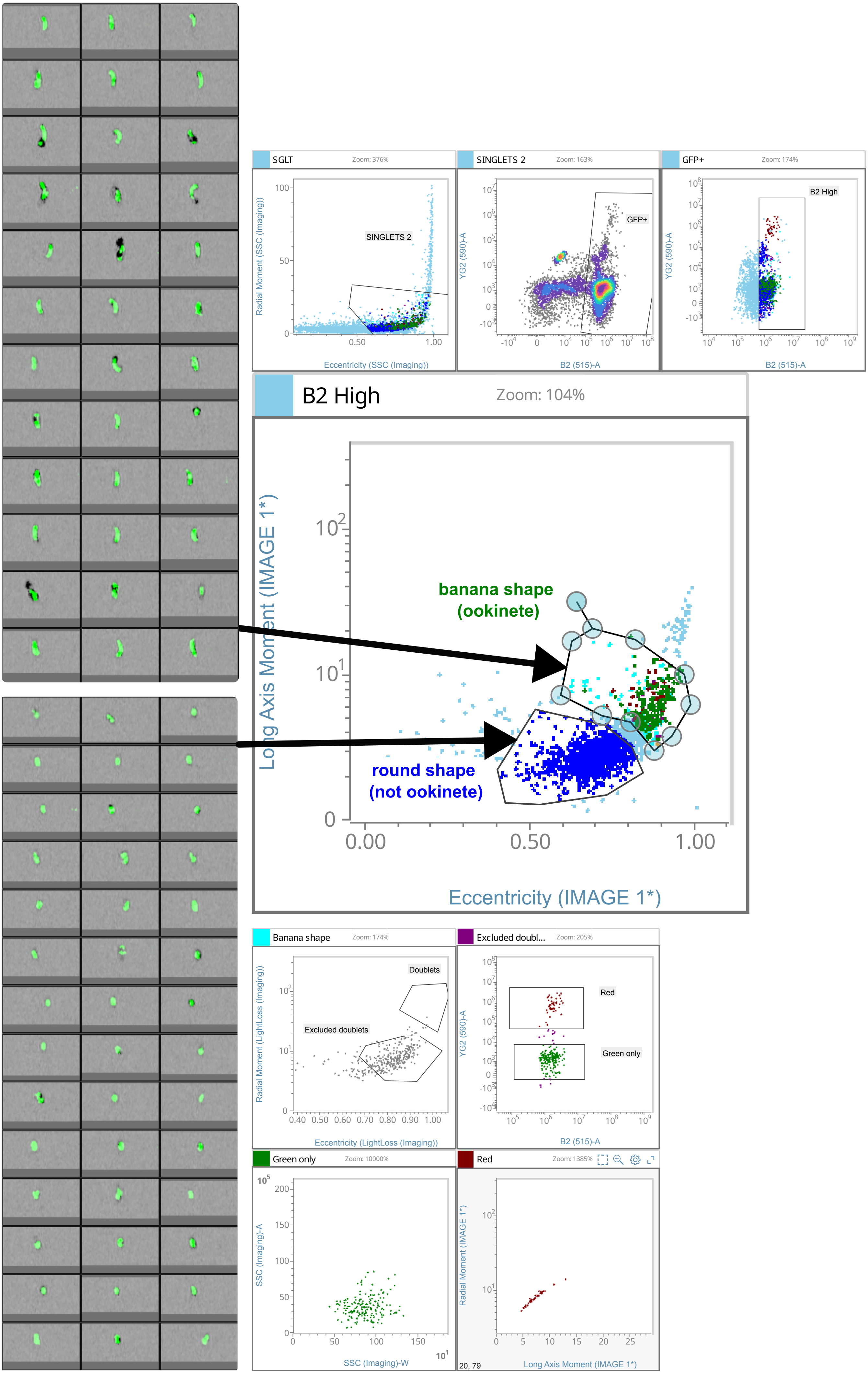

SGLT
Zoom: 376%
SINGLETS 2
Radial Moment (SSC (Imaging))
Eccentricity (SSC (Imaging))
SINGLETS 2
Zoom: 163%
GFP+
YG2 (590)-A
B2 (515)-A
GFP+
Zoom: 174%
B2 High
YG2 (590)-A
B2 (515)-A
B2 High
Zoom: 104%
banana shape
(ookinete)
round shape
(not ookinete)
Long Axis Moment (IMAGE 1*)
Eccentricity (IMAGE 1*)
Banana shape
Zoom: 174%
Doublets
Excluded doublets
Radial Moment (LightLoss (Imaging))
Eccentricity (LightLoss (Imaging))
Excluded doubl...
Zoom: 205%
Red
Green only
YG2 (590)-A
B2 (515)-A
Green only
Zoom: 10000%
SSC (Imaging)-A
SSC (Imaging)-W
Red
Zoom: 1385%
Radial Moment (IMAGE 1*)
Long Axis Moment (IMAGE 1*)
20, 79

Figure S2. Flow cytometry and imaging-flow cytometry workflows used in the chromosome inheritance assay. (A) Representative flow cytometry gating strategy used to quantify reporter inheritance in blood-stage parasites. Cells were selected by forward- and side-scatter properties, followed by singlet discrimination. GFP and BFP fluorescence were then used to distinguish uninfected cells, $GFP^{+}BFP^{-}$ parasites and $GFP^{+}BFP^{+}$ reporter-retaining parasites. Example gates are shown for parental *fd*$^{1-}$ parasites, *fd*$^{1-}$*::PAC-A* and the centromere-deficient control *fd*$^{1-}$*::PAC-AΔcen*. (B) Imaging-flow cytometry workflow used to distinguish mature elongated ookinetes from round sexual-stage cells. Single-cell events were selected, reporter-positive populations identified, and mature ookinetes classified using eccentricity and long-axis moment measurements. Doublets and colour-specific subpopulations were excluded before final scoring.

Panel A shows the sequential gating strategy used for blood-stage reporter-retention measurements and sorting of $GFP^{+}BFP^{+}$ parasites in Fig. 2C and the blood-stage component of Fig. 2D. Panel B shows the sequential imaging-flow cytometry gating strategy used to identify mature ookinetes and quantify paternal reporter inheritance after fertilisation in Fig. 2D.

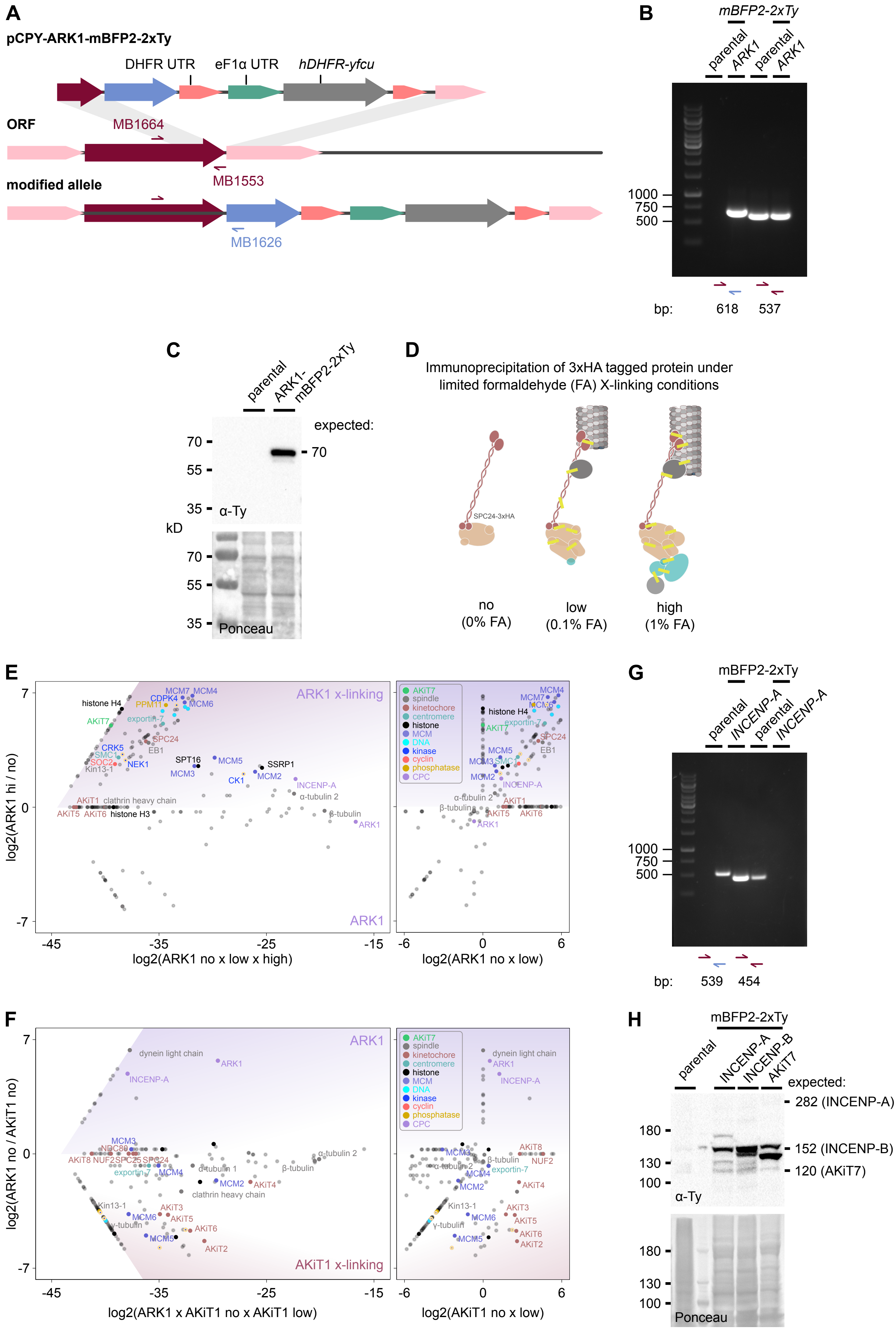
A
pCPY-ARK1-mBFP2-2xTy
DHFR UTR
eF1α UTR
hDHFR-yfcu
ORF
MB1664
MB1553
modified allele
MB1626
B
mBFP2-2xTy
parental
ARK1
parental
ARK1
1000
750
500
bp:
618
537
C
parental
ARK1-mBFP2-2xTy
expected:
70
55
35
α-Ty
kD
70
55
35
Ponceau
D
Immunoprecipitation of 3xHA tagged protein under limited formaldehyde (FA) X-linking conditions
SPC24-3xHA
no (0% FA)
low (0.1% FA)
high (1% FA)
E
ARK1 x-linking
ARK1
log2(ARK1 hi / no)
log2(ARK1 no x low x high)
log2(ARK1 no x low)
AKiT7
spindle
kinetochore
centromere
histone
MCM
DNA
kinase
cyclin
phosphatase
CPC
F
ARK1
AKiT1 x-linking
log2(AKiT1 no / AKiT1 no)
log2(ARK1 x AKiT1 no x AKiT1 low)
log2(AKiT1 no x low)
G
mBFP2-2xTy
parental
INCENP-A
parental
INCENP-A
1000
750
500
bp:
539
454
H
mBFP2-2xTy
parental
INCENP-A
INCENP-B
AKiT7
expected:
282 (INCENP-A)
152 (INCENP-B)
120 (AKiT7)
180
130
100
α-Ty
Ponceau

Figure S3. Validation of ARK1 tagging and cross-linking-resolved interactome analysis. (A) Schematic of the endogenous C-terminal tagging strategy used to generate ARK1-mBFP2-2xTy parasites. The construct integrates mBFP2-2xTy at the 3′ end of the ARK1 open reading frame, together with the hDHFR-yfcu selection cassette and flanking homology regions. Primer positions used for diagnostic PCR are indicated. (B) Diagnostic PCR confirming integration at the ARK1 locus. Expected products of 618 bp and 537 bp are detected in tagged parasites but not in parental controls. (C) Anti-Ty immunoblot of ARK1-mBFP2-2xTy parasites. A band is detected at the expected molecular weight of approximately 70 kDa in tagged parasites but not parental controls; Ponceau S staining shows protein loading. (D) Schematic of 3xHA immunoprecipitation under native or limited formaldehyde cross-linking conditions. (E) ARK1 interactome under graded cross-linking conditions. Scatter plots show log2 enrichment of ARK1-associated proteins comparing no, low and high formaldehyde cross-linking. Functional classes are colour-coded as indicated. (F) Comparison of ARK1-3xHA and AKiT1-3xHA immunoprecipitations under native and cross-linked conditions, resolving stable ARK1–INCENP-A association from broader kinetochore-associated proteins enriched with AKiT1. (G) Diagnostic PCR confirming C-terminal tagging of INCENP-A with mBFP2-2xTy. Expected products of 539 bp and 454 bp are detected in tagged parasites but not parental controls. (H) Anti-Ty immunoblot of parasites expressing INCENP-A-mBFP2-2xTy. The INCENP-A lane is from the same blot shown in Fig. S6C. Anti-Ty does not clearly resolve a band at the expected molecular weight of approximately 282 kDa, consistent with the large size of INCENP-A and reduced transfer efficiency; Ponceau S staining shows protein loading. Source data and uncropped blot/gel images are provided as a Source Data file.

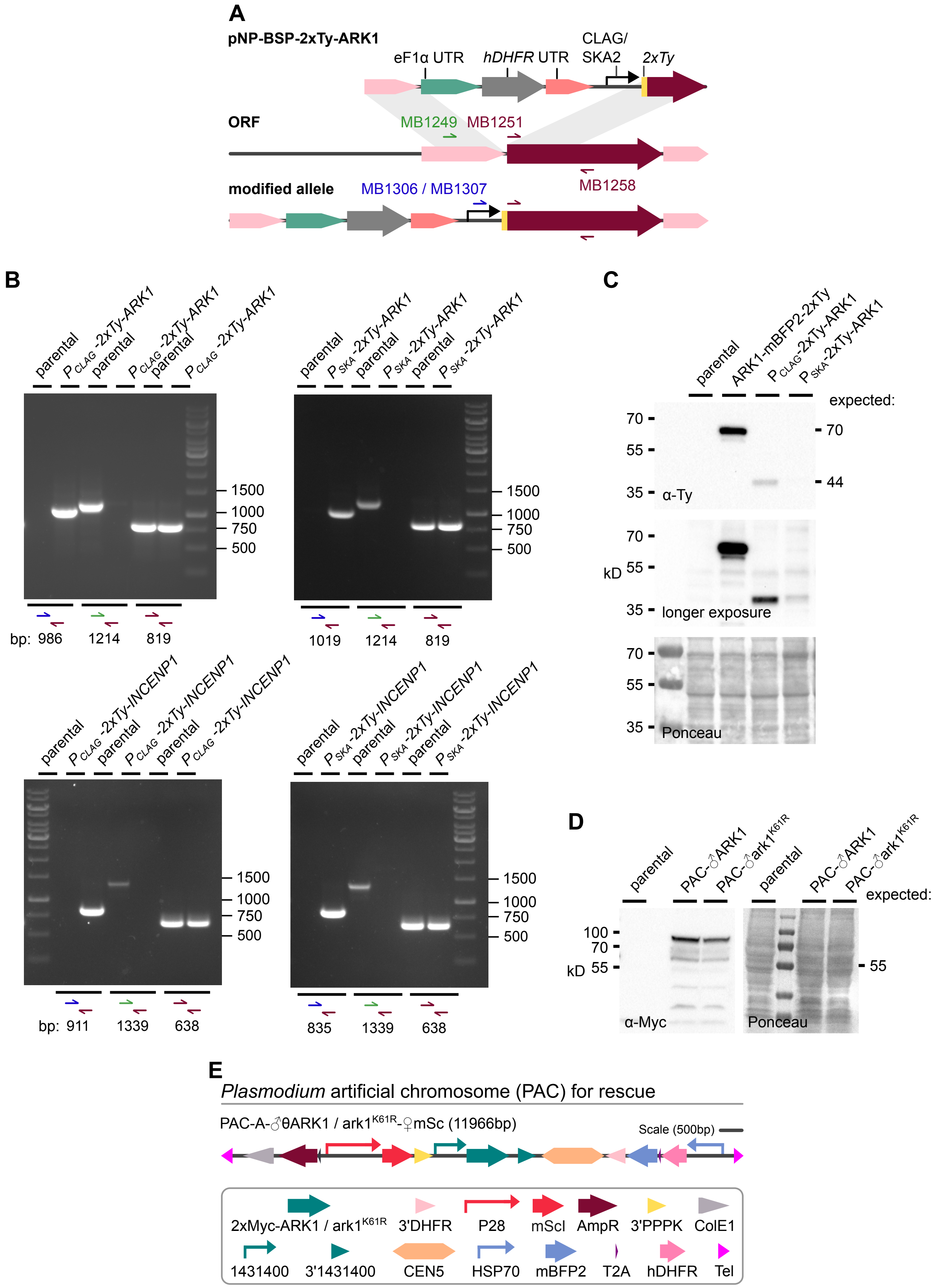
A
pNP-BSP-2xTy-ARK1
eF1α UTR
hDHFR UTR
CLAG/
SKA2
2xTy
ORF
MB1249
MB1251
MB1258
modified allele
MB1306 / MB1307
B
parental
P CLAG -2xTy-ARK1
P SKA -2xTy-ARK1
1500
1000
750
500
bp: 986
1214
819
1019
P CLAG -2xTy-INCENP1
P SKA -2xTy-INCENP1
bp: 911
1339
638
835
C
parental
ARK1-mBFP2-2xTy
P CLAG-2xTy-ARK1
P SKA-2xTy-ARK1
expected:
70
55
35
44
α-Ty
kD
longer exposure
Ponceau
D
parental
PAC-♂ARK1
PAC-♂ark1K61R
100
α-Myc
E
Plasmodium artificial chromosome (PAC) for rescue
PAC-A-♂θARK1 / ark1K61R-♀mSc (11966bp)
Scale (500bp)
2xMyc-ARK1 / ark1K61R
3'DHFR
P28
mScI
AmpR
3'PPPK
ColE1
1431400
3'1431400
CEN5
HSP70
mBFP2
T2A
hDHFR
Tel

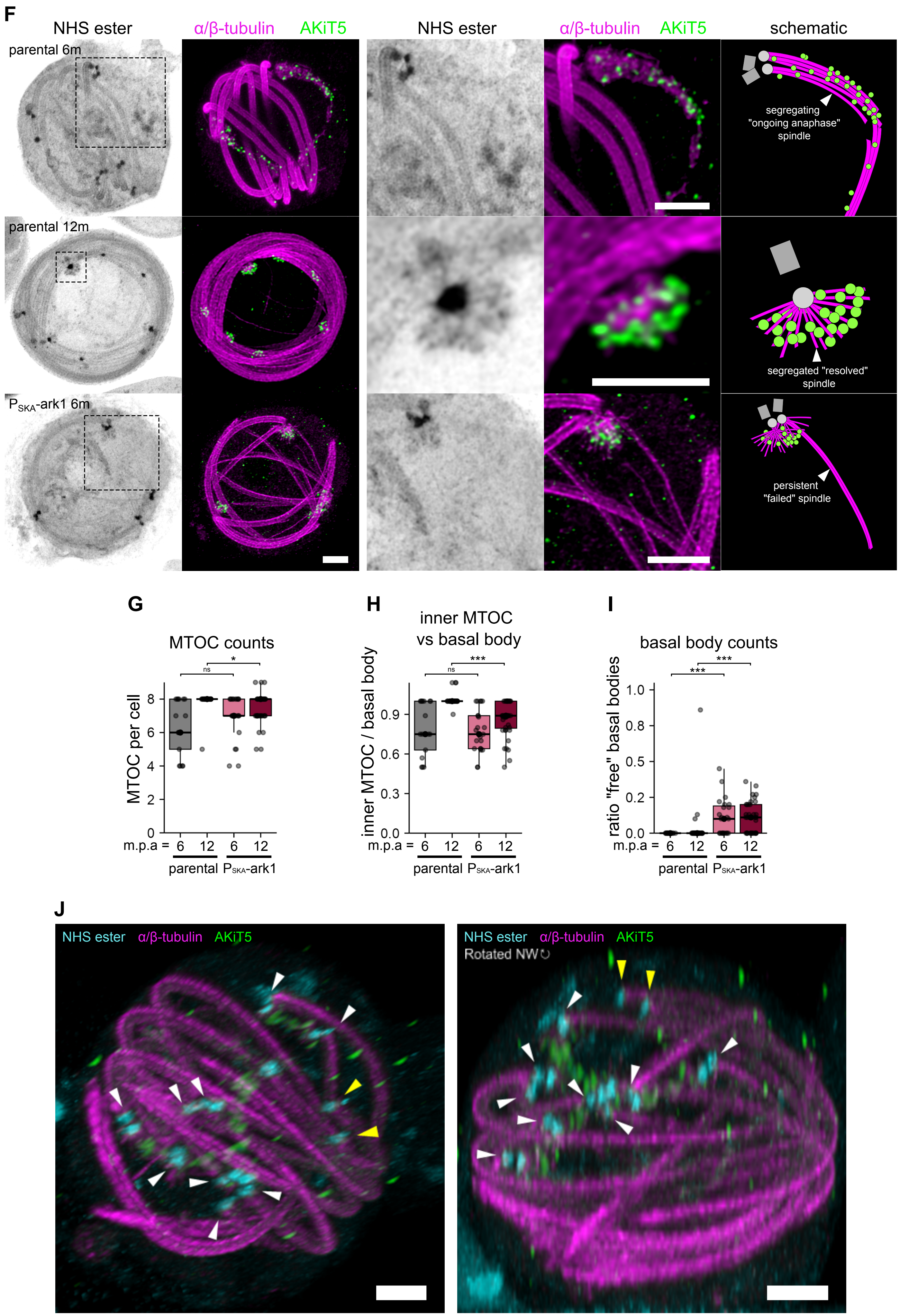

F
NHS ester
α/β-tubulin AKiT5
NHS ester
α/β-tubulin AKiT5
schematic
parental 6m
segregating
"ongoing anaphase"
spindle
parental 12m
segregated "resolved"
spindle
PSKA-ark1 6m
persistent
"failed" spindle
G
MTOC counts
MTOC per cell
m.p.a = 6 12 6 12
parental PSKA-ark1
H
inner MTOC
vs basal body
inner MTOC / basal body
m.p.a = 6 12 6 12
parental PSKA-ark1
I
basal body counts
ratio "free" basal bodies
m.p.a = 6 12 6 12
parental PSKA-ark1
J
NHS ester α/β-tubulin AKiT5
NHS ester α/β-tubulin AKiT5
Rotated NW

Figure S4. Validation of ARK1/INCENP-A depletion lines and quantitative analysis of MTOC–spindle organisation during microgametogenesis. (A) Promoter-swap strategy used to place ARK1 under control of CLAG or SKA2 regulatory sequences, with an N-terminal 2xTy tag. Primer positions used for diagnostic PCR are indicated. (B) Diagnostic PCR confirming integration of promoter-swap constructs for ARK1 and INCENP-A in PCLAG and PSKA lines. Expected amplicon sizes are indicated. (C) Anti-Ty immunoblot analysis of ARK1 promoter-swap lines, showing reduced ARK1 protein levels in P_{CLAG}-2xTy-ARK1 and P_{SKA}-2xTy-ARK1 parasites relative to endogenous ARK1-mBFP2-2xTy. Ponceau S staining shows protein loading. (D) Anti-Myc immunoblot of PAC-mediated rescue lines expressing 2xMyc-tagged ARK1 or ark1^{K61R} under the male-specific PBANKA_1431400 promoter. Ponceau S staining shows protein loading. (E) Schematic of the PAC-A-♂ARK1/ark1^{K61R}-♀mSc rescue construct. (F) Representative U-ExM micrographs illustrating spindle morphology classes in parental and P_{SKA}-ark1 microgametocytes: ongoing anaphase/segregating, resolved/post-segregation and persistent failed spindles. Scale bars, 1 µm; dimensions are non-expanded. (G–I) Quantification of total MTOC number, inner MTOC-to-basal-body ratio, and basal-body/free-basal-body organisation at 6 and 12 min post-activation. n = 19 parental 6 m.p.a., 31 parental 12 m.p.a., 22 P_{SKA}-ark1 6 m.p.a. and 40 P_{SKA}-ark1 12 m.p.a. fully reconstructed microgametocytes from three independent biological replicates; each cell is the unit of analysis. Boxplots show the median centre line, interquartile-range box and 1.5×IQR whiskers; points are individual cells. Statistical significance was assessed using two-sided Wilcoxon rank-sum tests with no adjustment for multiple comparisons; exact P values are provided in the Source Data file. (J) Representative U-ExM micrograph of P_{SKA}-ark1 microgametocytes displaying over-amplified and detached/free basal bodies. Source data and uncropped gel/blot images are provided as Source Data files.

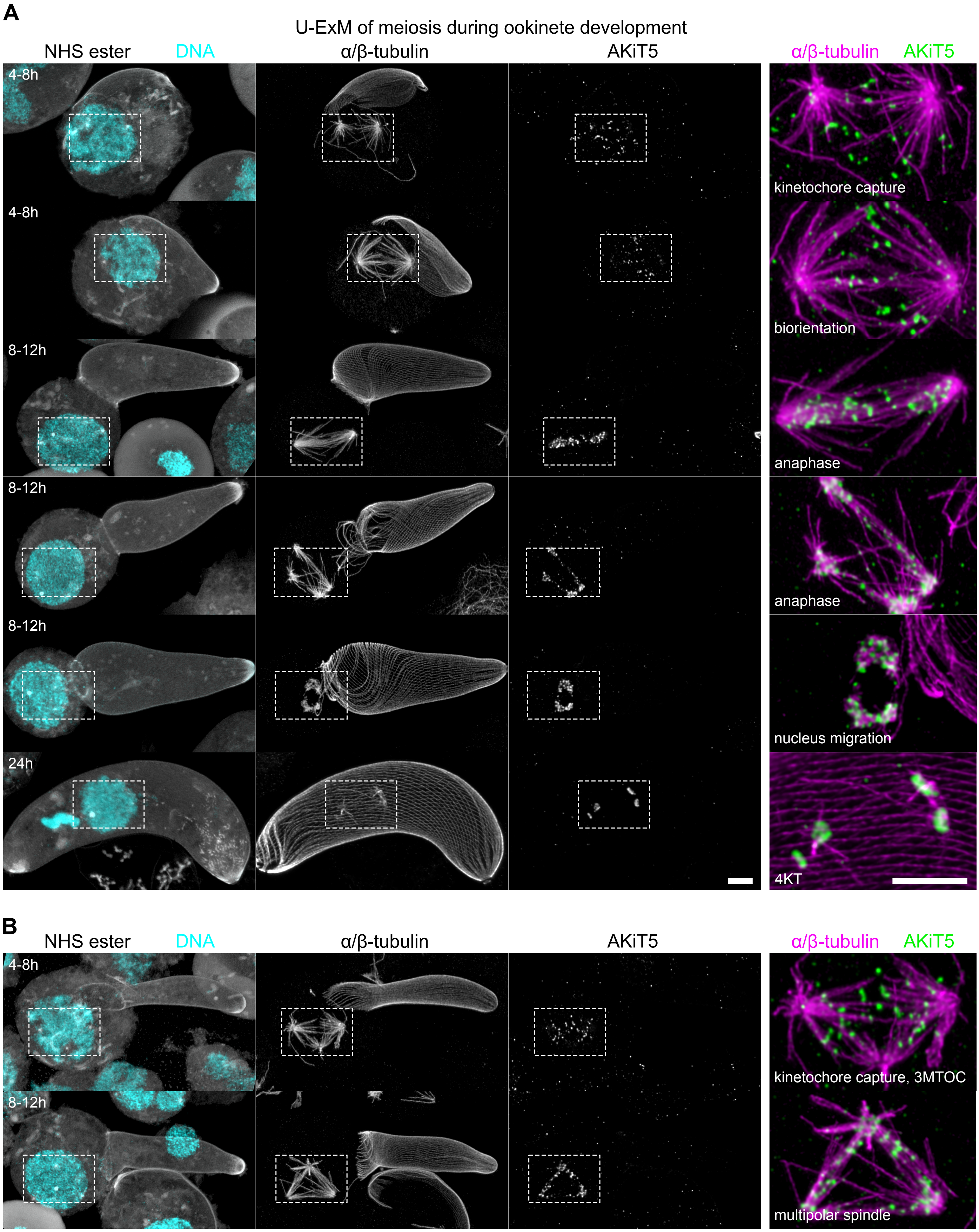
A
U-ExM of meiosis during ookinete development
NHS ester
DNA
α/β-tubulin
AKiT5
α/β-tubulin
AKiT5
4-8h
kinetochore capture
4-8h
biorientation
8-12h
anaphase
8-12h
anaphase
8-12h
nucleus migration
24h
4KT
B
NHS ester
DNA
α/β-tubulin
AKiT5
α/β-tubulin
AKiT5
4-8h
kinetochore capture, 3MTOC
8-12h
multipolar spindle

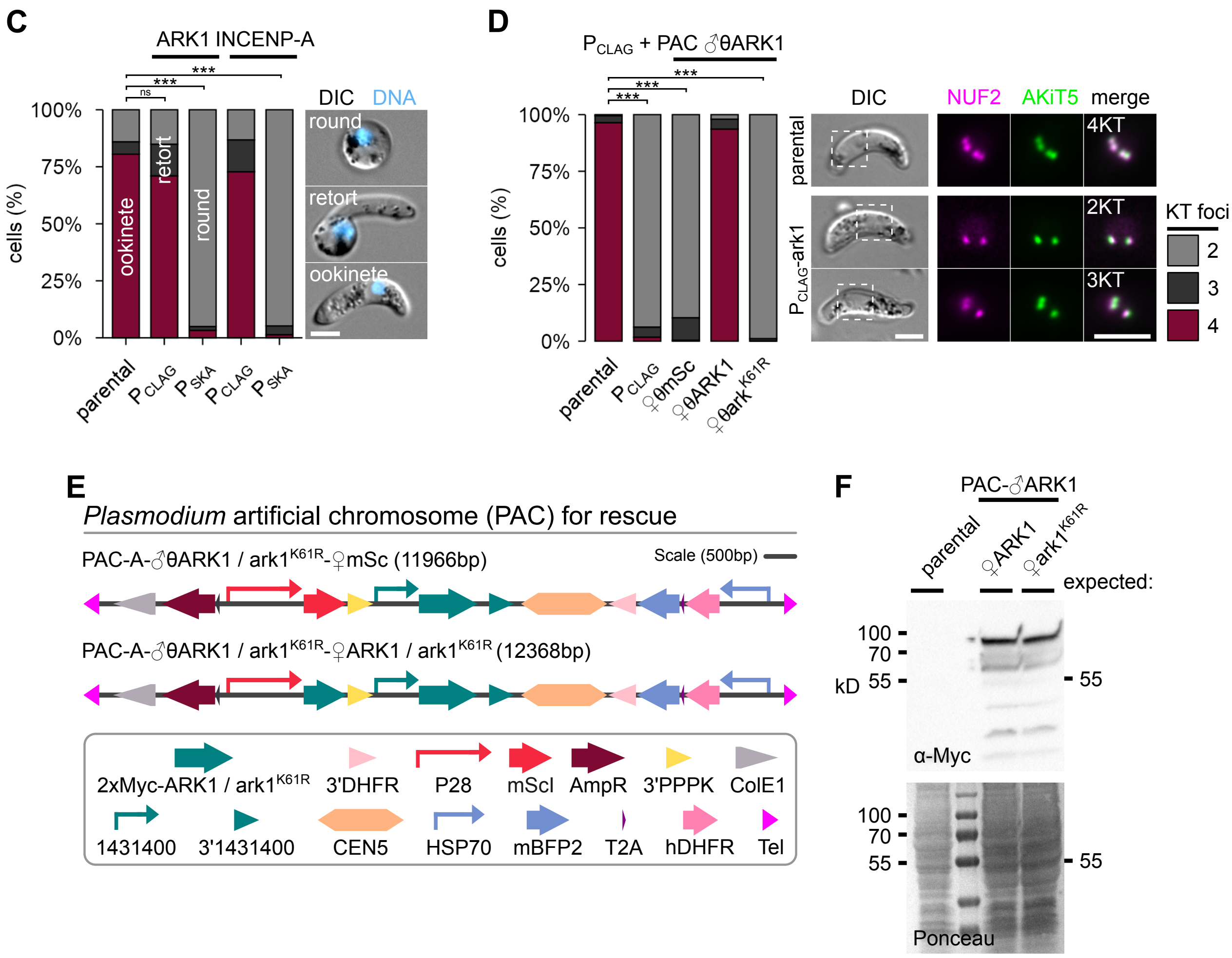
C
ARK1 INCENP-A
ns
***
***
100%
75%
50%
25%
0%
cells (%)
ookinete
retort
round
DIC DNA
round
retort
ookinete
parental
P CLAG
P SKA
P CLAG
P SKA
D
P CLAG + PAC ♂θARK1
***
***
***
100%
75%
50%
25%
0%
cells (%)
parental
P CLAG
♀θmSc
♀θARK1
♀θark K61R
DIC
NUF2
AKiT5
merge
parental
P CLAG-ark1
4KT
2KT
3KT
KT foci
2
3
4
E
Plasmodium artificial chromosome (PAC) for rescue
PAC-A-♂θARK1 / ark1 K61R-♀mSc (11966bp)
Scale (500bp)
PAC-A-♂θARK1 / ark1 K61R-♀ARK1 / ark1 K61R (12368bp)
2xMyc-ARK1 / ark1 K61R
3'DHFR
P28
mScI
AmpR
3'PPPK
ColE1
1431400
3'1431400
CEN5
HSP70
mBFP2
T2A
hDHFR
Tel
F
PAC-♂ARK1
parental
♀ARK1
♀ark1 K61R
expected:
100
70
kD 55
55
α-Myc
100
70
55
55
Ponceau

Figure S5. Expanded view of meiotic spindle and kinetochore dynamics and genetic rescue of ARK1 function during ookinete development. (A) U-ExM examples of parental ookinetes at the indicated stages, corresponding to Fig. 6A, displayed with single channels for NHS-ester, DNA, α/β-tubulin and AKiT5. Time points illustrate kinetochore capture, biorientation, anaphase-like configurations, nucleus migration and four kinetochore clusters in mature ookinetes. Boxed regions indicate merged zooms at right. Scale bar, 1 µm; dimensions are non-expanded. (B) Additional U-ExM examples of atypical meiotic spindles, including kinetochore capture in cells with three MTOCs and multipolar spindle configurations. Representative U-ExM phenotypes in A and B were observed across three independent biological replicates. (C) Morphological scoring of developing zygotes/ookinetes following ARK1 or INCENP-A depletion under PCLAG or PSKA regulation. Stacked bars show the proportion of round, retort and mature ookinete forms; each cell is the unit of analysis. (D) Quantification of kinetochore (KT) foci in mature ookinetes using NUF2 and AKiT5 in parental and PCLAG-ark1 parasites, and following PAC-mediated expression of ARK1 or ark1K61R; each ookinete is the unit of analysis. For C and D, n values are provided in the Source Data file and data were collected from six and three independent biological replicates, respectively. Statistical significance was assessed using chi-squared tests with no adjustment for multiple comparisons; exact P values are provided in the Source Data file. (E) Schematic of PAC constructs used for rescue experiments, including male- and female/zygote-stage ARK1 or ark1^{K61R} expression cassettes. (F) Anti-Myc immunoblot validating PAC-mediated expression of 2xMyc-tagged ARK1 or ark1^{K61R}; Ponceau S staining shows protein loading. Source data and uncropped blot images are provided as Source Data files.

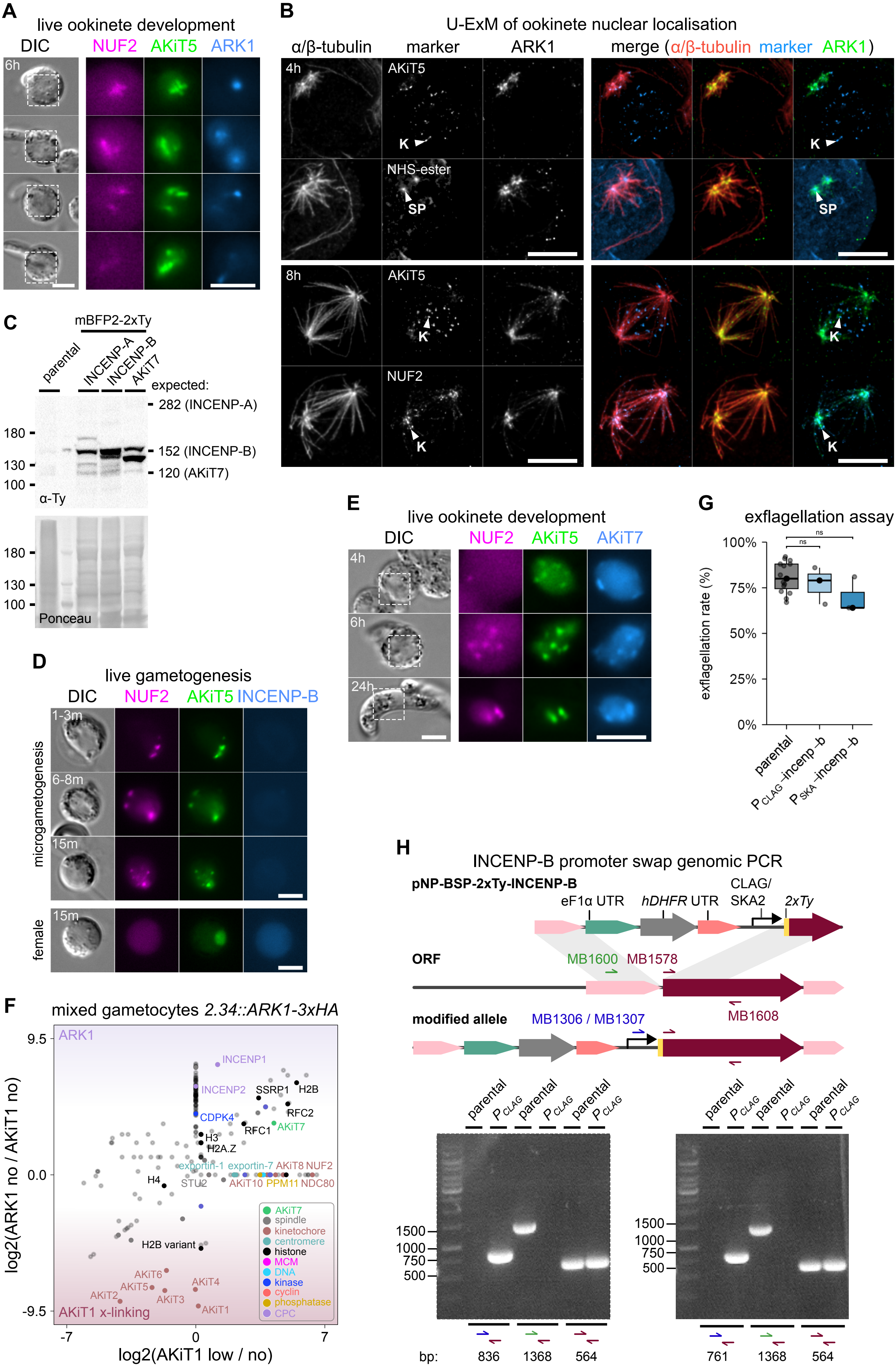

A
live ookinete development
DIC
NUF2
AKiT5
ARK1
6h
B
U-ExM of ookinete nuclear localisation
α/β-tubulin
marker
ARK1
merge (α/β-tubulin marker ARK1)
4h
AKiT5
K
NHS-ester
SP
8h
AKiT5
K
NUF2
K
C
mBFP2-2xTy
parental
INCENP-A
INCENP-B
AKiT7
expected:
282 (INCENP-A)
180
152 (INCENP-B)
130
120 (AKiT7)
100
α-Ty
180
130
100
Ponceau
D
live gametogenesis
DIC
NUF2
AKiT5
INCENP-B
1-3m
6-8m
15m
microgametogenesis
15m
female
E
live ookinete development
DIC
NUF2
AKiT5
AKiT7
4h
6h
24h
G
exflagellation assay
ns
ns
100%
75%
50%
25%
0%
exflagellation rate (%)
parental
P CLAG-incenp-b
P SKA-incenp-b
F
mixed gametocytes 2.34::ARK1-3xHA
9.5
0.0
-9.5
ARK1
INCENP1
INCENP2
SSRP1
H2B
CDPK4
RFC2
RFC1
AKiT7
H3
H2A.Z
exportin-1
exportin-7
AKiT8
NUF2
H4
STU2
AKiT10
PPM11
NDC80
H2B variant
AKiT6
AKiT5
AKiT4
AKiT2
AKiT3
AKiT1
AKiT1 x-linking
AKiT7
spindle
kinetochore
centromere
histone
MCM
DNA
kinase
cyclin
phosphatase
CPC
log2(ARK1 no / AKiT1 no)
-7
0
7
log2(AKiT1 low / no)
H
INCENP-B promoter swap genomic PCR
pNP-BSP-2xTy-INCENP-B
eF1α UTR
hDHFR UTR
CLAG/
SKA2
2xTy
ORF
MB1600
MB1578
MB1608
modified allele
MB1306 / MB1307
parental
P CLAG
parental
P CLAG
parental
P CLAG
1500
1000
750
500
bp:
836
1368
564
761
1368
564

Figure S6. Localisation and validation of CPC-associated factors and INCENP-B depletion lines. (A) Live widefield imaging of ookinete development at 6 h post-fertilisation showing cell-to-cell variability in NUF2-mScI signal, alongside AKiT5-mNG-3xHA and ARK1-mBFP2-2xTy. Boxed regions highlight nuclei. Scale bar, 2 μm. (B) U-ExM of developing zygotes/ookinetes at 4 h and 8 h post-fertilisation showing ARK1 relative to α/β-tubulin-labelled spindle microtubules and the indicated markers. Arrowheads indicate kinetochores (K) and spindle-pole regions (SP). Scale bars, 1 μm; dimensions are non-expanded. (C) Anti-Ty immunoblots of parasites expressing mBFP2-2xTy fusions for INCENP-A, INCENP-B and AKiT7; Ponceau S staining shows protein loading. (D) Live widefield imaging of INCENP-B-mBFP2-2xTy during male microgametogenesis and in female gametocytes, with NUF2-mScI and AKiT5-mNG-3xHA. Scale bars, 2 μm. (E) Live widefield imaging of AKiT7-mBFP2-2xTy during ookinete development, with NUF2-mScI and AKiT5-mNG-3xHA. Scale bars, 2 μm. Representative localisation patterns in A, B, D and E were reproduced in four independent biological replicates. (F) ARK1-3xHA interactome from mixed gametocytes at 6 min post-activation, highlighting CPC- and SAC-related factors. (G) Exflagellation assay of INCENP-B-depleted microgametocytes. n values are provided in the Source Data file; each mouse-derived parasite preparation is the unit of analysis. Statistical significance was assessed using two-sided Wilcoxon rank-sum tests with no adjustment for multiple comparisons; exact P values are provided in the Source Data file. (H) INCENP-B promoter-swap strategy and diagnostic PCR confirming genomic modification. Source data and uncropped blot/gel images are provided as Source Data files.

A

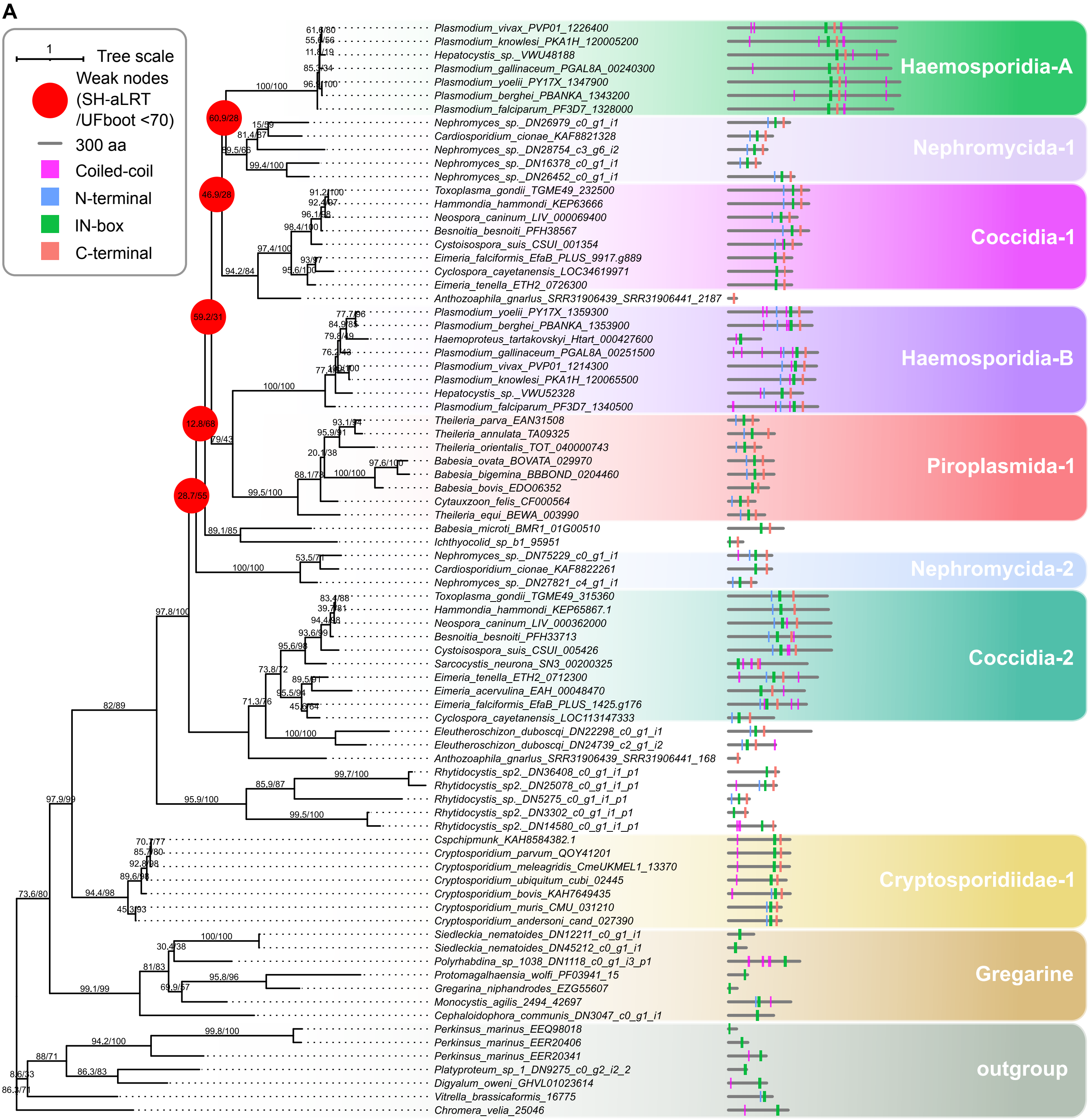
1
Tree scale
Weak nodes (SH-aLRT /UFboot <70)
300 aa
Coiled-coil
N-terminal
IN-box
C-terminal
Plasmodium_vivax_PVP01_1226400
Plasmodium_knowlesi_PKA1H_120005200
Hepatocystis_sp._VWU48188
Plasmodium_gallinaceum_PGAL8A_00240300
Plasmodium_yoelii_PY17X_1347900
Plasmodium_berghei_PBANKA_1343200
Plasmodium_falciparum_PF3D7_1328000
Haemosporidia-A
Nephromyces_sp._DN26979_c0_g1_i1
Cardiosporidium_cionae_KAF8821328
Nephromyces_sp._DN28754_c3_g6_i2
Nephromyces_sp._DN16378_c0_g1_i1
Nephromyces_sp._DN26452_c0_g1_i1
Nephromycida-1
Toxoplasma_gondii_TGME49_232500
Hammondia_hammondi_KEP63666
Neospora_caninum_LIV_000069400
Besnoitia_besnoiti_PFH38567
Cystoisospora_suis_CSUI_001354
Eimeria_falciformis_EfaB_PLUS_9917.g889
Cyclospora_cayetanensis_LOC34619971
Eimeria_tenella_ETH2_0726300
Anthozoaphila_gnarlus_SRR31906439_SRR31906441_2187
Coccidia-1
Plasmodium_yoelii_PY17X_1359300
Plasmodium_berghei_PBANKA_1353900
Haemoproteus_tartakovskyi_Htart_000427600
Plasmodium_gallinaceum_PGAL8A_00251500
Plasmodium_vivax_PVP01_1214300
Plasmodium_knowlesi_PKA1H_120065500
Hepatocystis_sp._VWU52328
Plasmodium_falciparum_PF3D7_1340500
Haemosporidia-B
Theileria_parva_EAN31508
Theileria_annulata_TA09325
Theileria_orientalis_TOT_040000743
Babesia_ovata_BOVATA_029970
Babesia_bigemina_BBBOND_0204460
Babesia_bovis_EDO06352
Cytauxzoon_felis_CF000564
Theileria_equi_BEWA_003990
Piroplasmida-1
Babesia_microti_BMR1_01G00510
Ichthyocolid_sp_b1_95951
Nephromyces_sp._DN75229_c0_g1_i1
Cardiosporidium_cionae_KAF8822261
Nephromyces_sp._DN27821_c4_g1_i1
Nephromycida-2
Toxoplasma_gondii_TGME49_315360
Hammondia_hammondi_KEP65867.1
Neospora_caninum_LIV_000362000
Besnoitia_besnoiti_PFH33713
Cystoisospora_suis_CSUI_005426
Sarcocystis_neurona_SN3_00200325
Eimeria_tenella_ETH2_0712300
Eimeria_acervulina_EAH_00048470
Eimeria_falciformis_EfaB_PLUS_1425.g176
Cyclospora_cayetanensis_LOC113147333
Coccidia-2
Eleutheroschizon_duboscqi_DN22298_c0_g1_i1
Eleutheroschizon_duboscqi_DN24739_c2_g1_i2
Anthozoaphila_gnarlus_SRR31906439_SRR31906441_168
Rhytidocystis_sp2._DN36408_c0_g1_i1_p1
Rhytidocystis_sp2._DN25078_c0_g1_i1_p1
Rhytidocystis_sp._DN5275_c0_g1_i1_p1
Rhytidocystis_sp2._DN3302_c0_g1_i1_p1
Rhytidocystis_sp2._DN14580_c0_g1_i1_p1
Cspchipmunk_KAH8584382.1
Cryptosporidium_parvum_QOY41201
Cryptosporidium_meleagridis_CmeUKMEL1_13370
Cryptosporidium_ubiquitum_cubi_02445
Cryptosporidium_bovis_KAH7649435
Cryptosporidium_muris_CMU_031210
Cryptosporidium_andersoni_cand_027390
Cryptosporidiidae-1
Siedleckia_nematoides_DN12211_c0_g1_i1
Siedleckia_nematoides_DN45212_c0_g1_i1
Polyrhabdina_sp_1038_DN1118_c0_g1_i3_p1
Protomagalhaensia_wolfi_PF03941_15
Gregarina_niphandrodes_EZG55607
Monocystis_agilis_2494_42697
Cephaloidophora_communis_DN3047_c0_g1_i1
Gregarine
Perkinsus_marinus_EEQ98018
Perkinsus_marinus_EER20406
Perkinsus_marinus_EER20341
Platyproteum_sp_1_DN9275_c0_g2_i2_2
Digyalum_oweni_GHVL01023614
Vitrella_brassicaformis_16775
Chromera_velia_25046
outgroup

Figure S7. INCENP phylogeny and domain architecture across apicomplexans and related alveolates. (A) Maximum-likelihood phylogeny of conserved INCENP regions from apicomplexans and selected alveolate outgroups. Major lineage groups are shaded. Red circles mark weak deeper nodes with SH-aLRT and/or ultrafast bootstrap support <70. Horizontal grey bars indicate protein length; coloured ticks indicate manually curated domains or motifs detected with custom HMMs; magenta segments indicate coiled-coil regions predicted with ncoils. The tree recovers lineage-level clades, including haemosporidian, coccidian, nephromycid, piroplasmid and cryptosporidiid groupings, but retains weak deep-backbone support, consistent with the limited ability of full-length INCENP phylogeny alone to resolve duplication timing.

Table S1. Oligonucleotides and genetic constructs used in this study.

Sequences and descriptions of primers for tagging, UTR exchange, and integration, including plasmid backbones and targeted genes.

modification	base plasmid	gene	gene name	oligo ID
Tagging at the C terminus	pCPY	PBANKA_0104100	*ARK1*	MB1485
		PBANKA_0104100	*ARK1*	MB1486
		PBANKA_0104100	*ARK1*	MB1487
		PBANKA_0104100	*ARK1*	MB1488
		PBANKA_0104100	*ARK1*	MB1664
		PBANKA_1343200	*INCENP1*	MB1036
		PBANKA_1343200	*INCENP1*	MB1037
		PBANKA_1343200	*INCENP1*	MB1038
		PBANKA_1343200	*INCENP1*	MB1039
		PBANKA_1343200	*INCENP1*	MB1665
		PBANKA_1353900	*INCENP2*	MB1526
		PBANKA_1353900	*INCENP2*	MB1527
		PBANKA_1353900	*INCENP2*	MB1528
		PBANKA_1353900	*INCENP2*	MB1529
		PBANKA_1353900	*INCENP2*	MB1530
		PBANKA_0612300	*AKiT7/MAD1*	MB1337
		PBANKA_0612300	*AKiT7/MAD1*	MB1338
		PBANKA_0612300	*AKiT7/MAD1*	MB1339
		PBANKA_0612300	*AKiT7/MAD1*	MB1340
		PBANKA_0612300	*AKiT7/MAD1*	MB1327
		mBFP2	*mBFP2*	MB1626
Replacement of the 5'UTR	pNP	PBANKA_0104100	*ARK1*	MB1249
		PBANKA_0104100	*ARK1*	MB1250
		PBANKA_0104100	*ARK1*	MB1251
		PBANKA_0104100	*ARK1*	MB1252
		PBANKA_0104100	*ARK1*	MB1258
		PBANKA_1343200	*INCENP1*	MB1051
		PBANKA_1343200	*INCENP1*	MB1052
		PBANKA_1343200	*INCENP1*	MB1053
		PBANKA_1343200	*INCENP1*	MB1054
		PBANKA_1343200	*INCENP1*	MB1075
		PBANKA_1353900	*INCENP2*	MB1600
		PBANKA_1353900	*INCENP2*	MB1601
		PBANKA_1353900	*INCENP2*	MB1578
		PBANKA_1353900	*INCENP2*	MB1579
		PBANKA_1353900	*INCENP2*	MB1608
		PBANKA_1400600	*CLAG*	MB1306
		PBANKA_0405800	*SKA2*	MB1307
PAC	pBAT1_PB401	PbCEN5		MB1700
		PbCEN5		MB1701

sequence	description
tgGCGGCCGCAGAGGTACTCATGATTACTGGTC	pCPY-ARK1_CDS_F
tgGGGCCCTTAGTCTAACGGGTCCTGATTCGTATGCACTTCATCCAGTGGGTCTTGGTTTGTGTGAACC	
TCCCGCGGTTTATTATGAAACCAAATTGGAGTAAGAAAAGA	pCPY-ARK1_CDS_R
TGGGTACCATGCAAAAAATATTTATGGGTAAAAAGTATATATTTATTTGACAT	pCPY-ARK1_UTR_F
TGGCGGCCGCTGGAAACTTATTAATTTTTTATTTTTTCCAAAATTGAATTACTG	pCPY-ARK1_UTR_R
GGATTATCTGCACATATAGGATCTAAACA	pCPY-ARK1_INT
CTGGTACCACGAGATCTAAAAATATACAAATAAATATAATTCTAGATATCCCCTTATACT	pCPY-INCENP1_UTR_F
CTGCGGCCGCAAAAATATATCATACACTTAGTGCAATCCT	pCPY-INCENP1_UTR_R
CTGCGGCCGCAGAGACTAACATTCTAAAATTAACACAAGA	pCPY-INCENP1_CDS_F
CTCCTAGGGAATAATTTTTTAAAAACAGAAGTAGCCATTTTAAA	pCPY-INCENP1_CDS_R
CTGGTAATAGTAGTAATAATACCCTGGA	pCPY-INCENP1_INT
TGGCGGCCGCGTATGAAAATATATACCTATGAAAAATGCTTTTGC	pCPY-INCENP2_UTR_F
TGCCGCGGCTCGAGTATTATCGAATTGCACTTTTTACTCCTCT	pCPY-INCENP2_UTR_R
TGGGTACCATACACCTTTTTTGGTATAGTTATGATTATTGTATTTTT	pCPY-INCENP2_CDS_F
TGGCGGCCGCGAACGTTCATACAATTTCTATAGACTTATTCG	pCPY-INCENP2_CDS_R
GAATGCAATATGTTCTTAGATCCTCTAC	pCPY-INCENP2_INT
TGGGTACCAAACTTGAAAAATACATGTCTTAATATTTTCATTTACCA	pCPY-AKiT7_UTR_F
TGGCGGCCGCTCACATTTAGCACAAATTATGATCCTCTAAT	pCPY-AKiT7_UTR_R
TGGCGGCCGCAATGCAGAATGACAAACGATCATATAATAAAG	pCPY-AKiT7_CDS_F
TGCTCGAGTATTTTGAAAAGTATACTCCTTGAGTTTTTGC	pCPY-AKiT7_CDS_R
GGGAAACCTCAAGATGTAATAAATAAACAG	pCPY-AKiT7_INT
GCACTTGAAGTGATGGTTGT	pCPY-mBFP2_INT
TGGCGGCCGCATCCAAATTTACATATACATATTTCTTTTACTTTATTTTCTTACG	pNP-ARK1_UTR_F
TGGAATTCTTTTTCGAAGAATGTGTGCATATATGTAAA	pNP-ARK1_UTR_R
TGGGGCCCAACGAATCAGCTAGTAGTCCA	pNP-ARK1_CDS_F
TGGCGGCCGCTACTTGTATGGAACCACGTATACA	pNP-ARK1_CDS_R
CCCAAATTATTTCGAATAACATAGTTGC	pNP-ARK1_INT
TGGGGCCCCTTGTTGTAACAGAAAGCACACTT	pNP-INCENP1_CDS_F
TGGCGGCCGCAACCTTTCTTTGACTTTTTATTAGGATGTTTATGT	pNP-INCENP1_CDS_R
TGGCGGCCGCACAAAGCATACATAGAGAAATACAAATAGTAAAAGG	pNP-INCENP1_UTR_F
TGGAATTCGATGCACATAATTAATTTTATTAATGAAAAATGTGTTGTTT	pNP-INCENP1_UTR_R
GATGTGTTTGACTTTCTACTTGTAACATTT	pNP-INCENP1_INT
TGGCGGCCGCGAACAAGAAACAGACATGTTTGAATGA	pNP-INCENP2_UTR_F
TGGAATTCGTATATATGCTTACCCCAAC	pNP-INCENP2_UTR_R
TGGGGCCCAATACTTTTGTGGAAAATGGTGATATAAATAAAAATGA	pNP-INCENP2_CDS_F
TGGCGGCCGCTTTTTGTATAATCCCATATATAACAGAATTTTGTTTTATTGC	pNP-INCENP2_CDS_R
GTTAATCTGATTAGGATGTGGATTGTTAGA	pNP-INCENP2_INT
cattcttaacaaaattaataatgcaaagccc	pNP-CLAG_INT
GATCAAAGTTGGAACATATATATTGCCT	pNP-SKA_INT
tgGATATCtgatgggttacattttgtaagaaataagaaaaT	PbCEN5_F
tgGAGCTCtttaaGGTATTTGTATCATAATCATTTTGTTTGTCT	PbCEN5_R

Table S2. Antibodies and stains used in this study.

Details of antibodies, fluorescent dyes, and stains including source, dilution, application (IFA, U-ExM, Western blot), and lot numbers.

antibody/stain	raised in	reference	source	dilution			lot/batch	other lot/batch
				IFA	U-ExM	western		
SYTOX Deep Red Nucleic Acid Stain		S11381	Invitrogen		1/1600		2916338	
Hoechst 33342, trihydrochloride, trihydrate		H3570	Invitrogen		1/400		2831276	
Alexa Fluor™ 405 NHS Ester (Succinimidyl Ester)		A30000	Invitrogen		1/250		2884573	
DyLight™ 488 NHS Ester		46402	Invitrogen		1/250		ZE4298191	
mCLING		710 006AT647N	SYSY		1/500			
α-alpha-tubulin	guinea pig	ABCD_AA345	Unige antibody facility		1/320		45258	AA345-Gp
α-beta-tubulin	guinea pig	ABCD_AA344	Unige antibody facility		1/343		45258	AA344-Gp
α-HA (3F10)	rat	11867431001	Roche		1/666	1/1000	66109200	75279200
α-Rabbit IgG (H+L) Alexa Fluor™ 568	goat	A11011	Invitrogen		1/1000		2782620	
α-Guinea Pig IgG (H+L) Alexa Fluor™ 568	goat	A11075	Invitrogen		1/1000		2872022	
α-Rat IgG (H+L) Alexa Fluor™ 488	goat	A11006	Invitrogen		1/1000		2747446	
α-Guinea Pig IgG (H+L) Alexa Fluor™ 647	goat	A21450	Invitrogen		1/1000		2795342	
α-Ty	rabbit	Uboldi et al. (2015). Cell Host Microbe. Dec 9;18(6):670-81	Chris Tonkin (WEHI)		1/500			
α-Ty	mouse	clone BB2	In house hybridoma		1/500	1/5		
α-Myc	mouse	In house hybridoma				1/10		
α-Strep-tag II	rabbit	ab76949	abcam			1/2000 - 1/1000	1101072-11	
α-rabbit IgG - HRP	goat	A8275	Sigma-Aldrich			1/3000		
α-mouse IgG - HRP	goat	A5278	Sigma-Aldrich			1/3000		
α-RFP	rat	5F8-20	Chromotek		1/500		D0000062	
α-RFP	rabbit	600-401-379S	Rockland		1/500		48776	
