# Supplementary material for: A modular chromosomal passenger complex rewires chromosome segregation in Plasmodium berghei
Source: Nat Commun. 2026 Jun 20;17:7788. doi: 10.1038/s41467-026-74542-7 (PMC13439184; doi:10.1038/s41467-026-74542-7)
Supplement: Supplementary file 2 [file 41467_2026_74542_MOESM23_ESM.pdf]

nature portfolio

Corresponding author(s): Lorenzo Brusini

Last updated by author(s): 05.05.26

Reporting Summary

Nature Portfolio wishes to improve the reproducibility of the work that we publish. This form provides structure for consistency and transparency in reporting. For further information on Nature Portfolio policies, see our Editorial Policies and the Editorial Policy Checklist.

Statistics

For all statistical analyses, confirm that the following items are present in the figure legend, table legend, main text, or Methods section.

n/a	Confirmed	
☐	☒	The exact sample size (*n*) for each experimental group/condition, given as a discrete number and unit of measurement
☐	☒	A statement on whether measurements were taken from distinct samples or whether the same sample was measured repeatedly
☐	☒	The statistical test(s) used AND whether they are one- or two-sided<br>*Only common tests should be described solely by name; describe more complex techniques in the Methods section.*
☒	☐	A description of all covariates tested
☐	☒	A description of any assumptions or corrections, such as tests of normality and adjustment for multiple comparisons
☐	☒	A full description of the statistical parameters including central tendency (e.g. means) or other basic estimates (e.g. regression coefficient) AND variation (e.g. standard deviation) or associated estimates of uncertainty (e.g. confidence intervals)
☐	☒	For null hypothesis testing, the test statistic (e.g. *F*, *t*, *r*) with confidence intervals, effect sizes, degrees of freedom and *P* value noted<br>*Give P values as exact values whenever suitable.*
☒	☐	For Bayesian analysis, information on the choice of priors and Markov chain Monte Carlo settings
☒	☐	For hierarchical and complex designs, identification of the appropriate level for tests and full reporting of outcomes
☒	☐	Estimates of effect sizes (e.g. Cohen's *d*, Pearson's *r*), indicating how they were calculated

Our web collection on statistics for biologists contains articles on many of the points above.

Software and code

Policy information about availability of computer code

Data collection	Data were collected using Summit v6.3.1 on a Beckman Coulter MoFlo Astrios EQ cell sorter, BD FACSChorus v6.2.8 on a BD FACSDiscover S8 Cell Sorter with CellView imaging technology, ZEN 3.11, Leica Application Suite X, Leica TCS SP8, Leica Stellaris 8 FALCON, Leica DM5500, Proteome Discoverer v2.3 and Mascot 3.x. Kinetochore, spindle and protein architecture schematics were generated in Inkscape 1.4.2.
Data analysis	Data were analysed using FlowJo v11, BD FACSChorus v6.2.8, Fiji/ImageJ v2.17.0, Imaris v9.5, Leica Lightning v4.1.1, Huygens Essential v25.10, Proteome Discoverer v2.3, Mascot 3.x, MAFFT 7.526, IQ-TREE 3.1.1, HMMER 3.4, HHsearch/HHsuite 3.3.0, ncoils/COILS, and R 4.6.0 with ggplot2. No custom software or mathematical algorithm central to the conclusions was developed.

For manuscripts utilizing custom algorithms or software that are central to the research but not yet described in published literature, software must be made available to editors and reviewers. We strongly encourage code deposition in a community repository (e.g. GitHub). See the Nature Portfolio guidelines for submitting code & software for further information.

Data

Policy information about availability of data

All manuscripts must include a data availability statement. This statement should provide the following information, where applicable:

- Accession codes, unique identifiers, or web links for publicly available datasets
- A description of any restrictions on data availability
- For clinical datasets or third party data, please ensure that the statement adheres to our policy

All data supporting the findings of this study are available within the paper, the Supplementary Information, the Source Data files, the Supplementary Data files, and

associated public repositories. Source Data are provided with this paper. Proteomics mass spectrometry data generated in this study have been deposited in the PRIDE database under accession code PXD068832 [https://proteomecentral.proteomexchange.org/cgi/GetDataset?ID=PXD068832]. The dataset is currently available to editors and reviewers through PRIDE reviewer access and will be made publicly available upon publication. Supplementary Data 1–9 contain quantitative measurements, raw counts, interactome outputs, comparative sequence datasets, and related source datasets. Supplementary Data 10–12 contain full U-ExM 3D reconstructions and additional representative imaging datasets.

Research involving human participants, their data, or biological material

Policy information about studies with human participants or human data. See also policy information about sex, gender (identity/presentation), and sexual orientation and race, ethnicity and racism.

Reporting on sex and gender	N/A
Reporting on race, ethnicity, or other socially relevant groupings	N/A
Population characteristics	N/A
Recruitment	N/A
Ethics oversight	N/A

Note that full information on the approval of the study protocol must also be provided in the manuscript.

Field-specific reporting

Please select the one below that is the best fit for your research. If you are not sure, read the appropriate sections before making your selection.

☒ Life sciences ☐ Behavioural & social sciences ☐ Ecological, evolutionary & environmental sciences

For a reference copy of the document with all sections, see nature.com/documents/nr-reporting-summary-flat.pdf

Life sciences study design

All studies must disclose on these points even when the disclosure is negative.

Sample size	No statistical method was used to predetermine sample size. Sample sizes were chosen based on previous work in P. berghei sexual-stage imaging, mosquito-transmission and U-ExM assays, and on the number of biological replicates, mosquito midguts, oocysts, pooled sporozoite samples or fully reconstructed cells required to capture reproducible stage-specific phenotypes. Biological replicate numbers, sample sizes, units of analysis and statistical tests are provided in the figure legends and Source Data file.
Data exclusions	No data were excluded from the analyses unless explicitly stated. All analysed data underlying the figures are provided in the Source Data and Supplementary Data files.
Replication	Representative imaging experiments were repeated independently with similar results as stated in the relevant figure legends. Biological replicate numbers are provided in the figure legends and Source Data file. No findings were irreproducible.
Randomization	Samples were allocated to experimental groups according to parasite genotype, reporter configuration, developmental stage and time point. Random allocation was not applicable because groups were defined by engineered parasite lines, matched parental controls or defined developmental stages.
Blinding	Blinding was not used for most experiments because experimental groups were identifiable from genotype, fluorescent reporter configuration or developmental phenotype during acquisition and analysis. Where flow-cytometry gates, imaging-flow gates, segmentation thresholds or quantitative image-analysis settings were used, the same analysis criteria were applied consistently across compared groups.

Reporting for specific materials, systems and methods

We require information from authors about some types of materials, experimental systems and methods used in many studies. Here, indicate whether each material, system or method listed is relevant to your study. If you are not sure if a list item applies to your research, read the appropriate section before selecting a response.

Materials & experimental systems

n/a	Involved in the study
	[x] Antibodies
	[x] Eukaryotic cell lines
[x]	Palaeontology and archaeology
	[x] Animals and other organisms
[x]	Clinical data
[x]	Dual use research of concern
[x]	Plants

Methods

n/a	Involved in the study
[x]	ChIP-seq
	[x] Flow cytometry
[x]	MRI-based neuroimaging

Antibodies

Antibodies used	Antibodies, stains, suppliers, catalogue or reference information, lot numbers where available, applications and dilutions are listed in Supplementary Table S11.
Validation	Primary antibodies were validated by expected localisation patterns, absence of signal in untagged parental controls where applicable, detection of tagged proteins at expected molecular weights where technically possible, and/or previous literature or manufacturer validation as indicated in Supplementary Table S11 and the Methods.

Eukaryotic cell lines

Policy information about cell lines and Sex and Gender in Research

Cell line source(s)	Plasmodium berghei ANKA clone 2.34 and derived transgenic parasite lines were used. The male-only fd1− and female-only md4− parasite lines were obtained from previously published work as cited in the manuscript.
Authentication	Parasite identities and genotypes were confirmed by diagnostic PCR, fluorescence phenotyping, immunoblotting where applicable, and/or expected stage-specific phenotypes as described in the Methods and Source Data files.
Mycoplasma contamination	N/A
Commonly misidentified lines (See ICLAC register)	N/A

Animals and other research organisms

Policy information about studies involving animals; ARRIVE guidelines recommended for reporting animal research, and Sex and Gender in Research

Laboratory animals	Mus musculus CD1 outbred or BALB/c female mice, 6–8 weeks old, were obtained from Charles River Laboratories and used as hosts for Plasmodium berghei parasite propagation, mosquito infection and transmission assays. Mice were housed under specific-pathogen-free conditions in individually ventilated cages at 21 ± 2°C, with humidity monitored according to institutional SPF facility standards, under a 12 h light/dark cycle, with autoclaved chow and water ad libitum. Anopheles stephensi mosquitoes were maintained at 28°C, 80% relative humidity and a 12 h light/dark cycle, and after infectious blood meals were maintained at 20°C.
Wild animals	N/A
Reporting on sex	Female mice were used for parasite propagation, mosquito infection and transmission assays. Sex was not analysed as an experimental variable because mice served as hosts for parasite development and mosquito feeding rather than as the biological subject of the chromosome-segregation phenotypes measured.
Field-collected samples	N/A. Parasite transmission was studied using laboratory-reared Anopheles stephensi mosquitoes.
Ethics oversight	All animal experiments were approved by the competent cantonal veterinary authorities under licence numbers GE283C (Canton of Geneva) and BE118/22 (Canton of Bern). For the Geneva licence, applications were evaluated by the Commission Cantonale pour les Expériences sur les Animaux and authorised by the Service de la consommation et des affaires vétérinaires. Experiments were performed in accordance with Swiss Federal Food Safety and Veterinary Office guidelines and the Swiss Animal Welfare Act.

Note that full information on the approval of the study protocol must also be provided in the manuscript.

Plants

Seed stocks	N/A
Novel plant genotypes	N/A
Authentication	N/A

Flow Cytometry

Plots

Confirm that:

- [x] The axis labels state the marker and fluorochrome used (e.g. CD4-FITC).
- [x] The axis scales are clearly visible. Include numbers along axes only for bottom left plot of group (a 'group' is an analysis of identical markers).
- [x] All plots are contour plots with outliers or pseudocolor plots.
- [x] A numerical value for number of cells or percentage (with statistics) is provided.

Methodology

Sample preparation	Flow cytometry was used to analyse and sort Plasmodium berghei blood-stage parasites. Tail-blood samples were diluted in PBS as described in the Methods. Imaging-flow cytometry was used to analyse enriched mature ookinetes 48 h after crossing, following Histodenz enrichment, ammonium chloride treatment to lyse residual erythrocytes, washing and resuspension in ookinete medium.
Instrument	Conventional flow cytometry was performed on a Beckman Coulter MoFlo Astrios EQ cell sorter. Imaging-flow cytometry was performed on a BD FACSDiscover S8 Cell Sorter with CellView imaging technology.
Software	Summit v6.3.1 was used for MoFlo Astrios EQ acquisition, FlowJo v11 for post-acquisition flow-cytometry analysis, and BD FACSChorus v6.2.8 for FACSDiscover S8 acquisition and on-the-fly imaging-flow cytometry analysis.
Cell population abundance	Blood-stage reporter retention was quantified as the percentage of GFP+BFP+ events among total GFP+ singlets. Paternal inheritance after fertilisation was quantified as the proportion of GFP+ mature ookinetes that were mScl+ after imaging-flow cytometry gating.
Gating strategy	Sequential gating strategies are shown in Supplementary Fig. S2. For blood-stage flow cytometry, events were gated by forward- and side-scatter properties, followed by singlet and doublet-exclusion gates, and then GFP/BFP fluorescence to identify reporter-retaining parasites. For imaging-flow cytometry, events were gated by light-loss and SSC parameters, singlet/morphology gates, GFP and mScl fluorescence, and mature ookinete morphology using eccentricity and long-axis moment. Supplementary Fig. S2A corresponds to Fig. 2C and the blood-stage component of Fig. 2D; Supplementary Fig. S2B corresponds to mature ookinete identification and paternal inheritance scoring in Fig. 2D.

- [x] Tick this box to confirm that a figure exemplifying the gating strategy is provided in the Supplementary Information.
