# Supplementary material for: A modular chromosomal passenger complex rewires chromosome segregation in Plasmodium berghei
Source: Nat Commun. 2026 Jun 20;17:7788. doi: 10.1038/s41467-026-74542-7 (PMC13439184; doi:10.1038/s41467-026-74542-7)
Supplement: Supplementary file 3 [file 41467_2026_74542_MOESM24_ESM.pdf]

nature portfolio

Peer Review File

A Modular Chromosomal Passenger Complex Rewires Chromosome Segregation in Plasmodium berghei

Corresponding Author: Dr Lorenzo Brusini

This file contains all reviewer reports in order by version, followed by all author rebuttals in order by version.

Version 0:

Reviewer comments:

Reviewer #1

(Remarks to the Author)
Summary: This study investigates how Aurora-related kinase 1 (ARK1) coordinates chromosome segregation during Plasmodium berghei sexual development. Using proximity-labeling proteomics (BioID-like), imaging, and functional assays, the authors suggest that Plasmodium's chromosomal passenger complex (CPC) acts as a stage-specific modular system, featuring a minimal ARK1–INCENP1 module in male gametogony and an expanded CPC that includes kinetochore components during meiosis. The work offers valuable mechanistic insights into how this parasite adapts its mitotic and meiotic machinery to its unique life cycle.
The dataset is impressive and technically advanced, and the study is likely to become a valuable resource for the community. However, several claims are overstated, and some key recent literature, particularly the studies by Li et al. (2024, PMID: 38987258) and Nagar et al. (2025, PMID: 40909691), should be recognized to contextualize this work appropriately.
Major Comments
1. Conceptual framing and overstatements
a) Scope of "rewiring" claims:
The manuscript often states that Plasmodium "rewires eukaryotic checkpoint control." These conclusions mainly come from P. berghei sexual stages and do not include recent structural and mechanistic data from asexual stages (Li et al. (2024, PMID: 38987258)) showing conservation of spindle–kinetochore–centrosome coupling. The language should be more cautious and based on proven differences rather than broad evolutionary generalizations.
The manuscript's claim of "rewiring of checkpoint control" in Plasmodium should be more cautiously framed as illustrating the plasticity and adaptation of cell division machinery within a limited phylogenetic and life stage context. Comprehensive comparative data, such as those provided by Nagar et al. (2025, PMID: 40909691), demonstrate divergent CPC architectures and checkpoint components across Apicomplexa, including losses and replacements in lineages like Cryptosporidium. Thus, descriptions of CPC "modularity" and stage-specific composition should be contextualized as possible lineage-specific innovations rather than broadly conserved eukaryotic features. Explicit acknowledgment of these contemporary works and phylogenetic nuance will improve accuracy and prevent overgeneralization.
b) Novelty of CPC modularity:
The characterization of the CPC's modularity as "previously unrecognized" is overstated. Nagar et al. (2025, PMID: 40909691) independently report ARK1's role in spindle/kinetochore regulation, the identification of two INCENPs, and the absence of Survivin/Borealin, demonstrating CPC divergence and stage-specific inclusion. The present study provides complementary spatial and functional insights, but it is not the first to recognize CPC modularity. It would strengthen the Discussion to acknowledge this concurrent work explicitly.
c) Generality across Apicomplexa:
The mechanistic data and evolutionary inferences in this study are solely based on experiments with Plasmodium berghei. Yet, the Discussion often generalizes these findings to "apicomplexan" and even broader "eukaryotic" principles. These broad generalizations should be tempered, as cell-division mechanisms, checkpoint control, and molecular structures vary significantly among different apicomplexan species, including Plasmodium falciparum, Toxoplasma gondii, Babesia spp., and especially Cryptosporidium, as well as among stages within a single species.
Recent comparative research by Nagar et al. (2025, PMID: 40909691) systematically examined ARK kinases and chromosomal passenger complex (CPC) components across the Apicomplexa. It revealed that, while Plasmodium and Toxoplasma share a diverged CPC lacking Survivin and Borealin, lineages like Cryptosporidium have lost or replaced many of the core CPC proteins entirely. These findings emphasize that claims about CPC modularity or structure should be

considered lineage-specific hypotheses rather than universal features of the phylum.
Including or referencing such cross-lineage comparative work would improve phylogenetic accuracy and strengthen the evolutionary Discussion, ensuring that conclusions based on P. berghei are contextualized adequately within the remarkable diversity and adaptability of apicomplexan cell-division strategies.

2. Integration with existing literature

• Li et al. (2024, PMID: 38987258):
This expansion microscopy study showed that losing kinetochore proteins PfNDC80 or PfNuf2 causes spindle–centriole-like plaque detachment and results in the formation of anucleate merozoites, revealing conserved mechanisms of kinetochore–MTOC anchoring during the asexual stages. This significant work should be cited when discussing the structural connection between the kinetochore and centrosomal counterparts.

• Nagar et al. (2025, PMID: 40909691):
This contemporaneous study provides a complementary ARK1 interactome obtained through GFP-based immunoprecipitation. It covers a broader range of life stages, including asexual schizogony, and offers detailed comparative genomics that reveal diversity in CPC composition across Apicomplexa. Including a brief acknowledgment of this work will help readers understand both studies within the context of ongoing Plasmodium cell division research.

3. Methodological distinctions and limitations

• Proximity labeling vs. immunoprecipitation:
The ARK1 interactome data in this manuscript are derived from proximity labeling combined with cross-linking, which identifies proteins in the spatial neighborhood of ARK1 rather than solely direct binders. This distinction should be explicitly emphasized, especially when interpreting the composition and structure of the chromosomal passenger complex. In comparison, Nagar et al. (2025, PMID 40909691) used direct co-immunoprecipitation coupled with mass spectrometry (IP-MS), which selectively enriches for stable and direct interactions. The authors might note that proximity labeling offers complementary insights by capturing transient or spatially restricted interactions, but it requires cautious interpretation to prevent overcalling stable complexes.

• Caveats on stage extrapolation:
The study's conclusions primarily rely on sexual-stage data, such as male gametogony and zygote/ookinete development. The manuscript should temper claims that these modular CPC mechanisms universally apply to all Plasmodium mitoses, including asexual blood stages, which feature distinct cell cycle characteristics and regulatory structures. Additional experimental validation in other stages is necessary before making broad generalizations.

4. Data presentation and figure clarity

• Figure formatting:
In Figure 3, axis labels overlap and truncate protein names. Adjust figure layout for legibility.
For Figure 1, add quantification of biological replicates (currently unclear).

5. Comparative and conceptual suggestions

• Clarify the differences between ARK1's proximity labeling interactome data (this study) and the direct immunoprecipitation-based interactome reported by Nagar et al. (2025, PMID 40909691), explicitly discussing which interactions may represent stable versus transient or spatially proximal associations. This distinction is important for understanding the composition and dynamics of the chromosomal passenger complex.
• Highlight that Nagar et al. (2025, PMID 40909691) also provide complementary transcriptomic (RNA-Seq) and imaging data that support broader functional roles of ARK1 across different life stages. These datasets should be acknowledged in the Discussion as supporting and expanding the mechanistic insights presented here.
• Recommend reframing any broad claims of "pan-apicomplexan" conservation or rewiring with specificity regarding the taxonomic lineages and life cycle stages where these findings have been validated. This will help readers clearly understand the evolutionary scope and limitations.
• Suggest including a graphical summary or model figure contrasting canonical, divergent, and modular architectures of the chromosomal passenger complex, integrating ARK1, INCENP paralogs, and kinetochore markers (e.g., AKiT proteins), to better visualize and contextualize the current findings within the broader field.

Overall assessment

This study offers a technically sound and visually engaging investigation into the molecular organization and stage-specific regulation of the Plasmodium chromosomal passenger complex. Its main limitations are in contextual interpretation and methodological clarity rather than data quality. Addressing the points mentioned above, especially regarding nuanced evolutionary context, integrating current literature carefully, clarifying methodological differences, and enhancing figure/data transparency, will significantly improve the rigor, transparency, and fairness of the manuscript's conclusions, thereby increasing its value to the field.

Reviewer #2

(Remarks to the Author)

The study examined aurora-related kinase 1 (Ark1) complexes in Plasmodium berghei gametocytes and ookinetes. The spindle assembly checkpoint controls faithful chromosome segregation. Due to its vital importance, SAC receives substantial attention in studies of apicomplexan parasites. Despite this, SAC remains poorly understood, which is worsened by the Reduction of conventional machinery. The study focuses on the regulation of the kinetochore-MT connection. The authors performed immunoprecipitations from separated male and female parasite lines, providing stage-specific resolution of the mitotic and meiotic ARK1 complexes. This approach led to the detection of alternative CPC complexes across different life stages. It is a significant and original finding in the field of apicomplexan cell biology. However, the study failed to provide a feasible hypothesis of the "modular CPC complexes". The study's novelty is somewhat reduced, as the Ark1-INCEPT interaction is well-documented in eukaryotes and other apicomplexans, as well as the lack of Borealin and Suvivin.

Proposed models of mitosis in gametocytes and the assembly of the Ark1-INCEPT1 and INCEPT2 complexes require better explanation and modeling. The writing is too complex and confusing. The statements are overreaching or based on the wrongly interpreted published data. Many citations are missing.

Major concerns

The SAC "rewriting" in Plasmodium is a strong but weakly supported statement. SAC is a process of kinetochores attachment to spindle fibers. It can be regulated by conventional or unconventional machinery, but it will remain a conserved spindle-assembly checkpoint. The "alternative regulation" makes more sense and does not artificially distance apicomplexans from their relatives, the other eukaryotes. The authors may reconsider the term "rewriting" applied to other processes and complexes across the manuscript.

Lines 89-103: Figure 1 requires a cartoon explaining the mechanics of the three sequential centromere separations. It would be helpful if the authors show their view of the 1N to 8N progression of mitosis. Previous studies confirmed the conventional duplication-elongation-split mechanism, which creates two sets of chromosomes per mitosis. The new model of duplication-elongation-partitioning-split leads to the formation of 4 sets of chromosomes, which contradicts the mitotic rules. It is also unclear how eight kinetochore clusters are formed from one round of mitosis (lines 101-103). Is it possible that the centromere clustering prior to spindle elongation (centromere separation) was missed due to rapid mitosis? A spindle should form between the poles of the duplicated MTOC (between two SMs) that are later partitioned. However, the images show a spindle between duplicated SMs, which I believe is an actual spindle, as centromeres are distributed on it. The statement "Subsequent movement of kinetochores is not driven by retraction of spindle microtubules, but by sequential partitioning of spindle pole MTOCs" does not align well with the evidence. The kinetochores remain spread on the spindle even after the SM portion (rows 3-5). Please present a clear model of the mitosis in the male gametocytes.

The importance of metaphase plate formation for the organism's well-being remains questionable. There is no clear correlation between the lack of centromere alignment at the metaplate in gametocytogenesis (and partial phenotype in ookinetes) and the fidelity of the genetic inheritance. The gametes remain fertile (lines 609-611).

The language is too complex and lacks clarity in delivering the point. The examples: "Evolutionary refinements have subfunctionalized paralogs in most modern-day eukaryotes, reflected not only by the limited phylogenetic signal linking Aurora families, even among animals (Hochegger et al., 2013), but by functional mosaicism in highly derived systems." "Kinetochores align along the spindle midplane during meiosis I, and further MTOC duplication is delayed in the absence of proper chromosome segregation, together consistent with a surveillance mechanism and transmission-fidelity checkpoint that safeguards against the propagation of aneuploidy." "The presence of a minimal machinery that permits transmission efficiency over occasional segregation errors, whilst retaining the capacity for greater fidelity of chromosome segregation when required, aligns with eukaryotic principles observed where the SAC can be modulated or bypassed entirely under certain physiological or developmental constraints."

Overreaching conclusion from the published evidence. The examples:

- "Phylogenetic patterns suggest this event occurred before the diversification of most modern apicomplexans, giving rise to the two INCENPs seen in Hematozoa and Coccidia and coinciding with the loss of SURVIVIN and BOREALIN".
- "Early Drosophila embryos exhibit reduced SAC stringency, facilitating rapid cycling of mitosis without intermittent gap phases." The cited publications did not evaluate the stringency of the SAC.

The model and discussion suggest that Ark1 can bind INCEPT1 and INCEPT2 simultaneously. The Aurora's INCEPT binding site is well-characterized and typically occurs once per Aurora molecule. It is an interesting hypothesis, but the data do not support it. Does Plasmodium Ark1 have two INCEPT binding sites? The fact that Ark1 interacts with two INCEPTs in ookinetes may mean that two different complexes are formed to serve two different functions (meiosis I versus meiosis II?). The differences between INCEPT1 and INCEPT2 complexes can help address this question.

Minor

How is the spindle MTOC (SM) different from the Plasmodium centrosomal plaque (SP)? If not, then the traditional SP abbreviation would be more appropriate.

Lines 96-97: "the spindle contains more microtubules than would be expected based on the number of diploid (28) chromosomes". The authors should either provide quantifications or remove the sentence, given the fact that the conventional spindle is composed of polar (non-kinetochore) microtubules and K-fibers (kinetochores).

Line 104. Please define the "lack of obvious kinetochore organization". Is it due to the lack of a metaphase plate? Please provide the reference.

Line 117: Did you mean the 14 RBC passages? The 14 rounds of intra-erythrocytic cycles will result in 32758 progenies. Please clarify.

Fig 3E. The Y axis measures "spindle pole per MTOC". The spindle pole is the MTOC. Did you mean a bipolar spindle? Then the ratio should be one spindle per two MTOCs (duplicated and separated).

Line 152: "analogous" CPC subunits. Apicomplexans have CPC orthologs. Please correct.

Lines 218-220. Please provide the reference for the increased errors in chromosome segregation of male gametocytes.

Lines 236-239. Please provide the reference

Lines 585-599 lack references for plants, yeasts, trypanosomes, and Plasmodium.

Line 610: Congress? Do you mean condense?

Lines 599-601: Please provide the reference.

Line 641: "It is quite possible, therefore, that INCENP2, rather than INCENP1, is the primary CPC subunit enabling ARK1 function in promoting chromosome biorientation." Could you please clarify how you arrived at this conclusion?

Line 669. "Permissive, error-prone chromosome segregation". It is a confusing and opposite description.

Fig. 8. The model is not informative; Ark1 and INCENPs appear as inactive inhabitants of kinetochores and MTOCs.

Reviewer #3

(Remarks to the Author)
The work presented in this manuscript by Roques etal, is substantial in scope and adds to our understanding of Plasmodium mitosis and meiosis. A major finding of the work is the discovery of a minimal CPC, that functions to allow error prone chromosomal separation during microgamete formation but high-fidelity inheritance during zygotes. The manuscript was generally well written, however due to the scope of the work (number of cell lines life cycle stages examined) it was sometimes hard to follow exactly what has been done on the first reading of the text. There were also some parts where there was a lot of assumed knowledge about previous studies and cell lines. Ot may be useful to add some text in these instance to help with the flow and accessibility to non Plasmodium readers. I have suggested below some areas where text changes may help with the flow of the paper and some suggestions of adding schematics to make some images more accessible, particularly to people outside of Plasmodium. It would also be good to see additional examples of images added to the supplementary. In many cases detailed quantification has been done but extra images would help with convincing the reader of localisation patterns etc and defects. It would also be good to see some additional controls for the initial PAC experiments, especially given this is a central part of the paper and a tool used throughout the work. Below are some specific questions and comments that require addressing.
Comments and questions
Line 90.
In asexual stages and ookinetes NDC80 labelled kinetochores have been previously shown to align. This arrangement is restricted to a very short time period before the kiteochores are spread out along the microtubules. Is it possible that this has been missed in the expansion imaging? Could this occur directly following the initial duplication of the MTOC when they are closer together than in the image displayed in figure1?
Figure 1A.
There is no colocation data shown to validate the AKiT5-mNeonGreen-HA parasite line with another kinetochore marker by expansion microscopy. There is also no reference to the paper where this line was generated. This needs to be supplied. It could be useful to add a line in explaining this cell line in the text and why you are using this rather than something else. Also please list which antibodies were used anti-HA or anti-neonGreen antibody in the legend?
There appears to be a large number of AKiT5 puncta that are not associated with the microtubules in the 1-3min panels particularly. The association seems to be more convincing later in development. The colocation experiments as suggested above as well as some additional examples in a supplementary would also be useful to understand what is going on here.
It is also difficult to see and interpret the mitosis specific processes with the heavily stained axonemes. A schematic representation accompanying each panel I think would help you better highlight the spindle microtubules and kinetochore staining.
There seems like there is a considerable leap in the morphology and cellular arrangements from the 1-3min to 3-6 minute timepoint panels shown.
Why does the DNA staining pattern not change at any point in the development? Would you not expect to see the nucleus starting to associate with the microgametes particularly in the latest time point? Is this the same with other DNA stains?

Fig 1B-E
The artificial chromosome section needs to be redrafted to make the experimental design and outcomes of the experiment clearer. While the experiment design is covered in the supplementary and methods it would be useful to describe the workflow in the main text in a little more detail as it would make the experiment much more understandable on the first read.
I like the suggestion that sex specific transcriptional silencing could be a plausible mechanism for why BFP2 fluorescence is lost. It is important to confirm that the loss of fluorescence is not due to some artifact or another technical reason. Could this be tested by swapping the reporter cassettes, so the PAC is delivered in the female?

In the original Iwagana paper cited, they see faithful inheritance of the PAC through mosquito stages with almost all subsequent sporozoites expressing the reporter. Could your observed phenotype be related to the P28 reporter somehow? Could you use something else? Why do you see p28-mscarlet ookinete/female marker in the male cells? Is there another way to look at native chromosome inheritance?
Fig2 D – the yellow labels are difficult to see. Change the colours.
Line 170 – A western blot should be supplied to confirm correct expression of the INCENP1-mBFP2-2xTy. Also are the images in 2E live cell microscopy? Or is this IFA? Which antibodies were used. This should be added to the legend. This is another part where a little more explanation fo the cell liens and their use would have been good.
There appears to be some mismatches here with the figures and figurelegends as well that need correcting. Figure S2 legend – part F and G are missing. Also line 173 refers to FigS2G being an IFA co location image? Fig 2E legend is also missing?
Line 176 – I think that the data shows that ARK1 and INCENP1 are strong interactors, but it does not say they are the only components. I would change this statement to say Plasmodium appears to have a reduced CPC with 2 core components. Rather than an absolute statement to give you an out in the future.
Line 180- Why did you choose a promoter swap here rather than a conditional KO?
Fig 3D – There appears to be many smaller AKiT5 puncta in the Pska-ARK1 panel than in the control cell. Is this signal specific or background? You should also show additional example images.
This image also shows clustering of all of the MTOCs/kinetochores etc to one side of the parasite. Is this a consistent pattern across all cells. This is reminiscent of some of the other segregation defects seen by others in asexual stage parasites.
Fig 3I – I like this analysis it seems to be good way to distil the data. It might however be better to start this section describing the measurements that were made referencing the images, then the explanation of the PCA.
Line 240 – can you please clarify this statement. In Zeeshan etal there appears to be NDC80 staining in all stages of ookinetes and it also shows kinetochore alignments. In Brusini etal there is co-location of NUF2 with AKiT5. Are you suggesting here that the NDC80-NUF2 and AKiT5 are not associated? Co-location experiments and expansion microscopy would be need to support this claim.
Fig 5A panel iii – there appears to be some puncta that are also decorating along the microtubules. This should be noted.
Line 260 – In this section where you use the ARK1 PAC's it would be useful to have more of an explanation of what the constructs are. This will help with the understanding and interpretation
Figure 6 – Again it would be good to see additional example images of the patterns observed.
Figure 7 B - the labels on the top of the panels on the right. Should be centred with tubulin/marker/ARK1 to make it clear this is refering to the colours in the merges below. You could also indicate that these are merged images.
Fig 7- You see NUF 2 puncta in 7E at 6h and some accumulation already at 4 h. This is different to the NUF 2 pattern shown in 7A where NUF 2 is not observed as puncta until 8h.
Discussion – The phenotype observed in the ARK depleted males where you see a disconnection of the outer MTOC from the spindle and kinetochores is similar to what has been previously observed following deletion of NDC80. This leads to a defect in segregation of nuclei. Other, albeit milder. defects have also been observed following deletion of mini-chromosome maintenance complex binding-protein and schizont egress antigen-1. This should be discussed.

Version 1:

Reviewer comments:

Reviewer #1

(Remarks to the Author)
I want to thank and congratulate all authors for resubmitting a rigorously revised manuscript.
I have no further comments other than congratulations.

Reviewer #2

(Remarks to the Author)
The study offers a new perspective on mitosis in apicomplexan parasites. Using the Plasmodium berghei model, the authors demonstrated a flexible organization of the core mitotic machinery that adapts to the parasite's different life-stage forms. The revised manuscript is substantially improved; all major concerns have been properly addressed. The corrected study is exciting and contributes significantly to the cell biology of apicomplexan parasites and overall biology of eukaryotes.

A few minor suggestions that authors may want to consider:

Lines 64-66: Please correct. The improper kinetochore attachment results in metaphase (not anaphase) arrest at SAC. Indeed, anaphase arrest is not achievable, but it is not related to SAC malfunction. Also, examination of Toxoplasma SAC confirmed the metaphase arrest using conditional downregulation of the SAC CDK-cyclin complex (Hawkins et al., 2021).

Line 886: Recent study in Toxoplasma demonstrated DNA replication machinery engagement to centromeric regions at the onset of S-phase, suggesting that centromeres are the first regions to replicate in Toxoplasma (PMID: 40590555). It indirectly supports the findings of this study.

Reviewer #3

(Remarks to the Author)
The authors have produced a significantly improved manuscript which is easy to read and shows of this important new data. They have adequately addressed all of my comments and concerns from my first review. Below are a few minor comments/correction. Again congratulations on the work.
Minor corrections
Fig 2 b sort 10? Or 10^6 in second line down.
Fig 3d why do you have the live cell panel as part of d? and not on its own as e
Line 283-285 – remove the word "reduced"
Fig7b. It would be better to give each panel its own letter as while related they are 4 different sets of data.

Open Access This Peer Review File is licensed under a Creative Commons Attribution 4.0 International License, which permits use, sharing, adaptation, distribution and reproduction in any medium or format, as long as you give appropriate credit to the original author(s) and the source, provide a link to the Creative Commons license, and indicate if changes were made.
In cases where reviewers are anonymous, credit should be given to 'Anonymous Referee' and the source.
The images or other third party material in this Peer Review File are included in the article's Creative Commons license, unless indicated otherwise in a credit line to the material. If material is not included in the article's Creative Commons license and your intended use is not permitted by statutory regulation or exceeds the permitted use, you will need to obtain permission directly from the copyright holder.
To view a copy of this license, visit https://creativecommons.org/licenses/by/4.0/

RESPONSE TO REVIEWERS

We thank the reviewers for their reading of the manuscript and for their constructive and insightful comments. We have substantially revised the manuscript in response. In particular, we have:

1. *softened and clarified the conceptual framing throughout the manuscript, replacing broad statements with more precise stage- and lineage-specific language;*
2. *integrated recent literature, including Li et al. and Nagar et al., more explicitly throughout the Introduction, Results and Discussion;*
3. *clarified the experimental basis of the ARK1 interactomes, explicitly distinguishing our direct immunoprecipitation–mass spectrometry approach from proximity-labeling methods;*
4. *reorganised and expanded the chromosome inheritance experiments into a dedicated figure and supplementary workflow, with additional reporter controls and revised analysis of blood-stage versus post-fertilisation transmission fidelity;*
5. *Included a phylogenetic study that explicitly concerns INCENP duplication timing and cross-lineage orthology.*
6. *expanded the Methods, revised multiple figure legends, added or clarified schematics, and included additional image examples and validation panels;*
7. *revised the Discussion extensively to better distinguish supported conclusions from more speculative interpretation.*

We respond to each reviewer below.

Sincerely,

Lorenzo Brusini

Reviewer #1:

Summary

This study investigates how Aurora-related kinase 1 (ARK1) coordinates chromosome segregation during Plasmodium berghei sexual development. Using proximity-labeling proteomics (BioID-like), imaging, and functional assays, the authors suggest that *Plasmodium's* chromosomal passenger complex (CPC) acts as a stage-specific modular system, featuring a minimal ARK1–INCENP-A module in male gametogony and an expanded CPC that includes kinetochore components during meiosis. The work offers valuable mechanistic insights into how this parasite adapts its mitotic and meiotic machinery to its unique life cycle.

The dataset is impressive and technically advanced, and the study is likely to become a valuable resource for the community. However, several claims are overstated, and some key recent literature, particularly the studies by Li et al. (2024, PMID: 38987258) and Nagar et al. (2025, PMID: 40909691), should be recognized to contextualize this work appropriately.

We thank the reviewer for the thoughtful and constructive assessment of our study and for highlighting the importance of more cautious framing and broader contextualisation. We have revised the manuscript accordingly.

Major Comments

1. Conceptual framing and overstatements

a) Scope of "rewiring" claims:

The manuscript often states that *Plasmodium* "rewires eukaryotic checkpoint control." These conclusions mainly come from *P. berghei* sexual stages and do not include recent structural and mechanistic data from asexual stages (Li et al. (2024, PMID: 38987258)) showing conservation of spindle–kinetochore–centrosome coupling. The language should be more cautious and based on proven differences rather than broad evolutionary generalizations. The manuscript's claim of "rewiring of checkpoint control" in *Plasmodium* should be more cautiously framed as illustrating the plasticity and adaptation of cell division machinery within a limited phylogenetic and life stage context. Comprehensive comparative data, such as those provided by Nagar et al. (2025, PMID: 40909691), demonstrate divergent CPC architectures and checkpoint components across Apicomplexa, including losses and replacements in lineages like Cryptosporidium.

Thus, descriptions of CPC "modularity" and stage-specific composition should be contextualized as possible lineage-specific innovations rather than broadly conserved eukaryotic features. Explicit acknowledgment of these contemporary works and phylogenetic nuance will improve accuracy and prevent overgeneralization.

We agree our earlier wording was too broad in places. We have therefore revised the Introduction (lines 66-70) and Discussion (lines 989-1020) to avoid implying general rewiring of eukaryotic

checkpoint control. Instead, we now frame our conclusions more precisely as demonstrating developmental plasticity in chromosome-segregation regulation during sexual development of Plasmodium berghei.

In the revised manuscript, we explicitly distinguish:

1. *conserved kinetochore–spindle–MTOC coupling (lines 56-61), which is consistent with both our previous work (Brusini et al., 2022) and recent data from asexual blood stages (Li et al., 2024); and*
2. *modularity of CPC composition and attachment-regulatory mechanisms across distinct sexual-stage division programmes (lines 86-90).*

Thus, our central claim is now that P. berghei deploys distinct ARK1-centred CPC states in male gametogenesis versus zygote/ookinete meiosis.

b) Novelty of CPC modularity:

The characterization of the CPC's modularity as "previously unrecognized" is overstated. Nagar et al. (2025, PMID: 40909691) independently report ARK1's role in spindle/kinetochore regulation, the identification of two INCENPs, and the absence of Survivin/Borealin, demonstrating CPC divergence and stage-specific inclusion. The present study provides complementary spatial and functional insights, but it is not the first to recognize CPC modularity. It would strengthen the Discussion to acknowledge this concurrent work explicitly.

We thank the reviewer for highlighting the complementary work by Nagar et al. We now explicitly acknowledge this study in the revised Discussion (959-961, 980-983). We note, however, that broader divergence of the apicomplexan CPC, including the presence of two INCENP paralogs and the lack of obvious SURVIVIN/BOREALIN orthologs, had already been established in work we already explicitly cited (Komaki et al., 2022) and we do not claim priority for these observations. Accordingly, we have removed wording that could imply that divergence of the CPC was "previously unrecognized," and we now distinguish more clearly between

- ***CPC divergence**, which is supported by comparative/evolutionary work, including our own, and*
- ***developmental modularity**, which in our manuscript refers specifically to deployment of distinct ARK1-centred CPC compositions during male mitosis versus meiosis.*

Importantly, Nagar et al. do not describe the CPC as modular, nor do they define or experimentally test CPC modularity. The term "modular" does not appear in their manuscript (Nagar et al., 2026), and their analyses do not address stage-specific deployment or function of CPC components past ARK1. We therefore now present the novelty of the present study more precisely as the biochemical, functional and spatial dissection of CPC composition across sexual-

stage division modes, compared to previous recognition that the Plasmodium CPC is divergent compared to that in animals and fungi.

c) Generality across Apicomplexa:

The mechanistic data and evolutionary inferences in this study are solely based on experiments with *Plasmodium berghei*. Yet, the Discussion often generalizes these findings to “apicomplexan” and even broader “eukaryotic” principles. These broad generalizations should be tempered, as cell-division mechanisms, checkpoint control, and molecular structures vary significantly among different apicomplexan species, including *Plasmodium falciparum, Toxoplasma gondii, Babesia spp.*, and especially *Cryptosporidium*, as well as among stages within a single species.

Recent comparative research by Nagar et al. (2025, PMID: 40909691) systematically examined ARK kinases and chromosomal passenger complex (CPC) components across the Apicomplexa. It revealed that, while *Plasmodium* and *Toxoplasma* share a diverged CPC lacking Survivin and Borealin, lineages like Cryptosporidium have lost or replaced many of the core CPC proteins entirely. These findings emphasize that claims about CPC modularity or structure should be considered lineage-specific hypotheses rather than universal features of the phylum. Including or referencing such cross-lineage comparative work would improve phylogenetic accuracy and strengthen the evolutionary Discussion, ensuring that conclusions based on *P. berghei* are contextualized adequately within the remarkable diversity and adaptability of apicomplexan cell-division strategies.

We agree that our experimental evidence presented in this study, which builds upon our previous functional analyses in P. berghei and T. gondii (Brusini et al., 2022), is restricted to P. berghei and that generalization beyond this context should be cautious. We have therefore revised the manuscript to avoid broad “pan-apicomplexan” mechanistic claims unless explicitly supported by comparative data.

In particular:

- *A phylogenetic study has been included (Fig. 8A-C, Fig. S7), especially with respect to our latest results that explicitly concerns INCENP duplication timing and cross-lineage orthology (lines 385-420).*
- *references to “Apicomplexa” in the Discussion now primarily concern correlation between our previous functional analyses in P. berghei and T. gondii with our evolutionary analysis shown here (lines 999-1020);*
- *mechanistic conclusions are explicitly stated as applying to P. berghei sexual stages unless otherwise indicated; and*

We now present broader apicomplexan relevance as a hypothesis-generating evolutionary framework, rather than as a demonstrated universal feature.

2. Integration with existing literature

Li et al. (2024, PMID: 38987258):

This expansion microscopy study showed that losing kinetochore proteins PfNDC80 or PfNuf2 causes spindle–centriole-like plaque detachment and results in the formation of anucleate merozoites, revealing conserved mechanisms of kinetochore–MTOC anchoring during the asexual stages. This significant work should be cited when discussing the structural connection between the kinetochore and centrosomal counterparts.

We have strengthened integration of Li et al. in the revised manuscript past its previous citation. We now cite Li et al. in both the Introduction (lines 58-61), Results (lines 267-271) and Discussion (lines 925-933), in particular with respect to ARK1 depletion phenotypes in male gametogenesis, MTOC/spindle decoupling and the increase in free outer MTOCs/basal bodies. This clearly links our sexual-stage observations to the previously described NDC80/NUF2-dependent kinetochore–MTOC anchoring defects in asexual blood stages.

Nagar et al. (now 2026, PMID 41748607)

This contemporaneous study provides a complementary ARK1 interactome obtained through GFP-based immunoprecipitation. It covers a broader range of life stages, including asexual schizogony, and offers detailed comparative genomics that reveal diversity in CPC composition across Apicomplexa. Including a brief acknowledgment of this work will help readers understand both studies within the context of ongoing *Plasmodium* cell division research.

We agree that Nagar et al. provides complementary context. We now discuss this work explicitly in the Discussion in two ways. First, we acknowledge that Nagar et al. supports the existence of an ARK1-centred CPC containing two INCENP paralogs in Plasmodium (lines 958-967). Second, we distinguish our contribution from theirs more clearly (lines 966-974). Nagar et al. uniquely includes transcriptomic data and blood-stage interactome and functional data. Our study uniquely resolves sex-separated, stage-specific CPC assemblies, localisations and functions during male mitosis versus zygote meiosis, throughout the entire lifecycle through bite-back experiments to re-introduce mutant parasite lines in mice. These distinctions are now made explicitly in the revised text.

3. Methodological distinctions and limitations

3.1. Proximity labeling vs. immunoprecipitation:

The ARK1 interactome data in this manuscript are derived from proximity labeling combined with cross-linking, which identifies proteins in the spatial neighborhood of ARK1 rather than solely direct binders. This distinction should be explicitly emphasized, especially when interpreting the composition and structure of the chromosomal passenger complex. In comparison, Nagar et al. (2025, PMID 40909691) used direct co-immunoprecipitation coupled with mass spectrometry (IP-MS), which selectively enriches for stable and direct interactions. The authors might note that

proximity labeling offers complementary insights by capturing transient or spatially restricted interactions, but it requires cautious interpretation to prevent overcalling stable complexes.

This interpretation is not accurate. Our ARK1 interactomes were generated by direct immunoprecipitation of endogenous HA-tagged ARK1 using anti-HA–coupled beads. We have further made this explicit throughout the Methods (lines 1335-1352) and Results (lines 208-218).

We further clarify that:

- *no BioID/TurboID/APEX-type enzyme was used;*
- *both native and formaldehyde cross-linking conditions were used, with cross-linking crucial to stabilise more labile protein associations; and*
- *comparison of native vs. cross-linked immunoprecipitations was used specifically to help distinguish stable core interactions from more labile associations.*

Caveats on stage extrapolation:

The study's conclusions primarily rely on sexual-stage data, such as male gametogony and zygote/ookinete development. The manuscript should temper claims that these modular CPC mechanisms universally apply to all *Plasmodium* mitoses, including asexual blood stages, which feature distinct cell cycle characteristics and regulatory structures. Additional experimental validation in other stages is necessary before making broad generalizations.

We agree that the conclusions of this manuscript are based primarily on sexual-stage divisions. We have revised the Discussion to make clear any extrapolation to blood-stage schizogony is now presented explicitly as a hypothesis (lines 958-966), albeit one now also supported by the broader context provided by Nagar et al. by the presence of both INCENP paralogs in asexual blood-stage interactomes, consistent with the canonical metaphase plate seen during schizogony and meiosis (Fig. S1, Fig. 6, Fig. S5; Brusini et al., 2022),

4. Data presentation and figure clarity

Figure formatting:

In Figure 3, axis labels overlap and truncate protein names. Adjust figure layout for legibility. For Figure 1, add quantification of biological replicates (currently unclear).

We have revised the figure layouts and legends accordingly. In particular:

- *Figure 1 now includes quantification and supporting schematics.*
- *Protein labels and axes in the interactome figures have been reformatted for readability.*

5. Comparative and conceptual suggestions

Clarify the differences between ARK1's proximity labeling interactome data (this study) and the direct immunoprecipitation-based interactome reported by Nagar et al. (2025, PMID 40909691), explicitly discussing which interactions may represent stable versus transient or spatially proximal

associations. This distinction is important for understanding the composition and dynamics of the chromosomal passenger complex.

Highlight that Nagar et al. (2025, PMID 40909691) also provide complementary transcriptomic (RNA-Seq) and imaging data that support broader functional roles of ARK1 across different life stages. These datasets should be acknowledged in the Discussion as supporting and expanding the mechanistic insights presented here.

Recommend reframing any broad claims of "pan-apicomplexan" conservation or rewiring with specificity regarding the taxonomic lineages and life cycle stages where these findings have been validated. This will help readers clearly understand the evolutionary scope and limitations.

Suggest including a graphical summary or model figure contrasting canonical, divergent, and modular architectures of the chromosomal passenger complex, integrating ARK1, INCENP paralogs, and kinetochore markers (e.g., AKiT proteins), to better visualize and contextualize the current findings within the broader field.

We have incorporated these suggestions as follows:

- *We now explicitly state our ARK1 interactomes were generated by direct immunoprecipitation of endogenous HA-tagged ARK1 using anti-HA–coupled beads (lines 1336-1338).*
- *We now acknowledge Nagar et al.'s transcriptomic datasets as complementary context, while clarifying that such datasets do not resolve CPC composition, interaction stability or stage-specific assembly (lines 980-988).*
- *Broad claims of pan-apicomplexan conservation have been narrowed substantially.*
- *A model of Plasmodium kinetochores has been added (Fig. S1B) and revised graphical summary/model has been included to better visualise the distinction between the reduced male CPC state and the expanded meiotic CPC state (Fig. 8D).*

Overall assessment

This study offers a technically sound and visually engaging investigation into the molecular organization and stage-specific regulation of the *Plasmodium* chromosomal passenger complex. Its main limitations are in contextual interpretation and methodological clarity rather than data quality. Addressing the points mentioned above, especially regarding nuanced evolutionary context, integrating current literature carefully, clarifying methodological differences, and enhancing figure/data transparency, will significantly improve the rigor, transparency, and fairness of the manuscript's conclusions, thereby increasing its value to the field.

We thank the reviewer for these valuable suggestions, which have helped substantially improve the precision and balance of the manuscript.

Reviewer #2:

The study examined aurora-related kinase 1 (Ark1) complexes in *Plasmodium berghei* gametocytes and ookinetes. The spindle assembly checkpoint controls faithful chromosome segregation. Due to its vital importance, SAC receives substantial attention in studies of apicomplexan parasites. Despite this, SAC remains poorly understood, which is worsened by the Reduction of conventional machinery. The study focuses on the regulation of the kinetochore-MT connection. The authors performed immunoprecipitations from separated male and female parasite lines, providing stage-specific resolution of the mitotic and meiotic ARK1 complexes. This approach led to the detection of alternative CPC complexes across different life stages. It is a significant and original finding in the field of apicomplexan cell biology. However, the study failed to provide a feasible hypothesis of the "modular CPC complexes". The study's novelty is somewhat reduced, as the Ark1-INCEPT interaction is well-documented in eukaryotes and other apicomplexans, as well as the lack of Borealin and Suvivin. Proposed models of mitosis in gametocytes and the assembly of the Ark1-INCEPT1 and INCEPT2 complexes require better explanation and modeling. The writing is too complex and confusing. The statements are overreaching or based on the wrongly interpreted published data. Many citations are missing.

Major concerns

1. The SAC "rewriting" in *Plasmodium* is a strong but weakly supported statement. SAC is a process of kinetochores attachment to spindle fibers. It can be regulated by conventional or unconventional machinery, but it will remain a conserved spindle-assembly checkpoint. The "alternative regulation" makes more sense and does not artificially distance apicomplexans from their relatives, the other eukaryotes. The authors may reconsider the term "rewriting" applied to other processes and complexes across the manuscript.

We thank the reviewer and agree that the core logic of kinetochore–microtubule attachment surveillance is a fundamental feature of eukaryotic cell division. However, we respectfully note that functional analogy should not be conflated with molecular conservation. Plasmodium species have recognizable kinetochores with inner CCAN and outer KMN modules. Yet the kinetochore, and the canonical SAC/CPC toolkit, is markedly reduced relative to animals and fungi, with multiple recruitment factors absent and stage-specific deployment of the remaining components. In this sense, our use of "rewiring" does not imply loss of checkpoint logic, but to the ***reconfiguration of machinery inherited by vertical descent*** *underlying attachment regulation in P. berghei. We now make this definition explicit in the Discussion (lines 906-907). Specifically, our data support distinct ARK1-centred CPC states in male gametogenesis versus zygote/ookinete meiosis. To avoid overstating the scope of this conclusion, we have revised the manuscript text to confine this claim to chromosome-segregation control during P. berghei sexual development.*

2. Lines 89-103: Figure 1 requires a cartoon explaining the mechanics of the three sequential centromere separations. It would be helpful if the authors show their view of the 1N to 8N progression of mitosis. Previous studies confirmed the conventional duplication-elongation-split mechanism, which creates two sets of chromosomes per mitosis. The new model of duplication-elongation-partitioning-split leads to the formation of 4 sets of chromosomes, which contradicts the mitotic rules. It is also unclear how eight kinetochore clusters are formed from one round of mitosis (lines 101-103). Is it possible that the centromere clustering prior to spindle elongation (centromere separation) was missed due to rapid mitosis? A spindle should form between the poles of the duplicated MTOC (between two SMs) that are later partitioned. However, the images show a spindle between duplicated SMs, which I believe is an actual spindle, as centromeres are distributed on it. The statement "Subsequent movement of kinetochores is not driven by retraction of spindle microtubules, but by sequential partitioning of spindle pole MTOCs" does not align well with the evidence. The kinetochores remain spread on the spindle even after the SM portion (rows 3-5). Please present a clear model of the mitosis in the male gametocytes.

We appreciate this detailed point and have substantially revised this section of the manuscript. To improve clarity, we have:

- *added schematics throughout the revised figure set, especially in Figure 1 and Figure S1;*
- *expanded the male U-ExM sampling to include non-activated and early post-activation states;*
- *clarified how the bipartite MTOC architecture was used to distinguish intranuclear spindle structures from axonemal structures (lines 113-119)*
- *Revised the Discussion (lines 866-883) to present our model explicitly as an interpretation supported by quantitative 3D volumetric analysis, rather than as a definitive reconstruction of every transient intermediate.*

Most importantly, our revised analysis is now based on >160 fully reconstructed 3D cells spanning non-activated and tightly spaced post-activation time points. Across this dataset, we do not observe a convincing example of sustained metaphase-like kinetochore alignment during male gametogenesis. Instead, the first spindle contains substantially more spindle microtubule and kinetochore material than later spindles, consistent with progressive partitioning of an initially larger spindle–kinetochore complement rather than a simple sequential diploid duplication–elongation–split cycle (lines 141-145, 872–885). We recognize that this interpretation is unusual, and we cannot formally exclude a very short-lived alignment event below our temporal resolution. However, the revised data are most consistent with progressive MTOC partitioning and supports early descriptions derived from electron microscopy that also concluded a reduction from a "larger" initial complement to a "smaller" terminal one (Sinden et al. 1976). We therefore now present this model more cautiously as the best-supported interpretation of the current data, and

we place the centromere-first replication idea explicitly as a speculative hypothesis rather than a conclusion (lines 885-889).

3. The importance of metaphase plate formation for the organism's well-being remains questionable. There is no clear correlation between the lack of centromere alignment at the metaplate in gametocytogenesis (and partial phenotype in ookinetes) and the fidelity of the genetic inheritance. The gametes remain fertile (lines 609-611).

We respectfully disagree with this interpretation. Across eukaryotes, accurate chromosome congression and kinetochore biorientation are widely associated with faithful chromosome segregation. Perturbations that impair the establishment of biorientation increase the frequency of segregation errors, including lagging chromosomes, micronuclei or aneuploidy (Yucel et al., 2000; Antonio et al., 2000; Cimini et al., 2001; Niu et al., 2015; Edwards et al., 2018; Fonseca et al., 2019; Vukušić and Tolić, 2022; Ballmer et al., 2024). We have now made this point explicit (lines 147-151).

At the same time, we agree that our previous wording may have implied a stronger causal claim than our data alone can prove and we do not demonstrate that metaphase-like alignment itself is the sole determinant of segregation fidelity in P. berghei. We have therefore revised the manuscript and now explicitly note that kinetochore congression/alignment are cues generally associated with correction of erroneous kinetochore–spindle interactions, and we directly compare chromosome retention during blood-stage proliferation with paternal transmission after fertilisation (lines 884-898).

To be clear, asexual blood-stage mitosis and meiosis both show obvious kinetochore congression and alignment, whereas male microgametogenesis does not. This correlates with uniquely permissive chromosome transmission, as shown in our chromosome-inheritance assays in Figure 2. Notably, even the endogenous paternal SIL6 reporter is not fully retained in mature ookinetes.

Regarding the suggestion of a "partial phenotype in ookinetes," we may have been unclear previously. Meiosis in Plasmodium is characterised by ordered kinetochore alignment and synaptonemal-complex formation, as described by electron microscopy (Sinden et al., 1985), and meiotic kinetochore alignment was also demonstrated in our previous U-ExM study (Brusini et al., 2022). In our revised meiotic analysis, the dominant configuration is an ordered bipolar spindle with aligned kinetochore states consistent with end-on kinetochore–microtubule attachment and biorientation (lines 306–320), unlike male microgametogenesis. We do note that a subset of meiotic cells shows 3 MTOCs and multipolar spindles, so we do not claim complete uniformity; rather, our point is that meiosis does not recapitulate the male segregation programme.

Finally, we distinguish fertility from faithful genetic inheritance. The ability of male gametes to fertilise and initiate ookinete development is not equivalent to full paternal genome transmission. Indeed, ookinete differentiation proceeds despite incomplete nuclear division or abnormal

genome inheritance (Bushell et al., 2009; Ning et al., 2013). This distinction is central to the interpretation of our data and our inheritance assays show that sexual development can proceed despite incomplete paternal chromosome transmission.

4. The language is too complex and lacks clarity in delivering the point. The examples: "Evolutionary refinements have subfunctionalized paralogs in most modern-day eukaryotes, reflected not only by the limited phylogenetic signal linking Aurora families, even among animals (Hochegger et al., 2013), but by functional mosaicism in highly derived systems." "Kinetochores align along the spindle midplane during meiosis I, and further MTOC duplication is delayed in the absence of proper chromosome segregation, together consistent with a surveillance mechanism and transmission-fidelity checkpoint that safeguards against the propagation of aneuploidy." "The presence of a minimal machinery that permits transmission efficiency over occasional segregation errors, whilst retaining the capacity for greater fidelity of chromosome segregation when required, aligns with eukaryotic principles observed where the SAC can be modulated or bypassed entirely under certain physiological or developmental constraints."

The manuscript has been simplified. In particular, we have revised long or over-compressed sentences, and presented the major biological conclusions more directly.

5. Overreaching conclusion from the published evidence. The examples: "Phylogenetic patterns suggest this event occurred before the diversification of most modern apicomplexans, giving rise to the two INCENPs seen in Hematozoa and Coccidia and coinciding with the loss of SURVIVIN and BOREALIN".

"Early Drosophila embryos exhibit reduced SAC stringency, facilitating rapid cycling of mitosis without intermittent gap phases." The cited publications did not evaluate the stringency of the SAC.

The model and discussion suggest that Ark1 can bind INCEPT1 and INCEPT2 simultaneously. The Aurora's INCEPT binding site is well-characterized and typically occurs once per Aurora molecule. It is an interesting hypothesis, but the data do not support it. Does Plasmodium Ark1 have two INCEPT binding sites? The fact that Ark1 interacts with two INCEPTs in ookinetes may mean that two different complexes are formed to serve two different functions (meiosis I versus meiosis II?). The differences between INCEPT1 and INCEPT2 complexes can help address this question.

INCENP evolution

An evolutionary comparative analysis section on INCENP in Apicomplexa has been included. We no longer claim that the timing of INCENP duplication has been resolved as our latest data is unable to distinguish between either an ancestral or parallel duplication, leaving the situation unresolved (Figure 8A-C, Figure S7). Instead, we now state that:

- *full-length phylogenies recover sensible lineage-level clades but do not strongly resolve deeper relationships;*
- *IN-box-restricted analyses support cross-lineage A-like and B-like similarity classes;*
- *AU tests do not decisively reject either parallel-duplication or ancient-core duplication models; and*
- *strong one-to-one orthology assignments are therefore not clear or justified.*

Drosophila/SAC stringency

The Discussion has been extensively re-written and this comparison is no longer present.

Simultaneous binding of two INCENPs

We agree that our earlier model was misleading and implied simultaneous binding of INCENP-A and INCENP-B to a single ARK1 molecule, for which we have no evidence. We have revised the model in Figure 8D to avoid this implication as absolute CPC conformations have not been demonstrated in this study.

The revised manuscript now states more conservatively that:

- *ARK1 forms a stable core complex with INCENP-A during male gametogenesis; and*
- *In the meiotic context, ARK1 is recovered in association with both INCENP-A and INCENP-B, consistent with an expanded meiotic CPC state.*

Minor concerns

How is the spindle MTOC (SM) different from the *Plasmodium* centrosomal plaque (SP)? If not, then the traditional SP abbreviation would be more appropriate.

The term centrosomal plaque (CP) has been used in the context of Plasmodium asexual blood-stage mitosis. The microgametocyte MTOC is structurally and functionally distinct from the asexual CP. In microgametocytes, the MTOC comprises two physically and functionally separable nucleation centres: an inner spindle pole origin of spindle microtubules (denoted IM); an outer basal body that nucleates axoneme microtubules (denoted BB). We have made this explicit in the Results (lines 114-118) and Figure 1B.

Lines 96-97: "the spindle contains more microtubules than would be expected based on the number of diploid (28) chromosomes". The authors should either provide quantifications or remove the sentence, given the fact that the conventional spindle is composed of polar (non-kinetochore) microtubules and K-fibers (kinetochores).

We have included the data to support this claim in Figure 1D, supported by volumetric measurements rather than inferred microtubule counts.

Line 104. Please define the "lack of obvious kinetochore organization". Is it due to the lack of a metaphase plate? Please provide the reference.

Clarified to mean absence of convincing metaphase-like alignment. This is one of our main findings of Figure 1, and previously reported (Brusini et al., 2022).

Line 117: Did you mean the 14 RBC passages? The 14 rounds of intra-erythrocytic cycles will result in 32758 progenies. Please clarify.

In the previous version, we referred to serial RBC passages in order to avoid over-interpreting the exact number of nuclear divisions occurring during each intra-erythrocytic cycle, which in P. berghei typically range from schizonts containing ~16-32 merozoites. However, we agree that expressing the result in that way made comparison with previous PAC studies unclear. To resolve this, we now follow the convention used by Iwanaga et al., in which blood-stage segregation efficiency is expressed per nuclear division, assuming that parasites undergo four nuclear divisions during schizogony over a 24 h erythrocytic cycle (Iwanaga et al., 2010).

This revised workflow is now shown in Fig. 2C–D and our calculations used to estimate chromosome retention during both blood-stage proliferation and sexual-stage transmission are revised and explicitly detailed in the Methods (lines 1275-1336).

Fig 3E. The Y axis measures "spindle pole per MTOC". The spindle pole is the MTOC. Did you mean a bipolar spindle? Then the ratio should be one spindle per two MTOCs (duplicated and separated).

Schematics in Figure 1 and 3 should clarify that the measurement concerns spindle poles relative to MTOC units, not a ratio implying that spindle poles and MTOCs are equivalent objects. Spindle poles relative to MTOC units increases in the ARK1 mutant, creating multipolar spindles. This is a main finding of Figure 3.

Line 152: "analogous" CPC subunits. Apicomplexans have CPC orthologs. Please correct.

Corrected.

Lines 218-220. Please provide the reference for the increased errors in chromosome segregation of male gametocytes.

A main finding of Figure 2.

Lines 236-239. Please provide the reference

Corrected.

Lines 585-599 lack references for plants, yeasts, trypanosomes, and Plasmodium.

These missing references have been added (lines 990-1003)

Line 610: Congress? Do you mean condense?

In animals and fungi, kinetochores "congress" at prometaphase towards the equatorial plate for metaphase alignment. We have previously shown this also in P. berghei and T. gondii (Brusini et al., 2022).

Lines 599-601: Please provide the reference.

Corrected.

Line 641: "It is quite possible, therefore, that INCENP-B, rather than INCENP-A, is the primary CPC subunit enabling ARK1 function in promoting chromosome biorientation." Could you please clarify how you arrived at this conclusion?

Please see point 3 above. Briefly, this hypothesis is based on the correlation between mitotic architecture and CPC composition:

- *Male gametogenesis = no biorientation = ARK1 + INCENP-A*
- *Meiosis = biorientation = ARK1 + INCENP-A + INCENP-B*

This is now also supported by (Nagar et al., 2026)

- *Asexual mitosis = biorientation = ARK1 + INCENP-A + INCENP-B*

Line 669. "Permissive, error-prone chromosome segregation". It is a confusing and opposite description.

We do not regard these descriptors as contradictory. Evolution allows for imperfection, i.e. sometimes things have to be just "good enough". "Permissive" here refers to a chromosome-segregation regime with reduced stringency of surveillance (lack of metaphase architecture and components), whereas "error-prone" refers to the consequence. Our inheritance data support this interpretation: male microgametogenesis is associated with reduced paternal chromosome transmission. To avoid semantic distraction however, we have revised the manuscript text and this is no longer relevant.

Fig. 8. The model is not informative; Ark1 and INCENPs appear as inactive inhabitants of kinetochores and MTOCs.

We have completely redesigned our model in Figure 8D to give direction for CPC regulation and make the functional distinction between male and meiotic CPC states clearer.

We thank the reviewer again for helping us improve the clarity, caution and interpretability of the manuscript.

Reviewer #3:

The work presented in this manuscript by Roques etal, is substantial in scope and adds to our understanding of Plasmodium mitosis and meiosis. A major finding of the work is the discovery of a minimal CPC, that functions to allow error prone chromosomal separation during microgamete

formation but high-fidelity inheritance during zygotes. The manuscript was generally well written, however due to the scope of the work (number of cell lines life cycle stages examined) it was sometimes hard to follow exactly what has been done on the first reading of the text. There were also some parts where there was a lot of assumed knowledge about previous studies and cell lines. Ot may be useful to add some text in these instance to help with the flow and accessibility to non Plasmodium readers. I have suggested below some areas where text changes may help with the flow of the paper and some suggestions of adding schematics to make some images more accessible, particularly to people outside of Plasmodium. It would also be good to see additional examples of images added to the supplementary. In many cases detailed quantification has been done but extra images would help with convincing the reader of localisation patterns etc and defects. It would also be good to see some additional controls for the initial PAC experiments, especially given this is a central part of the paper and a tool used throughout the work. Below are some specific questions and comments that require addressing.

We thank the reviewer for the careful and constructive reading, and especially for the suggestions aimed at improving accessibility, figure interpretation and experimental clarity. We agree that the manuscript benefits from clearer workflow explanation, more schematics, more supporting image examples, and stronger presentation of the chromosome inheritance assays.

In response to these comments, we have:

- *improved the flow in both the Results and figure legends;*
- *added or revised schematics across figures;*
- *expanded the chromosome inheritance section into a dedicated figure with a clearer workflow;*
- *added additional controls and supporting image examples; and*
- *clarified the rationale and validation for several reporter and depletion lines.*

Comments and questions

1. Line 90. In asexual stages and ookinetes NDC80 labelled kinetochores have been previously shown to align. This arrangement is restricted to a very short time period before the kiteochores are spread out along the microtubules. Is it possible that this has been missed in the expansion imaging? Could this occur directly following the initial duplication of the MTOC when they are closer together than in the image displayed in figure1?

We thank the reviewer for this important point. To address this explicitly, we expanded the sampling of male U-ExM analyses now shown in Figure 1 to include non-activated cells and early post-activation states, including the period immediately following initial MTOC duplication. We also added additional examples in Figure S1 and Supplementary Data 1.

Our revised analysis is now based on >160 fully reconstructed 3D cells spanning non-activated and tightly spaced post-activation time points. Across this dataset, and those previously generated (Brusini et al., 2022), we do not observe a convincing example of sustained metaphase-like kinetochore alignment during male gametogenesis (lines 112–127). We note that we cannot formally exclude a very short-lived alignment event below our temporal resolution, however we conclude that, at least, male kinetochore movements look very different to those seen during blood-stage division and meiosis.

2. Figure 1A. There is no colocation data shown to validate the AKiT5-mNeonGreen-HA parasite line with another kinetochore marker by expansion microscopy. There is also no reference to the paper where this line was generated. This needs to be supplied. It could be useful to add a line in explaining this cell line in the text and why you are using this rather than something else. Also please list which antibodies were used anti-HA or anti-neonGreen antibody in the legend?

The AKiT5-mNeonGreen-3xHA parasite line is referenced at first mention in the Results (previously line 140), However we agree this required clearer explanation. We have therefore:

- *cited the original study validating AKiT5 at first mention (lines 109-112);*
- *added a brief explanation of why AKiT5 was used in this study;*
- *clarified in the figure legends how the tagged protein was detected (line 425); and*
- *added schematics and supporting validation panels to Figure S1A, B, C.*

3. There appears to be a large number of AKiT5 puncta that are not associated with the microtubules in the 1-3min panels particularly. The association seems to be more convincing later in development. The colocation experiments as suggested above as well as some additional examples in a supplementary would also be useful to understand what is going on here. It is also difficult to see and interpret the mitosis specific processes with the heavily stained axonemes. A schematic representation accompanying each panel I think would help you better highlight the spindle microtubules and kinetochore staining. There seems like there is a considerable leap in the morphology and cellular arrangements from the 1-3min to 3-6 minute timepoint panels shown.

We agree that the early time points are difficult to interpret in the original presentation. To address this, we have:

- *added earlier time points to Figure 1;*
- *included schematics alongside the relevant panels Figure 1, and the remaining manuscript, detailing how the MTOC is used to distinguish between axonemal and spindle microtubules;*
- *added additional image examples in Figure S1 and Supplementary Data 1.*

4. Why does the DNA staining pattern not change at any point in the development? Would you not expect to see the nucleus starting to associate with the microgametes particularly in the latest time point? Is this the same with other DNA stains?

We agree that the DNA staining could be misleading in the original presentation. In our hands, DNA staining after U-ExM is variable and strongly influenced by fixation, denaturation and gel-processing steps. Accordingly, we have removed DNA-stain panels from main figures where they are not informative and only misleading, and now rely primarily on protein-based markers and volumetric analyses to infer mitotic progression and chromosome segregation.

5. Fig 1B-E. The artificial chromosome section needs to be redrafted to make the experimental design and outcomes of the experiment clearer. While the experiment design is covered in the supplementary and methods it would be useful to describe the workflow in the main text in a little more detail as it would make the experiment much more understandable on the first read. I like the suggestion that sex specific transcriptional silencing could be a plausible mechanism for why BFP2 fluorescence is lost. It is important to confirm that the loss of fluorescence is not due to some artifact or another technical reason. Could this be tested by swapping the reporter cassettes, so the PAC is delivered in the female? In the original Iwagana paper cited, they see faithful inheritance of the PAC through mosquito stages with almost all subsequent sporozoites expressing the reporter. Could your observed phenotype be related to the P28 reporter somehow? Could you use something else? Why do you see p28-mscarlet ookinete/female marker in the male cells? Is there another way to look at native chromosome inheritance?

We strongly agree and have substantially revised this section.

Specifically, we have:

- *reorganised the chromosome inheritance experiments into a dedicated Figure 2;*
- *expanded the Results (lines 147-189) and Methods (lines 1275-1336) explanations of the workflow;*
- *added an endogenous chromosomal reporter (SIL6) as a reference;*
- *added PAC-B and PAC-C to test cassette orientation/3'UTR effects and local sequence context;*
- *introduced a new imaging-flow-cytometry workflow for mature ookinete scoring (lines 1254-1274) and;*
- *clarified explicitly why previous PAC-based assays could not isolate male segregation fidelity (lines 973-975).*

This last point is particularly important. By introducing the reporter exclusively into the male-only fd^{1-} *line and crossing to untransformed female-only* md^{4-} *parasites, we were able to specifically*

measure male-lineage transmission fidelity, which earlier designs could not isolate as the PAC was already present in the female.

Fig2 D - the yellow labels are difficult to see. Change the colours.

Corrected, now Figure 3C, D.

Line 170 – A western blot should be supplied to confirm correct expression of the INCENP-A-mBFP2-2xTy. Also are the images in 2E live cell microscopy? Or is this IFA? Which antibodies were used. This should be added to the legend. This is another part where a little more explanation fo the cell liens and their use would have been good.

There appears to be some mismatches here with the figures and figurelegends as well that need correcting. Figure S2 legend – part F and G are missing. Also line 173 refers to FigS2G being an IFA co location image? Fig 2E legend is also missing?

Now in Figure 3 and Figure S3A, INCENP line validation / western / legend mismatches: diagnostic PCR and immunoblot validation panels have now been added/clarified, and figure/legend inconsistencies were corrected. Please note INCENP-A protein is large (>280 kD) and we do not readily detect by immunoblot, therefore we also show genomic PCR validation.

Line 176 – I think that the data shows that ARK1 and INCENP-A are strong interactors, but it does not say they are the only components. I would change this statement to say Plasmodium appears to have a reduced CPC with 2 core components. Rather than an absolute statement to give you an out in the future.

We agree that our data do not formally exclude the existence of additional, weakly associated or transient CPC-related factors. However, we think the present dataset supports a stronger conclusion than simply stating that ARK1 and INCENP-A are strong interactors. In Figure 3C, D, under our experimental conditions, ARK1 and INCENP-A are the only pair that behaves as a stable core complex in microgametocytes. INCENP-A is the only CPC component robustly recovered with ARK1 under native conditions, and this interaction remains prominent after cross-linking. By contrast, other candidate associations are detected primarily or preferentially after cross-linking and are also recovered in cross-linked AKiT datasets, consistent with more labile and/or indirect interactions. We therefore interpret ARK1–INCENP-A as the stable core CPC interaction in male gametogenesis, while still allowing that additional transient or weakly bound factors may exist. To reflect this nuance without weakening the main result, we have revised the manuscript to state that Plasmodium appears to deploy a reduced CPC with two core components during male gametogenesis (lines 915-920).

Line 180- Why did you choose a promoter swap here rather than a conditional KO?

Clarified (lines 234-236). Because these genes are essential and gametocytes are developmentally constrained once committed, promoter swap provides earlier gametocyte depletion to a late conditional knockout strategy.

Fig 3D – There appears to be many smaller AKiT5 puncta in the Pska-ARK1 panel than in the control cell. Is this signal specific or background? You should also show additional example images.

This image also shows clustering of all of the MTOCs/kinetochores etc to one side of the parasite. Is this a consistent pattern across all cells. This is reminiscent of some of the other segregation defects seen by others in asexual stage parasites.

Additional example images for ARK1 depletion phenotypes have been added in the Figure S1, Supplementary Data 2. Clustering of MTOCs may be due to the spindles that cannot properly elongate and "push" MTOCs apart.

Fig 3I – I like this analysis it seems to be good way to distil the data. It might however be better to start this section describing the measurements that were made referencing the images, then the explanation of the PCA.

Revised to introduce the underlying measured parameters more clearly, with examples in Figure S4G, followed by Figure 4D-G, then interpreting the PCA.

Line 240 – can you please clarify this statement. In Zeeshan etal there appears to be NDC80 staining in all stages of ookinetes and it also shows kinetochore alignments. In Brusini etal there is co-location of NUF2 with AKiT5. Are you suggesting here that the NDC80-NUF2 and AKiT5 are not associated? Co-location experiments and expansion microscopy would be need to support this claim.

We have previously shown that following fertilisation, AKiT-positive centromeric/kinetochore structures are detectable far before robust spindle-associated NUF2 foci become evident, with ordered meiotic kinetochore alignment becoming apparent only later (Brusini et al., 2022). This is consistent with eukaryotic hierarchical kinetochore assembly, in which inner kinetochore components are established before stable outer-kinetochore/spindle attachment states are resolved. Even though this is not the focus of the current study, we also show it here by both live and U-ExM, Figure 6A, B, Figure 7A and Figure S6A. We have not looked at NDC80 in Plasmodium therefore cannot comment on its localisation.

Fig 5A panel iii – there appears to be some puncta that are also decorating along the microtubules. This should be noted.

This has now been included (lines 311–315), alongside the point that the dominant meiotic feature is the appearance of end-on kinetochore attachment. Because these images are 3D reconstructions displayed as 2D projections, some puncta that appear to decorate spindle

microtubules likely represent end-on kinetochores that overlap with other spindle microtubules in the flattened view. This is now more clearly visible in Video S4. Supplementary Data 3 has now also been included for additional examples.

Line 260 – In this section where you use the ARK1 PAC's it would be useful to have more of an explanation of what the constructs are. This will help with the understanding and interpretation

We have updated Fig. S4F and S5E for clarification.

Figure 6 – Again it would be good to see additional example images of the patterns observed.

Additional example images for meiotic stages are now included in Supplementary Data 3.

Figure 7 B - the labels on the top of the panels on the right. Should be centred with tubulin/marker/ARK1 to make it clear this is refering to the colours in the merges below. You could also indicate that these are merged images.

Changed as recommended, now Figure S6B.

Fig 7- You see NUF 2 puncta in 7E at 6h and some accumulation already at 4 h. This is different to the NUF 2 pattern shown in 7A where NUF 2 is not observed as puncta until 8h.

We thank the reviewer for drawing attention to this point. The original time-course panel was designed to show a representative developmental series while simultaneously following three fluorophores (NUF2-mScI, AKiT5-mNG-3xHA and ARK1-mBFP2-2xTy) together with DIC imaging. Acquiring all four channels in the same representative cells necessarily constrains cell orientation, focal plane and signal intensity; under these conditions, early NUF2 signal can be weak and may not always resolve as distinct puncta in every cell.

In addition, we note that "time 0" in these experiments corresponds to activation of gametocytes, not the exact moment of fertilisation. As we cannot control the time it takes for a flagellum to find a macrogametocyte, fertilisation is slightly asynchronous across the population, meaning cell-to-cell variation is unavoidable.

To address this more clearly, we have now included Figure S6A, which shows multiple representative cells at 6 h post-fertilisation and explicitly illustrates this variability. At this stage, NUF2-mScI ranges from barely detectable signal to clearly resolved puncta, alongside AKiT5 and ARK1. The manuscript has been revised accordingly and now states that NUF2 kinetochore foci become clearly detectable typically between 4–6 h, rather than implying a later onset.

We therefore agree that the original panel could be misinterpreted, but we do not consider the datasets to be contradictory. Rather, they reflect a gradual accumulation of NUF2 at meiotic kinetochores, detectable after AKiT5 and persisting longer than ARK1 signal, which diminishes as ordered meiotic kinetochore configurations are established. In this context, the relative timing between the fluorophores is the key observation, rather than absolute time points within a partially asynchronous population.

Discussion – The phenotype observed in the ARK depleted males where you see a disconnection of the outer MTOC from the spindle and kinetochores is similar to what has been previously observed following deletion of NDC80. This leads to a defect in segregation of nuclei. Other, albeit milder. defects have also been observed following deletion of mini-chromosome maintenance complex binding-protein and schizont egress antigen-1. This should be discussed.

We now explicitly compare the ARK1 male phenotype with previously described MTOC–kinetochore-spindle uncoupling (lines 930-934) and place it in the context of other segregation phenotypes reported in malaria parasites.

We thank the reviewer again for these suggestions, which helped us make the manuscript more accessible and much clearer.

Closing statement

We are grateful to all three reviewers for their detailed and constructive comments. We believe the revised manuscript is substantially improved in clarity, methodological transparency, figure presentation, and conceptual balance. We hope that the revised version addresses the reviewers' concerns satisfactorily and we thank the reviewers again for helping us strengthen the manuscript.

RESPONSE TO REVIEWERS

We thank the editor and reviewers for their positive assessment of our revised manuscript. We are grateful that the reviewers found the revised version substantially improved and that the major concerns have now been addressed. In this final revision, we have focused on the remaining minor reviewer comments and editorial requirements. Specifically, we have corrected the spindle assembly checkpoint wording, added the suggested recent Toxoplasma gondii reference relating to early centromere-proximal replication, addressed the remaining figure and text corrections, and made minor typographical and formatting changes requested during final checks.

We respond to each reviewer below.

Sincerely,

Lorenzo Brusini

Reviewer #1:

I want to thank and congratulate all authors for resubmitting a rigorously revised manuscript. I have no further comments other than congratulations.

We thank Reviewer #1 for their positive assessment of our revised manuscript and are pleased that they have no further comments.

Reviewer #2:

The study offers a new perspective on mitosis in apicomplexan parasites. Using the Plasmodium berghei model, the authors demonstrated a flexible organization of the core mitotic machinery that adapts to the parasite's different life-stage forms. The revised manuscript is substantially improved; all major concerns have been properly addressed. The corrected study is exciting and contributes significantly to the cell biology of apicomplexan parasites and overall biology of eukaryotes.

We thank Reviewer #2 for their positive assessment of the revised manuscript and for recognising the significance of the study.

A few minor suggestions that authors may want to consider:

Lines 64-66: Please correct. The improper kinetochore attachment results in metaphase (not anaphase) arrest at SAC. Indeed, anaphase arrest is not achievable, but it is not related to SAC malfunction. Also, examination of Toxoplasma SAC confirmed the metaphase arrest using conditional downregulation of the SAC CDK-cyclin complex (Hawkins et al., 2021).

We have revised the relevant text to state that the spindle assembly checkpoint delays metaphase-to-anaphase progression, rather than referring to anaphase arrest. The revised wording now refers to the lack of a complete metaphase arrest in response to certain spindle poisons.

Line 886: Recent study in Toxoplasma demonstrated DNA replication machinery engagement to centromeric regions at the onset of S-phase, suggesting that centromeres are the first regions to replicate in Toxoplasma (PMID: 40590555). It indirectly supports the findings of this study.

We have added the recent Toxoplasma gondii study to the manuscript and revised the relevant paragraph to state that engagement of DNA replication machinery at centromeric regions at the onset of S-phase indirectly supports early centromere-proximal replication in apicomplexans. We have also retained the broader reference to centromere-first replication in other eukaryotes.

Reviewer #3:

The authors have produced a significantly improved manuscript which is easy to read and shows of this important new data. They have adequately addressed all of my comments and concerns from my first review. Below are a few minor comments/correction. Again congratulations on the work.

We thank Reviewer #3 for their positive assessment of the revised manuscript and for their remaining helpful comments.

Minor corrections

Fig 2 b sort 10? Or 10^6 in second line down.

We thank the reviewer for raising this point. We have checked Fig. 2B and confirm that "10" is the intended label.

Fig 3d why do you have the live cell panel as part of d? and not on its own as e

We thank the reviewer for this suggestion. We considered separating the live-cell images into a new panel. However, because these images directly support the same experimental point presented in Fig. 3D, we have retained them within the same panel to preserve the figure flow and maintain the direct link between the microscopy validation and the associated data.

Line 283-285 – remove the word "reduced"

This has been removed.

Fig7b. It would be better to give each panel its own letter as while related they are 4 different sets of data.

We have revised the organisation of this part of Fig. 7 by splitting the previous panel into two lettered panels, grouping the related datasets together with their corresponding microscopy validation. The figure legend and relevant text have been updated accordingly.

Closing statement

We thank the reviewers again for their constructive comments throughout the review process. We believe that the final revisions have improved the clarity and presentation of the manuscript, and we hope that the revised version is now suitable for publication in Nature Communications.
