# Supplementary material for: A modular chromosomal passenger complex rewires chromosome segregation in Plasmodium berghei
Source: Nat Commun. 2026 Jun 20;17:7788. doi: 10.1038/s41467-026-74542-7 (PMC13439184; doi:10.1038/s41467-026-74542-7)
Supplement: Supplementary file 4 [file 41467_2026_74542_MOESM2_ESM.pdf]

Description of Additional supplementary files

Supplementary Data 1. Quantitative U-ExM measurements of spindle, kinetochore and MTOC features during microgametogenesis.

Measurements from three-dimensional U-ExM reconstructions of non-activated and activated microgametocytes, including spindle, kinetochore and MTOC parameters as defined in the Methods. Each row represents a single cell.

Supplementary Data 2. Raw data underlying chromosome inheritance assays.

Measurements from flow cytometry and imaging-flow cytometry used to calculate blood-stage chromosome retention and paternal transmission in sexual stages. Each row corresponds to an individual sample/replicate.

Supplementary Data 3. ARK1 interactome during microgametogenesis.

Mass spectrometry enrichment of proteins co-purifying with ARK1-3xHA in male-only fd^{1-} gametocytes under native and sequential formaldehyde (FA) crosslinking conditions. Relative abundance values are shown as NSAF values alongside AKiT1-3xHA control immunoprecipitates.

Supplementary Data 4. Raw counts underlying exflagellation and ookinete conversion assays.

Counts used to calculate exflagellation rates and ookinete conversion efficiency for each genotype/condition.

Supplementary Data 5. Quantification of spindle and MTOC organisation during microgametogenesis.

Individual cell measurements for full-cell U-ExM projections of parental and mutant lines, including spindle poles, inner MTOCs, basal bodies, spindles and kinetochores.

Supplementary Data 6. Mosquito infection outcomes following ARK1 or INCENP-A depletion.

Enumeration of midgut and salivary gland infections per mosquito across biological replicates at 8, 14 and 22 days post-blood-meal, as well as mouse bite-back experiments.

Supplementary Data 7. Meiotic spindle and kinetochore morphology in zygotes/ookinetes.

Individual cell measurements for full-cell U-ExM projections of parental and mutant lines, including for spindle poles, MTOCs, spindles, and kinetochores.

Supplementary Data 8. ARK1 interactome during meiosis and mixed gametocytes.

Mass spectrometry enrichment of proteins co-purifying with ARK1-3xHA in female-only md^{4-} gametocytes 8h post-fertilisation and mixed *2.34* gametocytes populations 6 minutes post-

activation. Relative abundance values are shown as NSAF values alongside AKiT1-3xHA control immunoprecipitates.

Supplementary Data 9. Sources for detection of putative INCENP homologs in Apicomplexa.

Supplementary Data 10.

Full U-ExM 3D reconstructions and representative imaging datasets supporting the analysis of microgametogenesis in parental parasites.

Supplementary Data 11.

Full U-ExM 3D reconstructions and representative imaging datasets supporting the analysis of ARK1-depleted microgametocytes.

Supplementary Data 12.

Full U-ExM 3D reconstructions and representative imaging datasets supporting the analysis of meiotic spindle and kinetochore dynamics during zygote/ookinete development.

Supplementary Movie 1. Three-dimensional U-ExM reconstruction displaying the methodology used for analysis of microgametocyte spindle and kinetochore organisation. The cell is rotated on its axis, and channels are shown sequentially for AKiT5-mNG-3xHA-labelled kinetochores, DNA stained with SYTOX, microtubules labelled with α/β-tubulin, and spindle/basal-body MTOCs labelled with NHS ester.

Supplementary Movie 2. Three-dimensional U-ExM reconstruction of a parental microgametocyte at 6 min post-activation. The cell is rotated on its axis, and channels are shown sequentially for AKiT5-mNG-3xHA-labelled kinetochores, DNA stained with SYTOX, microtubules labelled with α/β-tubulin, and spindle/basal-body MTOCs labelled with NHS ester.

Supplementary Movie 3. Three-dimensional U-ExM reconstruction of a PSKA-ark1 microgametocyte at 6 min post-activation. The cell is rotated on its axis, and channels are shown sequentially for AKiT5-mNG-3xHA-labelled kinetochores, DNA stained with SYTOX, microtubules labelled with α/β-tubulin, and spindle/basal-body MTOCs labelled with NHS ester.

Supplementary Movie 4. Three-dimensional U-ExM reconstruction of a parental developing ookinete at 6 h of development. The cell is rotated on its axis, and channels are shown sequentially for AKiT5-mNG-3xHA-labelled kinetochores, DNA stained with SYTOX, microtubules labelled with α/β-tubulin, and spindle/basal-body MTOCs labelled with NHS ester.

Supplementary Movie 5. Three-dimensional U-ExM reconstruction of a parental developing ookinete at 8 h of development. The cell is rotated on its axis, and channels are shown sequentially for AKiT5-mNG-3xHA-labelled kinetochores, DNA stained with SYTOX, microtubules labelled with α/β-tubulin, and spindle/basal-body MTOCs labelled with NHS ester.

Supplementary Movie 6. Three-dimensional U-ExM reconstruction of a PCLAG-ark1 developing ookinete at 8 h of development. The cell is rotated on its axis, and channels are shown sequentially for AKiT5-mNG-3xHA-labelled kinetochores, DNA stained with SYTOX, microtubules labelled with α/β-tubulin, and spindle/basal-body MTOCs labelled with NHS ester.

Supplementary Movie 7. Three-dimensional U-ExM reconstruction of a fully developed parental ookinete at 24 h of development. The cell is rotated on its axis, and channels are shown sequentially for AKiT5-mNG-3xHA-labelled kinetochores, DNA stained with SYTOX, microtubules labelled with α/β-tubulin, and spindle/basal-body MTOCs labelled with NHS ester.

Supplementary Movie 8. Three-dimensional U-ExM reconstruction of a fully developed PCLAG-ark1 ookinete at 24 h of development. The cell is rotated on its axis, and channels are shown sequentially for AKiT5-mNG-3xHA-labelled kinetochores, DNA stained with SYTOX, microtubules labelled with α/β-tubulin, and spindle/basal-body MTOCs labelled with NHS ester.
