# Supplementary material for: A modular chromosomal passenger complex rewires chromosome segregation in Plasmodium berghei
Source: Nat Commun. 2026 Jun 20;17:7788. doi: 10.1038/s41467-026-74542-7 (PMC13439184; doi:10.1038/s41467-026-74542-7)

Figure S3.

B

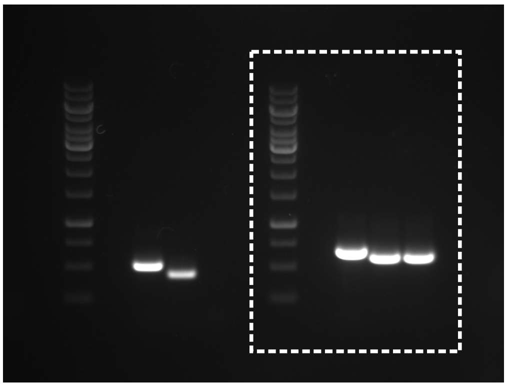

G

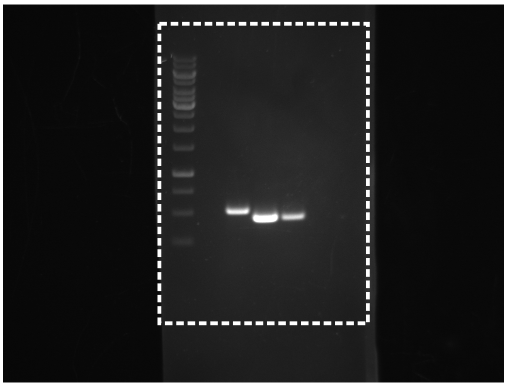

H

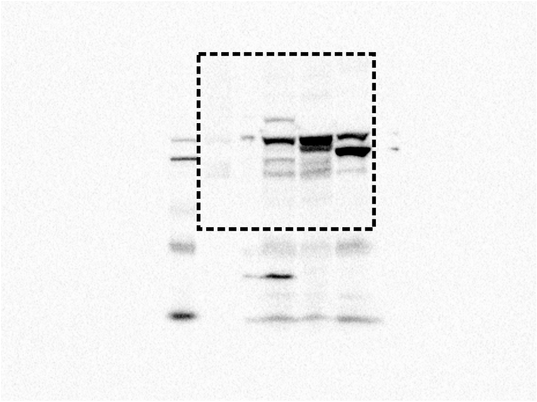

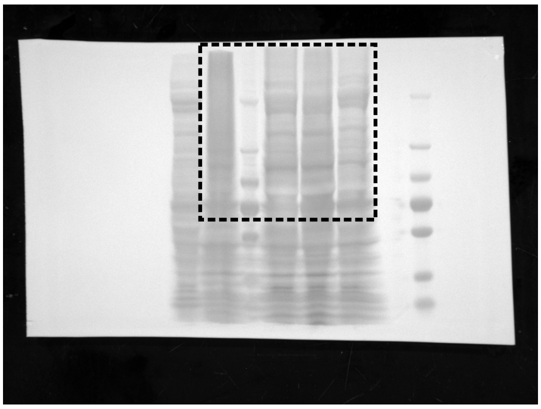

Figure S4.

C

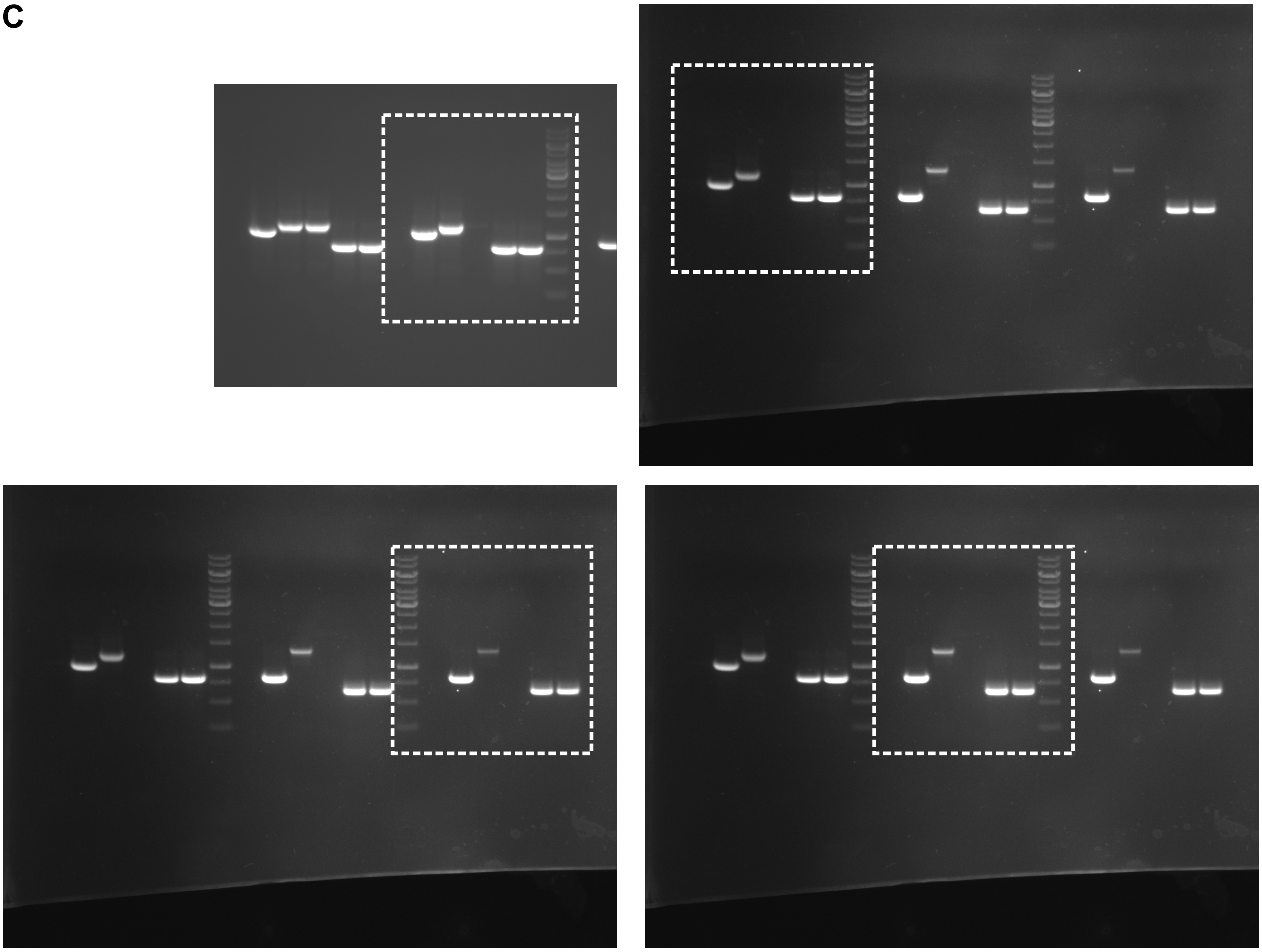

D

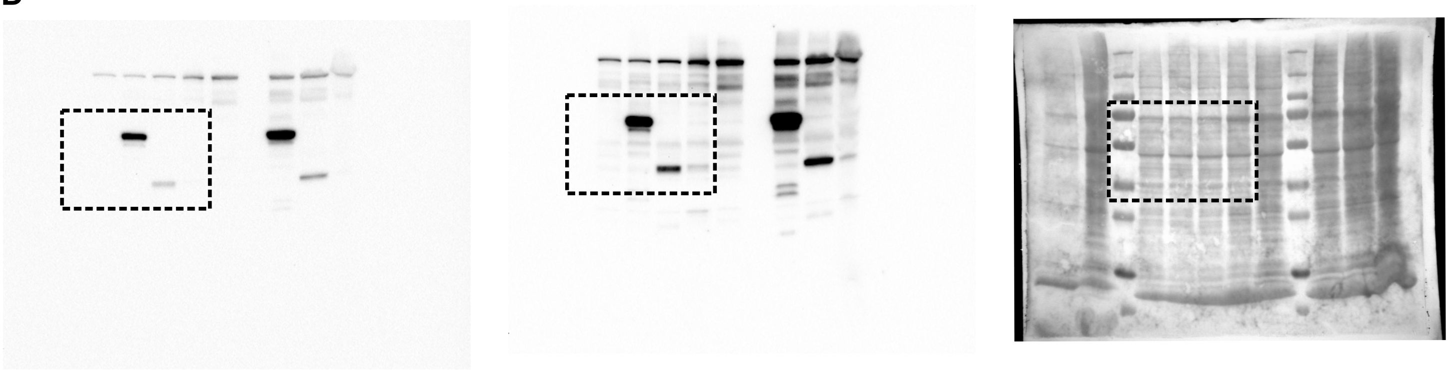

E

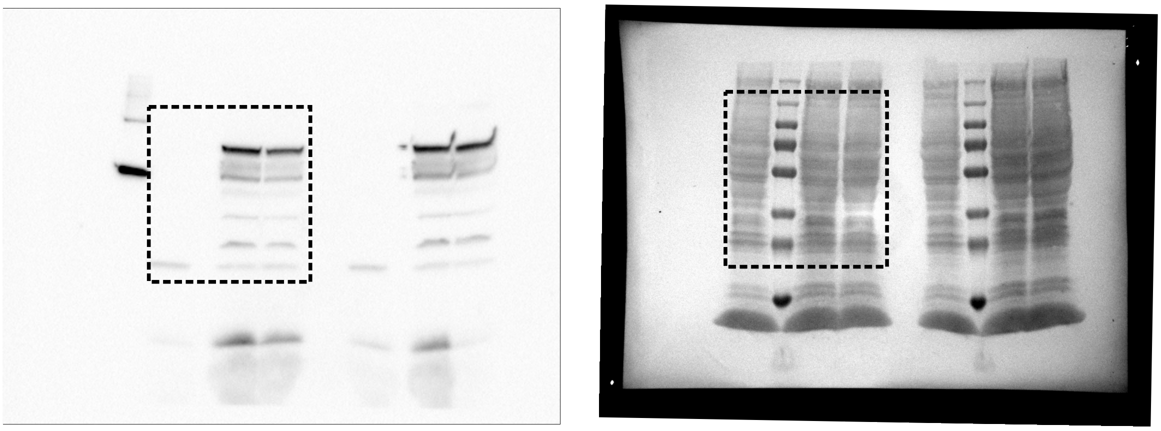

Figure S5.

F

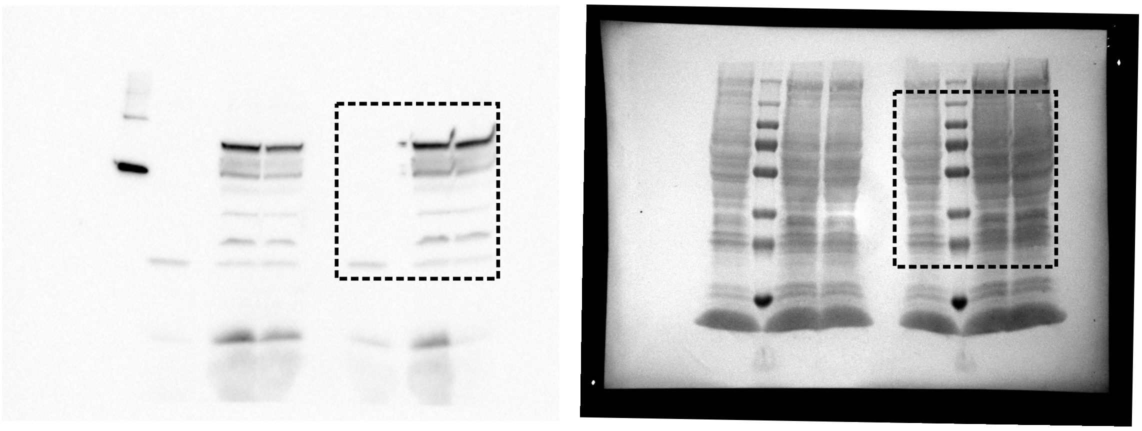

Figure S6.

C

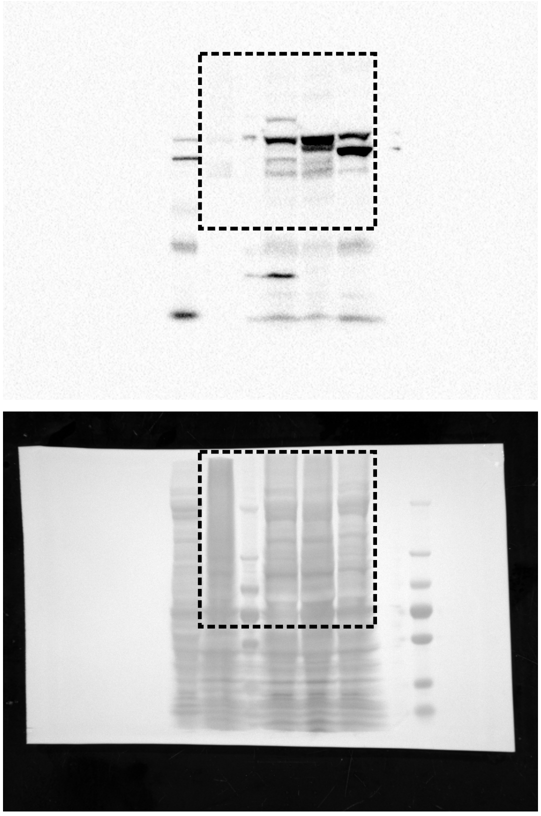

H

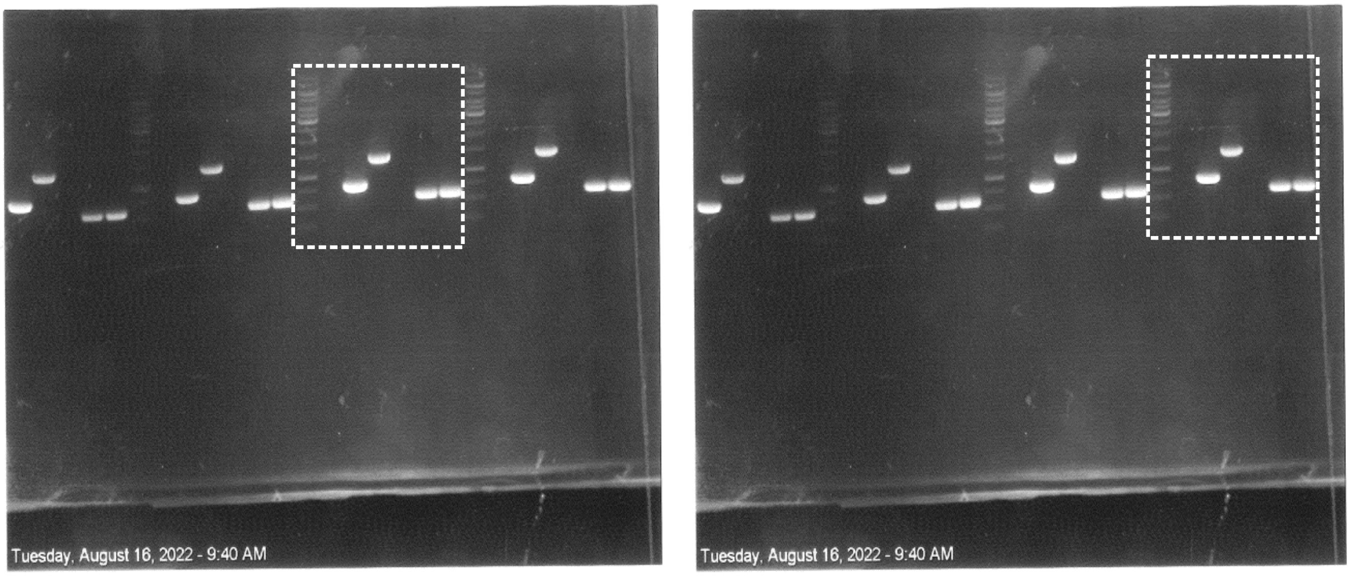

Supplement: Supplementary file 8 [file 41467_2026_74542_MOESM25_ESM.zip › Source_Data_blots.pdf]
